# *Polygonatum sibiricum* polysaccharides enhance pancreatic β-cell function in diabetic zebrafish by mitigating mitochondrial oxidative damage via the AMPK-SIRT1 pathway

**DOI:** 10.3389/fnut.2025.1601490

**Published:** 2025-05-09

**Authors:** Fan Lin, Wenjing Yu, Ping Li, Shuyao Tang, Yitong Ouyang, Liya Huang, Di Wu, Shaowu Cheng, Zhenyan Song

**Affiliations:** ^1^School of Integrated Chinese and Western Medicine, Hunan University of Chinese Medicine, Changsha, China; ^2^Key Laboratory of Hunan Province for Integrated Traditional Chinese and Western Medicine on Prevention and Treatment of Cardio-Cerebral Diseases, Hunan University of Chinese Medicine, Changsha, China

**Keywords:** *Polygonatum sibiricum* polysaccharides, mitochondrial oxidative damage, pancreatic β-cells, AMPK-SIRT1 pathway, zebrafish, network pharmacology

## Abstract

**Background:**

Mitochondrial oxidative damage in pancreatic β-cells is a key contributor to diabetes pathogenesis, particularly under hyperglycemic conditions. *Polygonatum sibiricum* polysaccharides (PSP) have demonstrated potential anti-diabetic effects; however, their precise mechanism, particularly through the AMPK-SIRT1 pathway, remains unclear.

**Methods:**

A diabetic zebrafish model was established by exposure to 2% glucose for 28 days. Zebrafish were divided into control, model, low-dose PSP (50 μg/mL), medium-dose PSP (100 μg/mL), high-dose PSP (200 μg/mL), and metformin groups. Behavioral, biochemical, and molecular analyses were performed to assess β-cell function, mitochondrial oxidative damage, and inflammation. Network pharmacology analysis was used to predict PSP targets, and molecular docking validated key protein interactions. Immunofluorescence and Western blotting (WB) were conducted to examine apoptosis-related protein expression.

**Results:**

*Polygonatum sibiricum* polysaccharides significantly improved zebrafish swimming behavior, reduced blood glucose and fructosamine levels, and enhanced ATP production (*p* < 0.01). Antioxidant enzyme activities (SOD, CAT) increased, while oxidative stress markers (MDA) and inflammatory cytokines (IL-1β, IL-6, TNF-*α*) decreased (*p* < 0.01). PSP treatment downregulated Cycs expression, alleviating mitochondrial damage. Moreover, PSP upregulated AMPK and SIRT1 expression (*p* < 0.01), along with downstream regulators PGC-1α and Nrf1/2 (*p* < 0.01), confirming AMPK-SIRT1 pathway activation. Network pharmacology identified 389 shared targets between PSP and diabetes-related pathways, implicating key mechanisms of inflammation, insulin resistance, and mitochondrial dysfunction. Molecular docking demonstrated strong PSP binding affinities to AMPK and SIRT1. Immunofluorescence and WB analyses showed reduced cleaved caspase-3 levels and apoptosis in pancreatic β-cells following PSP treatment (*p* < 0.01).

**Conclusion:**

*Polygonatum sibiricum* polysaccharides protects pancreatic β-cell function in diabetic zebrafish by mitigating mitochondrial oxidative stress and apoptosis via AMPK-SIRT1 pathway activation. Network pharmacology and molecular docking further highlight PSP’s potential as a multi-target therapeutic agent for diabetes.

## 1 Introduction

In recent years, with improvements in living standards and changes in lifestyle, diabetes has become a major global health concern. According to statistics, the prevalence of diabetes among adults in China reached 12.8% in 2023, while approximately 529 million people worldwide are affected. By 2030, this number is projected to rise to 10.2% of the global population (1). The primary cause of diabetes is the dysfunction or loss of pancreatic β-cells, leading to insufficient insulin secretion and subsequent metabolic imbalances. Pancreatic β-cell impairment is considered a central pathophysiological event in the onset and progression of diabetes (2, 3). Among the various mechanisms contributing to β-cell dysfunction, mitochondrial oxidative damage plays a pivotal role, particularly under chronic hyperglycemic conditions. Excess glucose levels result in excessive mitochondrial reactive oxygen species (ROS) production, causing mitochondrial DNA mutations, disruption of the electron transport chain, and impaired ATP synthesis, ultimately leading to β-cell apoptosis (4).

The AMPK-SIRT1-PGC1α signaling axis is a crucial regulator of cellular energy metabolism and plays a key role in maintaining mitochondrial homeostasis in β-cells. Upon activation under conditions of energy stress, AMPK triggers a cascade involving the phosphorylation of PGC1α (Ser538) and the upregulation of SIRT1 expression. SIRT1 subsequently enhances the transcriptional activity of PGC1α through deacetylation at Lys778, thereby promoting the expression of mitochondrial biogenesis-related genes, such as NRF1 and TFAM (5). Studies have shown that AMPK activation mitigates diabetes-related metabolic disorders and preserves β-cell function. Specifically, AMPK-induced SIRT1 upregulation enhances cellular resistance to oxidative stress and inflammation via deacetylation mechanisms. Furthermore, SIRT1-mediated deacetylation of PGC-1α promotes mitochondrial biogenesis, ultimately improving mitochondrial function (6). Recent studies have demonstrated that widely used antidiabetic agents, such as metformin, enhance β-cell mitochondrial function through this pathway (7, 8). Although natural compounds like resveratrol and luteolin are well-characterized activators of the AMPK-SIRT1 pathway, the potential of PSP to modulate this pathway remains underexplored in global research (9–11).

In recent years, diabetes has emerged as a global public health crisis, and research into its pathogenesis and therapeutic strategies faces dual challenges (12). *Polygonatum sibiricum* (Huangjing) is a nutrient-rich edible plant widely consumed for its health benefits. It contains a variety of bioactive compounds, including polysaccharides, flavonoids, and saponins, which contribute to its functional properties. Among these, *P. sibiricum* polysaccharides (PSP) have been extensively studied for their potential health-promoting effects. Modern pharmacological studies have shown that PSP can improve glucose and lipid metabolism in diabetic animal models through mechanisms such as scavenging free radicals and inhibiting the NF-κB inflammatory pathway. PSP exhibits significant effects in antioxidant activity, anti-inflammatory action, and improving glucose metabolism (13).

The zebrafish serves as a robust translational model for diabetes research, owing to its high genetic tractability, physiological parallels to human metabolic pathways, and substantial genomic conservation (approximately 70% of disease-related genes are homologous). These attributes, combined with its experimental versatility, make it a widely adopted system for investigating diabetic mechanisms and therapeutic interventions (14, 15). This study employs a high-glucose-induced zebrafish model to evaluate the protective role of PSP on β-cell mitochondrial function. Key techniques include behavioral tracking, biochemical assays, immunostaining, qPCR, network pharmacology, and molecular docking. We focus on the AMPK-SIRT1 signaling axis and associated antioxidant, apoptotic, and inflammatory markers to elucidate PSP’s therapeutic mechanism.

## 2 Materials and methods

### 2.1 Reagents and instruments

Metformin hydrochloride (D9351) was purchased from Solarbio Company; glucose (50–99-7) was obtained from China National Pharmaceutical Group Chemical Reagent Co., Ltd.; the reverse transcription cDNA kit (A5001) was sourced from Promega, United States; the Superoxide Dismutase (CuZn-SOD/Mn-SOD) assay kit (hydroxylamine method) (E-BC-K022-M), Catalase (CAT) colorimetric assay kit (E-BC-K031-M), Malondialdehyde (MDA) colorimetric assay kit (E-BC-K025-M), and ATP content chemiluminescent assay kit (E-BC-F002) were purchased from Wuhan Elabscience Biotechnology Co., Ltd.; the Zebrafish Fructosamine (FRA) ELISA kit (KT5402-A) was obtained from Jiangsu KET Biological Technology Co., Ltd.; the Fast antibody dye-based quantitative PCR premix (MQ10401S) was purchased from Mona Biotechnology Co., Ltd.; primers were synthesized by Shanghai Shenggong Biotechnology Co., Ltd. Cleaved-caspase 3 antibody (GB11532), Alexa Fluor 488 labeled goat anti-rabbit IgG (GB25303); One-step TUNEL cell apoptosis detection kit (TMR red fluorescence) (G1502-50T) were purchased from Wuhan Servicebio Biotechnology Co., Ltd.

We obtained the following: The Roche Blood Glucose Meter Excellence Precision Model (Roche Diagnostics Products Shanghai Co., Ltd.); ZebraTower Automated Zebrafish Analysis System Adult Observation Tower (ViewPoint, France); Fishbio-d5 Zebrafish Double-Row Five-Layer Circulating Rearing Rack (Shanghai Fexi Biotechnology Co., Ltd.); Medifuge™ Small Desktop Centrifuge (Thermo Fisher Scientific, United States); Cytation3 Multifunctional Microplate Reader (BioTek, United States); T100™ Real-Time Fluorescent Quantitative PCR (Real-time qPCR) System (Bio-Rad, United States); HM355S Paraffin Microtome (Thermo Fisher Scientific, United States); Microscopic Imaging System (Motic, China).

### 2.2 Animals

Male AB strain zebrafish, aged 6–7 months, were purchased from the National Zebrafish Resource Center and maintained in a zebrafish breeding system. Rearing conditions included pH (6.8–8.5), temperature (24–28°C), alkalinity (50–100 mg/L), salinity (0.5–2.0 g/L), dissolved oxygen (>4 mg/L), and a light/dark cycle of 14/10 h. The zebrafish were fed twice daily. All procedures were approved by the Ethics Committee of Hunan University of Chinese Medicine (Approval No. LLBH-202311100013).

Seven to eight month old AB strain zebrafish were randomly selected and bred in a breeding tank, fed with Artemia. Based on the results of acute toxicity testing, the concentrations were set as low, medium, and high doses at 50, 100, and 200 μg/mL, respectively. Additionally, positive control drug metformin and model and control groups were included. The modeling lasted for 14 days. A total of 240 adult zebrafish aged 6–7 months were randomly assigned to the control group, model group, and three PSP dose groups (50, 100, and 200 μg/mL), as well as the metformin group (30 μg/mL), with 40 fish in each group. The control group was maintained in a standard aquatic environment, while the model group, PSP groups, and metformin group were exposed to a 2% glucose solution (10 fish/1500 mL). They were fed with three times the standard amount of Artemia daily, continuously exposed for 28 days to induce a hyperglycemic model. Starting from day 14, the low, medium, and high doses of PSP and metformin groups received continuous treatment for 14 days.

### 2.3 Acute toxicity experiment of PSP

During the acute toxicity assay, larvae were maintained in 6-well plates at a density of 10 embryos per well (3 mL solution/well). Water quality (pH, dissolved oxygen, temperature) was monitored daily, and 50% of the solution was replaced every 24 h to ensure optimal conditions. The groups were set as control, 50 μg/mL PSP, 100 μg/mL PSP, 200 μg/mL PSP, and 400 μg/mL PSP. The experiment lasted for 96 h, with observations and recordings of zebrafish hatching and developmental conditions taken every 8 h. The PSP concentrations (50–400 μg/mL) were selected using a tiered approach: lower doses (50–200 μg/mL) aligned with preliminary tests and environmental exposure data, while the highest dose (400 μg/mL) served as a conservative safety margin for extreme toxicity assessment (16–18).

### 2.4 Measurement of body weight, blood glucose, and fructosamine

Prior to body weight and blood glucose measurements, zebrafish were fasted for 12 h. The zebrafish to be tested were collected, anesthetized with an ice bath, surface water was removed, and they were placed on a tray. Body weight was recorded and measured both before modeling and prior to blood collection. The tail was amputated for blood collection, and blood glucose levels were quickly measured using a Roche blood glucose meter. Zebrafish tissue was collected and homogenized in an appropriate volume of physiological saline under ice bath conditions. The mixture was centrifuged at 3000 rpm for 10 min, and the supernatant was collected. Fructosamine was measured using undiluted supernatant per ELISA kit protocol.

### 2.5 Behavioral analysis of adult fish

Following the method described by Yu et al. (19), behavioral testing was conducted on zebrafish at the end of the 28-day intervention period. Zebrafish were observed using the ZebraTower Adult Observation Tower to record their locomotor behavior. The total swimming distance over a 60-s period and the swimming speed (high speed >5 cm/s, moderate speed 2–5 cm/s, low speed <2 cm/s) were recorded and statistically analyzed using the zebrafish behavioral analysis system. Adult zebrafish subjected to behavioral analysis were subsequently used for blood glucose measurement to ensure cohort consistency.

### 2.6 Biochemical indexes measurement

After behavioral testing, zebrafish were anesthetized with an ice bath. The pancreas was dissected according to the zebrafish anatomical diagram and homogenized in an appropriate volume of PBS under ice bath conditions. Tissues were homogenized in PBS (1:9w/v) and centrifuged (3,000 rpm, 10 min). Supernatants were diluted 1:10 for SOD/CAT and 1:5 for MDA/ATP. The activities of SOD, CAT, MDA, and ATP content were measured following the instructions provided with the biochemical assay kits.

### 2.7 HE staining

Zebrafish pancreatic and intestinal tissues were fixed in 4% paraformaldehyde, embedded in paraffin, sectioned, and baked. The sections were deparaffinized, stained with hematoxylin for 10 min, differentiated with hydrochloric alcohol, blued, stained with eosin for 2 min, and subjected to gradient dehydration. The sections were then cleared with xylene and mounted. Morphological and structural changes in the pancreatic tissue were observed under an optical microscope. Image analysis was performed using ImageProPlus 6.0 software.

### 2.8 RNA extraction and quantitative real-time PCR analysis

Zebrafish pancreatic tissue was collected, and the tissue from every five zebrafish was placed in a 1.5 mL EP tube for total RNA extraction using the Trizol method. After measuring the RNA concentration using a multifunctional microplate reader, cDNA was synthesized using a reverse transcription kit. The reaction program was as follows: 95°C for 5 min; 95°C for 30 s; 58°C for 30 s, for 40 cycles. GAPDH was used as the reference gene, and data analysis was performed using the 2^-△△Ct^ method. The primers used were designed and synthesized by Sangon Biological Engineering (Shanghai) Co., Ltd. (Table 1).

**Table 1:** PCR primer sequences.

Primer	Forward primer (5′ → 3′)	Reverse primer (3′ → 5′)	Length/bp
GAPDH	TGTGGAGTCTACTGGTGTCTTC	GGCATTGCTTACAACTGTGAGA	166
prkaa1	GCCTCCAGCTCTACCAAGTG	AGAGCCTTCCGCCACTTTAC	167
SIRT1	CACAGAGCCATGAAGCAGGA	CACGATCACGTCACAATCGC	156
Pgc-1α	GCGTGGGACAGGTGTAATCA	TTGGCCTCATTTTCCTCATCT	112
Nrf1	ACTGGAGAATGTGGTGAGGAA	CATCAATAGTCAGCGGAGGAAG	125
Nrf2	TGAAGCAGACGGAGGAGGAG	AAGGTGGAGCGGAGGTGTT	170
pck2	CCCCGGAACGTCCCTAAAAA	CCAGGATTTCCCATGCCAGT	132
Cycs	AATGCTCTCAGTGTCACA	GTCGCTGGAATGTACCTT	202
IL-1β	GCTGCTGTTCTTCAGGAAGGAGAC	TCCACCATCTGCGAATCTTCATACG	108
IL-6	GTCTGCTACACTGGCTACACTCTTC	CGTCCACATCCTGAACTTCGTCTC	110
TNF-α	GCGTTGAAGATGTTGAAGGAGA	CTAGAGACTGGCAGACGGAAT	123
bax	TACTTTGCCTGTCGCCTTGT	AGCGAGGAAAACTCCGACTG	112
Caspase 3	TGAGTCCACCTCCAACATGC	ACAGCTGCTGACGTTCTCAA	174
bcl2	AACCGACTCTTTCCTGCTCG	TTCAGAGTTGTTCCCTCCGC	138

### 2.9 Network pharmacology analysis

The active ingredients of *P. sibiricum* were retrieved from the TCMSP database^1^, with screening criteria set at oral bioavailability (OB) ≥ 30% and drug-likeness (DL) ≥ 0.18 to ensure pharmacological relevance. Potential targets of the compounds were predicted using Swiss Target Prediction^2^, with a probability threshold of >0.5 to enhance prediction reliability.

Diabetes-related targets were obtained from the GeneCards database^3^ by searching for “diabetes,” and only targets with a relevance score ≥ median were retained to prioritize high-confidence associations. Common targets between *P. sibiricum* and diabetes were identified through Venn analysis^4^.

The protein–protein interaction (PPI) network was constructed using STRING^5^ with a confidence score cutoff ≥ 0.4 to ensure robust interactions. Functional enrichment analysis, including Gene Ontology (GO) and Kyoto Encyclopedia of Genes and Genomes (KEGG) pathways, was performed using DAVID^6^, with a significance threshold of *p* < 0.05. Networks were visualized using Cytoscape 3.10.2.

### 2.10 Molecular docking

The structure was obtained by searching “*Polygonatum sibiricum* polysaccharides” in SciFinder^7^ and downloading the corresponding SDF files. The PDB files for “AMPK” and “SIRT1” were downloaded from the PDB database^8^. The PDB files of the relevant proteins and the SDF files of the small molecules were input into the CB-Dock2 website^9^ for molecular docking, and the Vina scores and docking diagrams were generated. The resulting Vina scores were uploaded to the Microbioinformatics platform^10^ for further analysis.

### 2.11 TUNEL staining

Paraffin sections were dewaxed with xylene, and treated with DNase- and proteinase K-free solution (20 mg/L) for 30 min at room temperature. After washing with PBS three times, TUNEL detection solution was added, followed by incubation at room temperature in the dark for 60 min. The sections were washed with PBS three times, and mounted with an anti-fade reagent containing DAPI. Imaging was performed using a panoramic scanner, and three fields of view were selected from each sample to calculate the TUNEL-positive cell rate.

### 2.12 Immunofluorescence detection of cleaved-caspase 3 levels

Paraffin sections were baked, permeabilized, and subjected to antigen retrieval. After blocking, primary antibody incubation was performed overnight at 4°C. The following day, the sections were incubated with fluorescent secondary antibody for 1 h in the dark. After staining, the slides were mounted with an anti-fluorescence quenching agent containing DAPI. Imaging was performed using a whole-slide scanner, and the average fluorescence intensity of each group was analyzed using Image J.

### 2.13 Western blot analysis

Tissues (100 mg) were homogenized in RIPA lysis buffer and incubated at 4°C, followed by sonication and centrifugation at 12,000 rpm for 15 min (centrifuge radius: 5 cm). The supernatant was collected, and protein concentration was measured using a BCA protein assay kit. A loading buffer with a protein concentration of 2 g/L was prepared and heated at 100°C for 20 min. A total of 20 μg of protein was loaded onto a 10% SDS-PAGE gel, with electrophoresis run at 80 V for 30 min, followed by 120 V for 60 min. Proteins were transferred to a 0.45 μm PVDF membrane by wet transfer for 120 min. The membrane was blocked with 5% skim milk at room temperature for 1 h. The primary antibody against cleaved-caspase3 (1:8000) was incubated overnight at 4°C, followed by incubation with the corresponding secondary antibody (1:10,000) for 1 h at room temperature. Protein bands were detected using ECL chemiluminescent substrate and analyzed with Image Lab software for densitometric analysis.

### 2.14 Statistical analysis

Experimental data are expressed as mean ± standard deviation, and statistical graphs were generated using Prism GraphPad 8.0. Data analysis was performed using SPSS 25.0. For multiple group comparisons, one-way ANOVA was used. If variances were homogeneous, the LSD method was applied; if variances were heterogeneous, the Tamhane’s T2 method was used. A *p*-value of less than 0.05 was considered statistically significant.

## 3 Results

### 3.1 Acute toxicity experiment of PSP in zebrafish embryos

**Figure 1:**
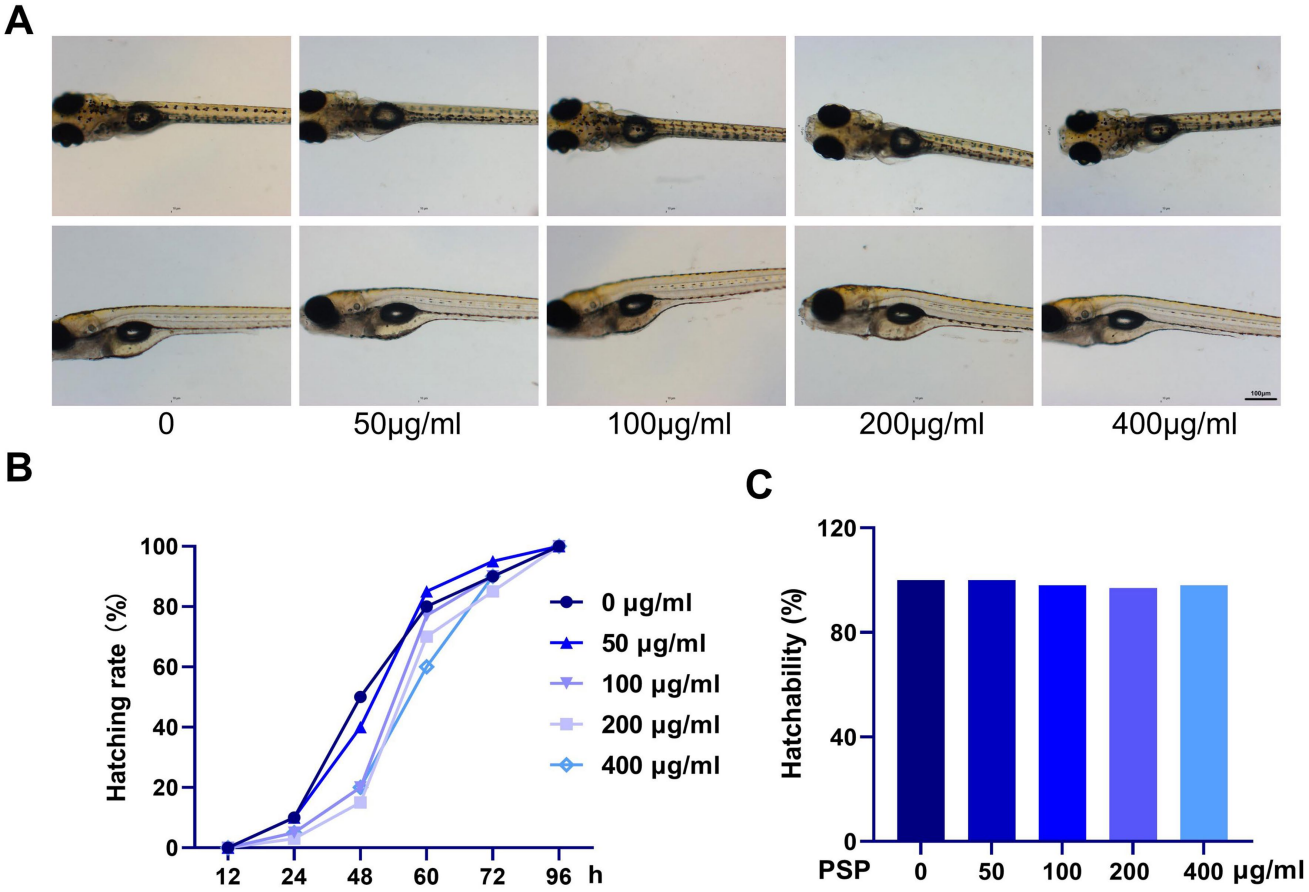
Effects of *Polygonatum sibiricum* polysaccharides (PSP) on acute toxicity in zebrafish. **(A)** Morphology of zebrafish larvae after treatment with various concentrations of PSP (96 h post-fertilization). **(B)** Hatching time of fertilized eggs following PSP treatment. **(C)** Hatching rate of fertilized eggs after PSP treatment. *N* = 100.

Zebrafish embryos were exposed to PSP from fertilization until hatching was completed. The results showed that exposure to 50, 100, 200, and 400 μg/mL PSP did not affect the normal hatching process of the embryos. The morphology of the zebrafish larvae across all treatment groups was consistent with that of the control group, and no abnormal behaviors were observed (Figure 1A). The hatching rate was 100% in all groups, with slight differences in the timing of hatching observed, though all embryos successfully hatched within 96 h post-fertilization (Figures 1B,C).

### 3.2 Behavioral analysis of zebrafish

**Figure 2:**
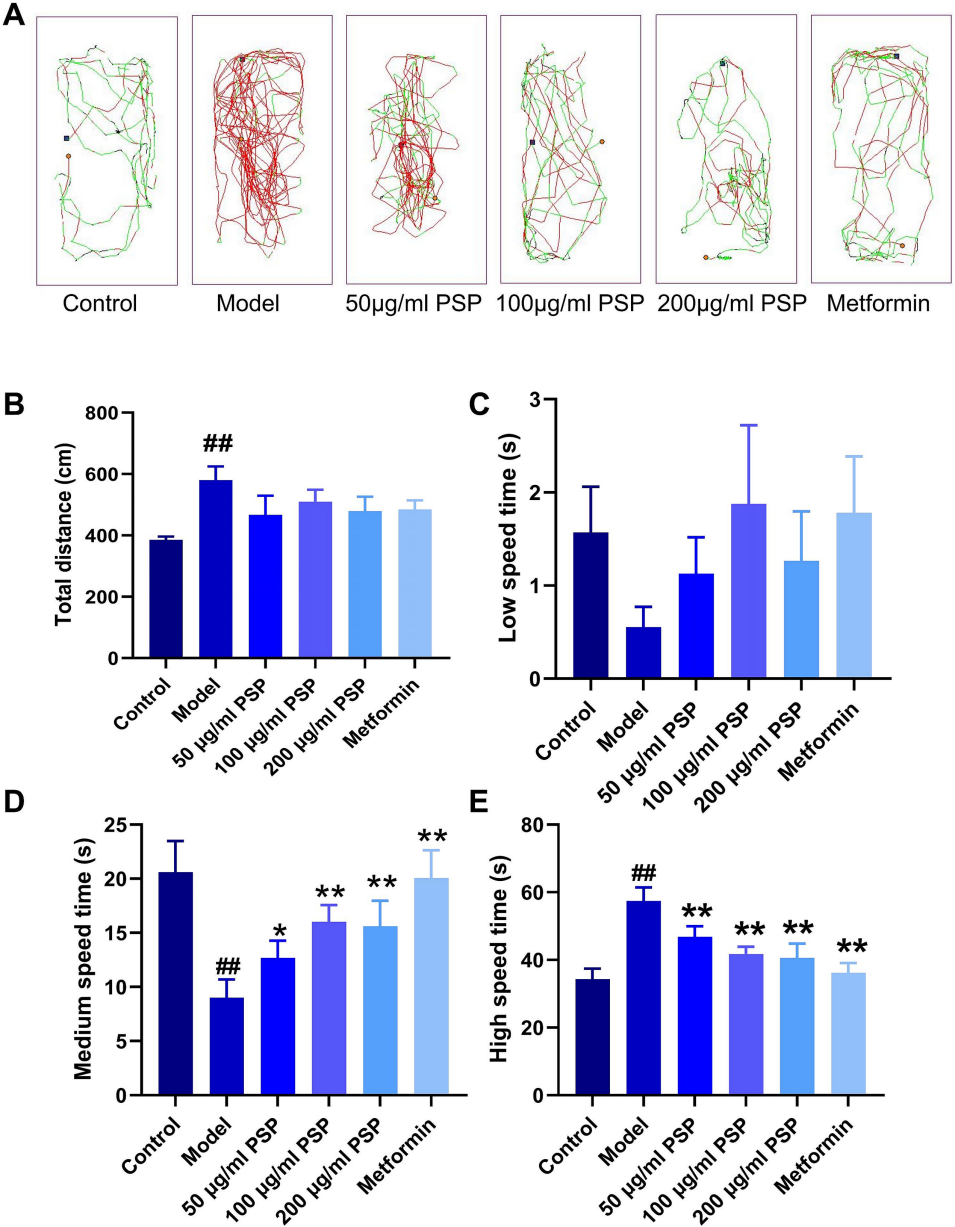
Effects of PSP on zebrafish behavior. **(A)** Movement trajectories of zebrafish in each treatment group. **(B)** Total movement distance of zebrafish in each group after PSP treatment (60 s). **(C)** Duration of slow movement in zebrafish in each group after PSP treatment. **(D)** Duration of moderate movement in zebrafish in each group after PSP treatment. **(E)** Duration of fast movement in zebrafish in each group after PSP treatment. ^##^*p* < 0.01 vs. Control; *p < 0.05, **p < 0.01 vs. Model; *N* = 10.

The total swimming distance of zebrafish in the model group was significantly increased compared to the control group (*p* < 0.01), while the time spent swimming at moderate speed was notably reduced (*p* < 0.01), and the time spent swimming at high speed was significantly increased (*p* < 0.01). In contrast, the PSP-treated groups (all doses) and the metformin group showed a reduction in total swimming distance compared to the model group, though these differences were not statistically significant (*p* > 0.05). Additionally, the time spent swimming at moderate speed was significantly increased (*p* < 0.05, *p* < 0.01), while the time spent swimming at high speed was significantly decreased (*p* < 0.01) in the PSP-treated groups and metformin group (Figure 2).

### 3.3 Effects of PSP on body weight and biochemical markers in a zebrafish diabetes model

**Figure 3:**
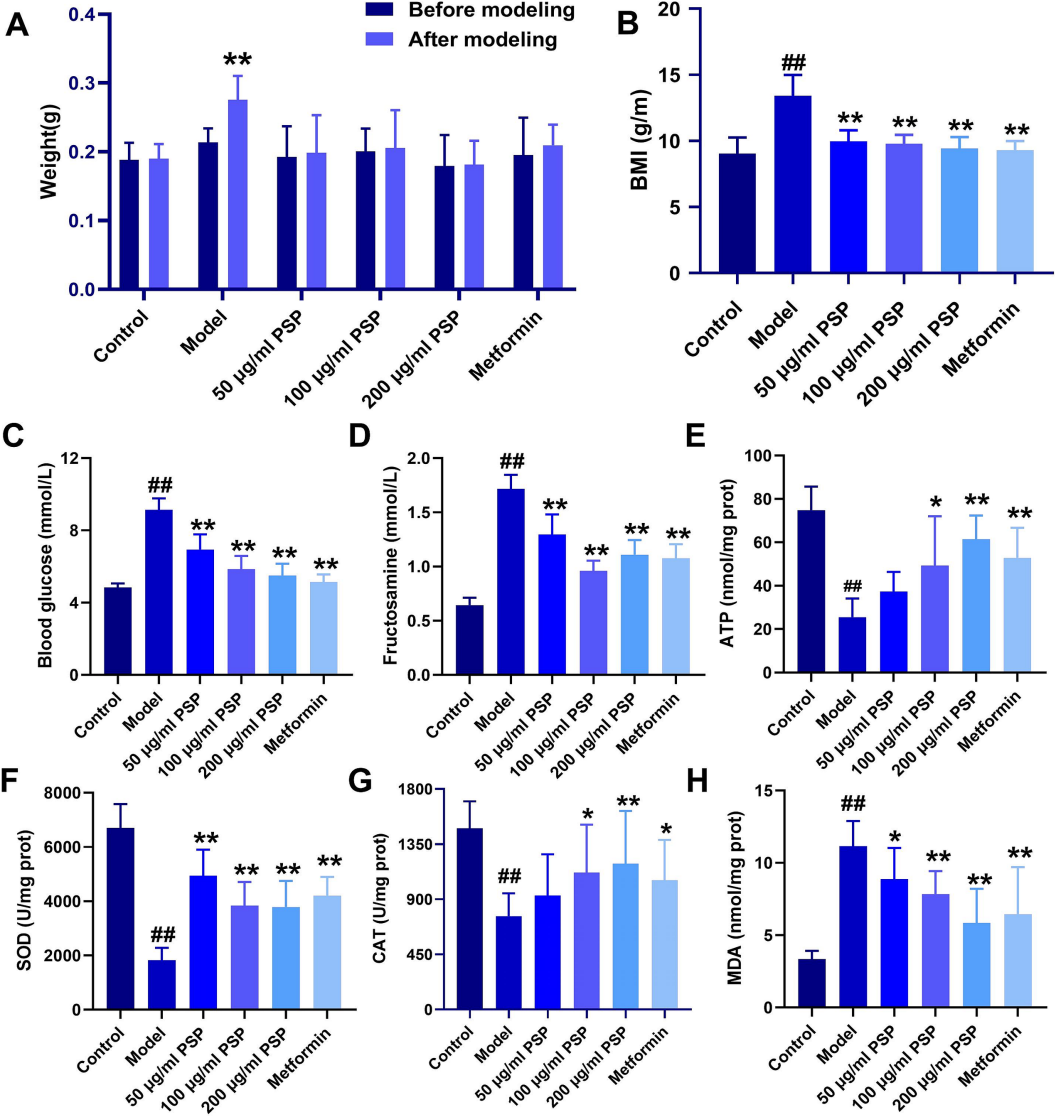
Effects of PSP on body weight, blood glucose, and antioxidant activity in a high-glucose-induced zebrafish model. **(A)** Weight changes in zebrafish before and after modeling. **(B)** Body mass index (BMI) of zebrafish in each group. **(C)** Blood glucose (mmol/L) levels. **(D)** Fructosamine (mmol/L) content. **(E)** ATP (nmol/mg protein) content. **(F)** SOD (U/mg protein) activity. **(G)** CAT (U/mg protein) activity. **(H)** MDA (nmol/mg protein) activity. ^##^*p* < 0.01 vs. Control; *p < 0.05, **p < 0.01 vs. Model; *N* = 10.

Body weight measurements taken before and after treatment revealed a significant increase in the model group compared to baseline (*p* < 0.01), while no significant changes in body weight were observed in the other groups (*p* > 0.05). The body mass index (BMI) was significantly higher in the model group compared to the control group (*p* < 0.01). In contrast, the BMI of zebrafish in the PSP-treated groups (50, 100, and 200 μg/mL) and the metformin group showed significant reductions compared to the model group (*p* < 0.01) (Figures 3A,B).

Blood glucose levels and fructosamine content were significantly elevated in the model group compared to the control group (*p* < 0.01). Treatment with PSP at 50 μg/mL, 100 μg/mL, 200 μg/mL and metformin significantly reduced both blood glucose levels and fructosamine content (*p* < 0.01), suggesting that PSP exerts a clear hypoglycemic effect (Figures 3C,D).

Biochemical analysis showed that the ATP content, SOD and CAT activities in the model group were significantly decreased (*p* < 0.01), whereas MDA activity was significantly increased (*p* < 0.01). In the 50 μg/mL PSP-treated group, ATP content and CAT activity remained unchanged (*p* > 0.05), but SOD activity was significantly increased (*p* < 0.01), and MDA activity was significantly decreased (*p* < 0.05). In the 100 μg/mL, 200 μg/mL PSP-treated groups and metformin group, ATP content, SOD and CAT activities were significantly increased (*p* < 0.05, *p* < 0.01), while MDA activity was significantly decreased (*p* < 0.01), indicating that PSP demonstrates strong antioxidant properties (Figures 3E–H).

### 3.4 Effects of PSP on pancreatic and intestinal pathology in zebrafish diabetes model

**Figure 4:**
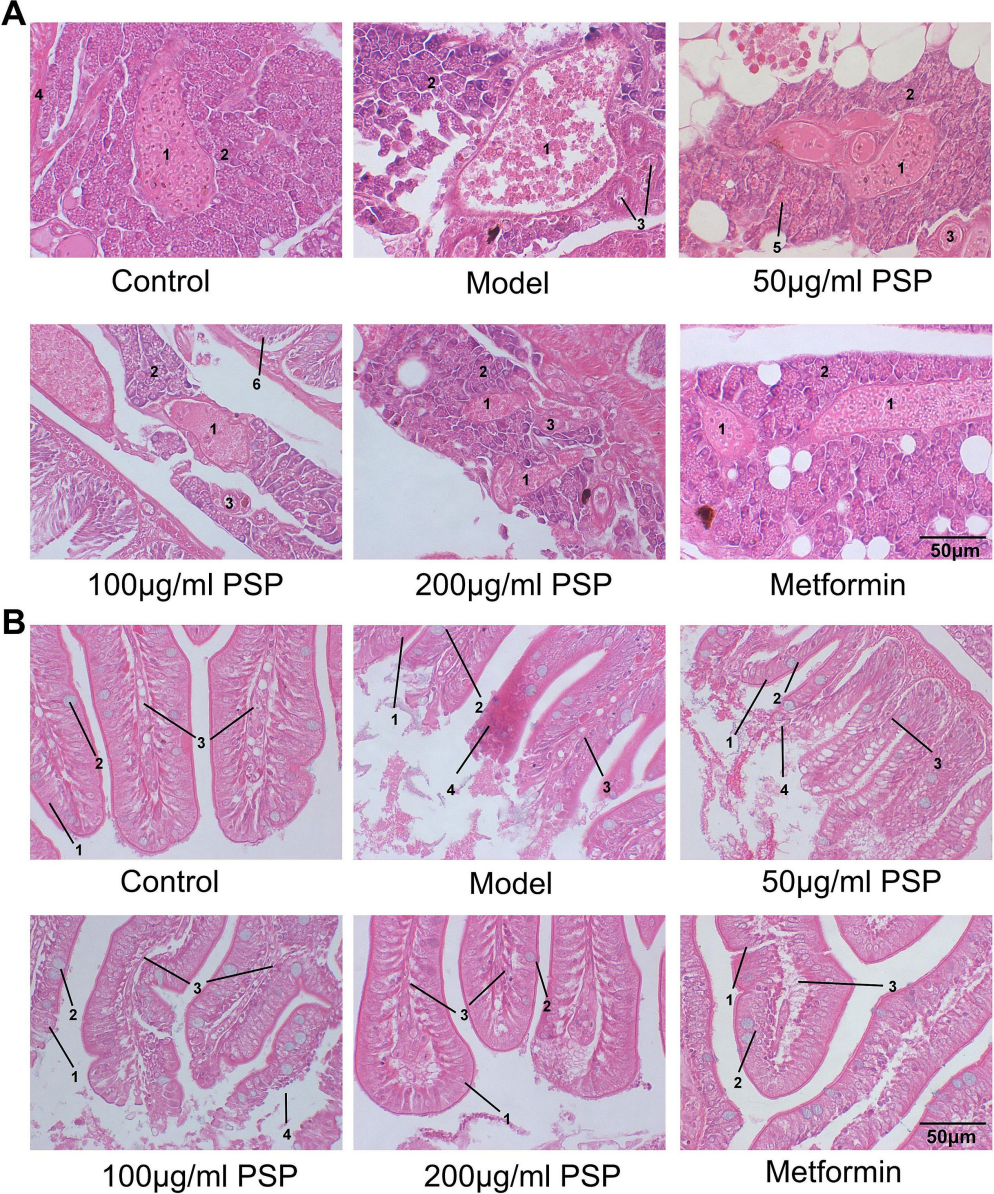
Effects of PSP on pancreatic and intestinal pathology in a zebrafish diabetic model. **(A)** Pathological morphology of zebrafish pancreatic tissue: (1) pancreatic islet; (2) exocrine pancreas (acinar cells); (3) intralobular duct; (4) blood vessel; (5) zymogen granule; (6) intestine. **(B)** Pathological morphology of zebrafish intestinal tissue: (1) epithelial cells; (2) goblet cells; (3) lamina propria; (4) inflammatory cell infiltration.

The HE staining showed distinct morphological differences between the groups. In the control group, pancreatic tissue exhibited normal architecture with intact acinar cells containing abundant zymogen granules, and well-defined pancreatic islets with clear boundaries and no signs of inflammation. In the model group, severe pancreatic damage was observed, including loss of zymogen granules in acinar cells, decreased islet volume, blurred boundaries, and a marked reduction in the number of islet cells. Treatment with PSP at 50, 100, and 200 μg/mL, as well as metformin, significantly alleviated these pathological changes. PSP treatment partially restored pancreatic architecture, including partial restoration of acinar cell granules, moderately increased islet volume, and mild islet cell proliferation. Although some acinar cells still exhibited granule loss and blurred boundaries, the overall tissue structure was notably improved (Figure 4A).

Intestinal tissue analysis revealed substantial differences between groups. In the control group, intestinal tissue showed a uniform distribution of villi, smooth boundaries, and no signs of inflammation. The villi were tall and slender, with intact columnar epithelial cells and goblet cells, and the lamina propria showed no immune cell infiltration. In the model group, the intestinal tissue exhibited severe damage, including villus loss, bifurcation, ruptured villus tips, and significant injury to the columnar epithelial cells. Extensive neutrophil and mononuclear cell infiltration indicated a marked inflammatory response. Treatment with PSP at 50, 100, and 200 μg/mL, as well as metformin, significantly improved the intestinal tissue morphology. PSP-treated groups exhibited fewer bifurcations and ruptures of the villi, clearer villus boundaries, and a marked reduction or absence of inflammatory cell infiltration, suggesting that PSP helps mitigate hyperglycemia-induced intestinal damage and inflammation (Figure 4B).

### 3.5 Network pharmacology analysis

**Figure 5:**
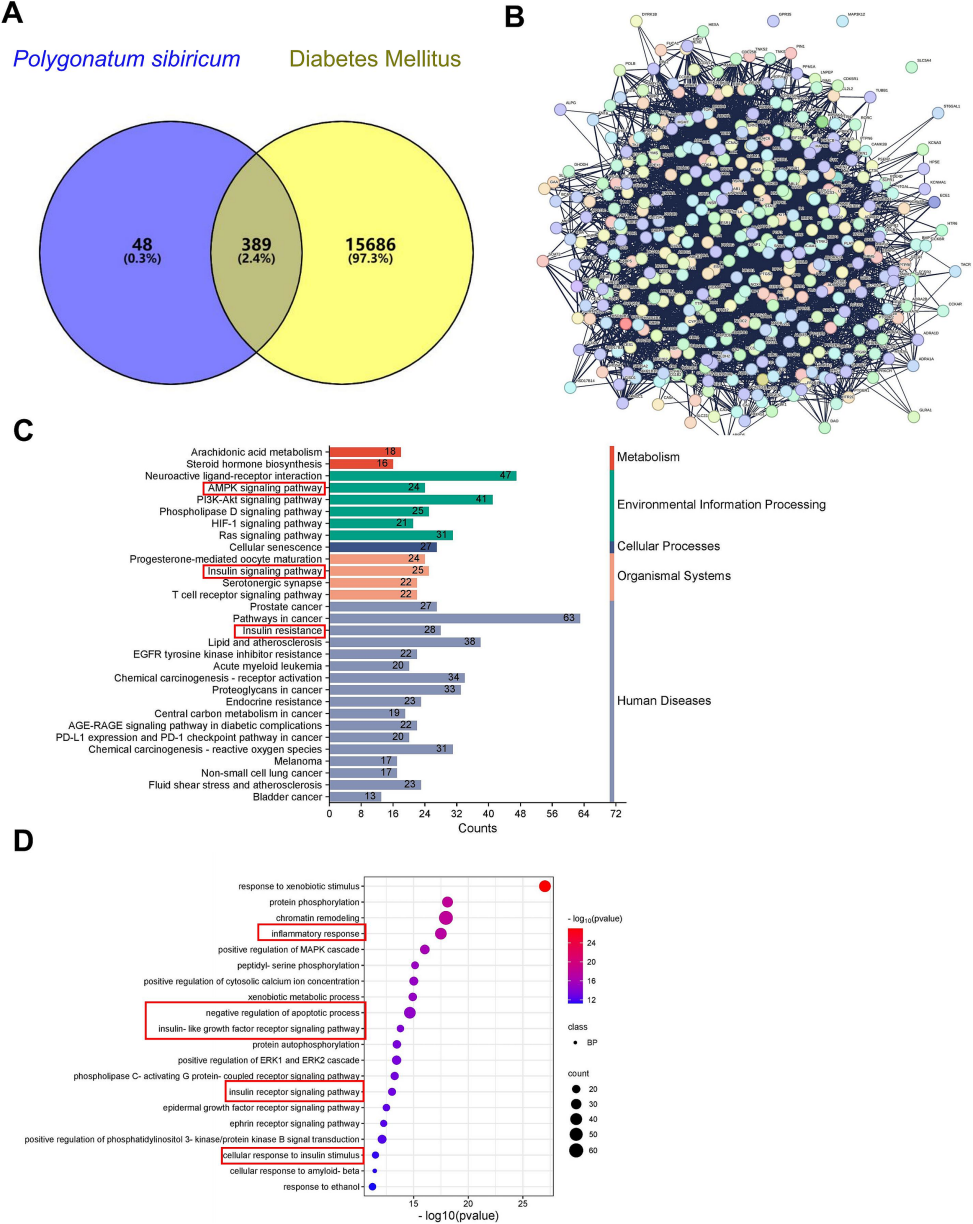
Network pharmacology analysis of PSP. **(A)** Venn diagram showing common targets between *P. sibiricum* and diabetes. **(B)** PPI network of common targets constructed using STRING database with a confidence score ≥ 0.4. **(C)** GO enrichment (BP) analysis of the common targets. **(D)** KEGG signaling pathway enrichment analysis of the common targets.

Through network pharmacology analysis, we identified a total of 442 targets associated with *P. sibiricum* and 16,075 targets related to diabetes, with 389 common targets (Figure 5A and Supplementary Tables S1–S3). These common targets underwent protein–protein interaction (PPI) analysis (Figure 5B and Supplementary Table S4), which revealed key biological processes including “AMPK signaling pathway,” “insulin resistance,” and “insulin signaling pathway” (Figure 5C and Supplementary Table S5). Key signaling pathways identified in the Kyoto Encyclopedia of Genes and Genomes (KEGG) analysis included the “inflammatory response,” “negative regulation of apoptotic process,” “insulin-like growth factor receptor signaling pathway,” “insulin receptor signaling pathway,” and “cellular response to insulin stimulus” (Figure 5D and Supplementary Table S6).

### 3.6 Molecular docking of PSP components

**Figure 6:**
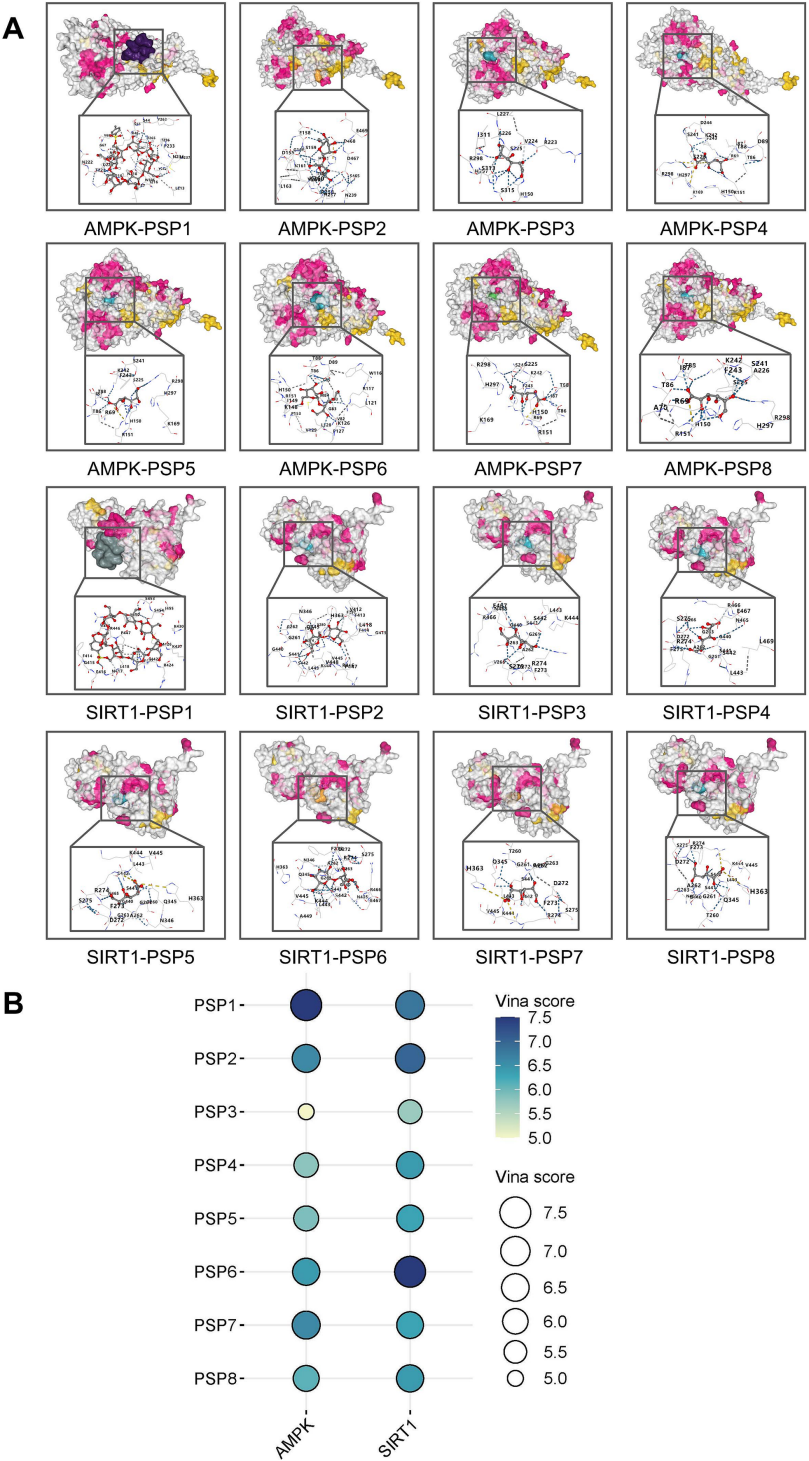
Target-active ingredient docking pattern mapping. **(A)** Mapping of target-active ingredient docking patterns. **(B)** Total score bubble map. PSP1, 6-*O*-Tosyl-β-cyclodextrin; PSP2, polysucrose; PSP3, Polyglucose; PSP4, Poly(mannuronic acid); PSP5, poly-d-galacturonic acid; PSP6, polymaltose; PSP7, galacturonic acid; PSP8, d-mannuronic acid.

Molecular docking simulations were conducted to explore potential interactions between PSP components and key targets involved in glucose metabolism, namely AMPK and SIRT1. Eight PSP-related structures were identified, including Polysucrose, 6-*O*-Tosyl-β-cyclodextrin, Polyglucose, Poly-d-galacturonic acid, Poly(mannuronic acid), Polymaltose, Galacturonic acid, and D-Mannuronic acid (Supplementary Figure S1). Docking simulations revealed that all eight PSP components exhibited strong binding affinities with AMPK and SIRT1, suggesting that they may play key roles in PSP’s hypoglycemic effects. Among these, 6-*O*-Tosyl-β-cyclodextrin, Polysucrose, Polymaltose, and Galacturonic acid displayed particularly strong binding interactions, suggesting their primary involvement in PSP’s biological activity (Figures 6A,B).

### 3.7 Effects of PSP on mRNA expression of mitochondrial damage and inflammation-related genes

**Figure 7:**
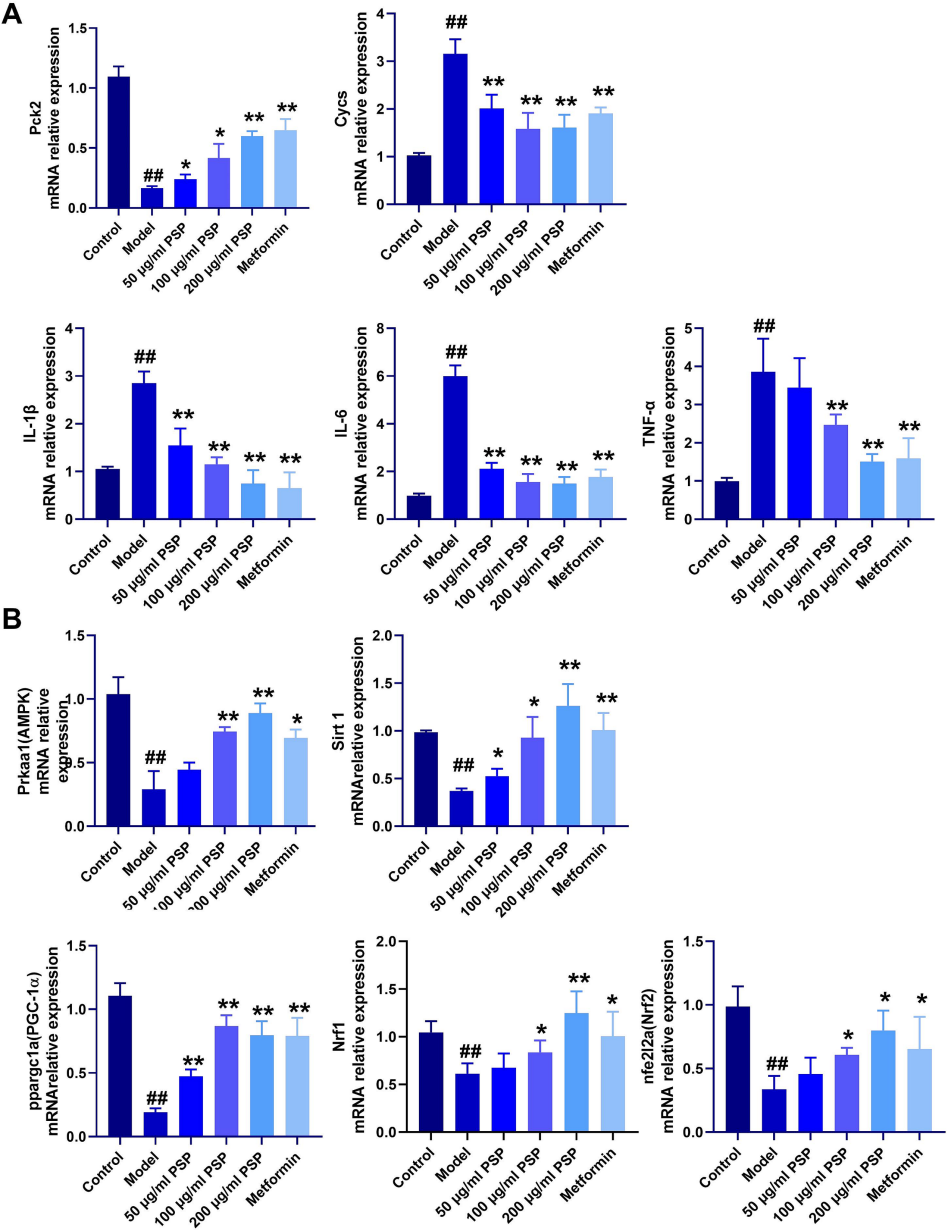
Effects of PSP on mRNA expression of relevant genes in a high-glucose-induced zebrafish model. **(A)** mRNA expression of inflammatory factors, glucose metabolism, and mitochondrial damage. **(B)** mRNA expression of genes in the AMPK-SIRT1-PGC-1α signaling pathway. ^##^*p* < 0.01 vs. Control; *p < 0.05, **p < 0.01 vs. Model; *N* = 3.

In comparison with the control group, the model group showed significantly upregulated mRNA expression of inflammatory markers (IL-1β, IL-6, TNF-*α*) and the mitochondrial damage gene Cycs (*p* < 0.01), and a significant downregulation of the glucose metabolism gene Pck2 (*p* < 0.01). Treatment with PSP at doses of 50, 100, and 200 μg/mL, as well as metformin, significantly reduced the mRNA expression of inflammatory factors and mitochondrial damage markers (*p* < 0.01) and significantly upregulated Pck2 expression (*p* < 0.01) (Figure 7A).

### 3.8 Effects of PSP on AMPK-SIRT1 pathway-related gene expression

Compared to the control group, the model group exhibited significantly downregulated mRNA expression levels of AMPK, SIRT1, PCG-1α, Nrf1, and Nrf2 (*p* < 0.01). In the 50 μg/mL PSP-treated group, the mRNA expression levels of AMPK, Nrf1, and Nrf2 remained unchanged (*p* > 0.05), while those of SIRT1 and PCG-1α were significantly upregulated (*p* < 0.05, *p* < 0.01). In the 100 μg/mL, 200 μg/mL PSP-treated groups and the metformin group, the expression of AMPK, SIRT1, PCG-1α, Nrf1, and Nrf2 were significantly increased (*p* < 0.05, *p* < 0.01) (Figure 7B).

### 3.9 Protective effects of PSP on β-cell apoptosis in zebrafish diabetes model

**Figure 8:**
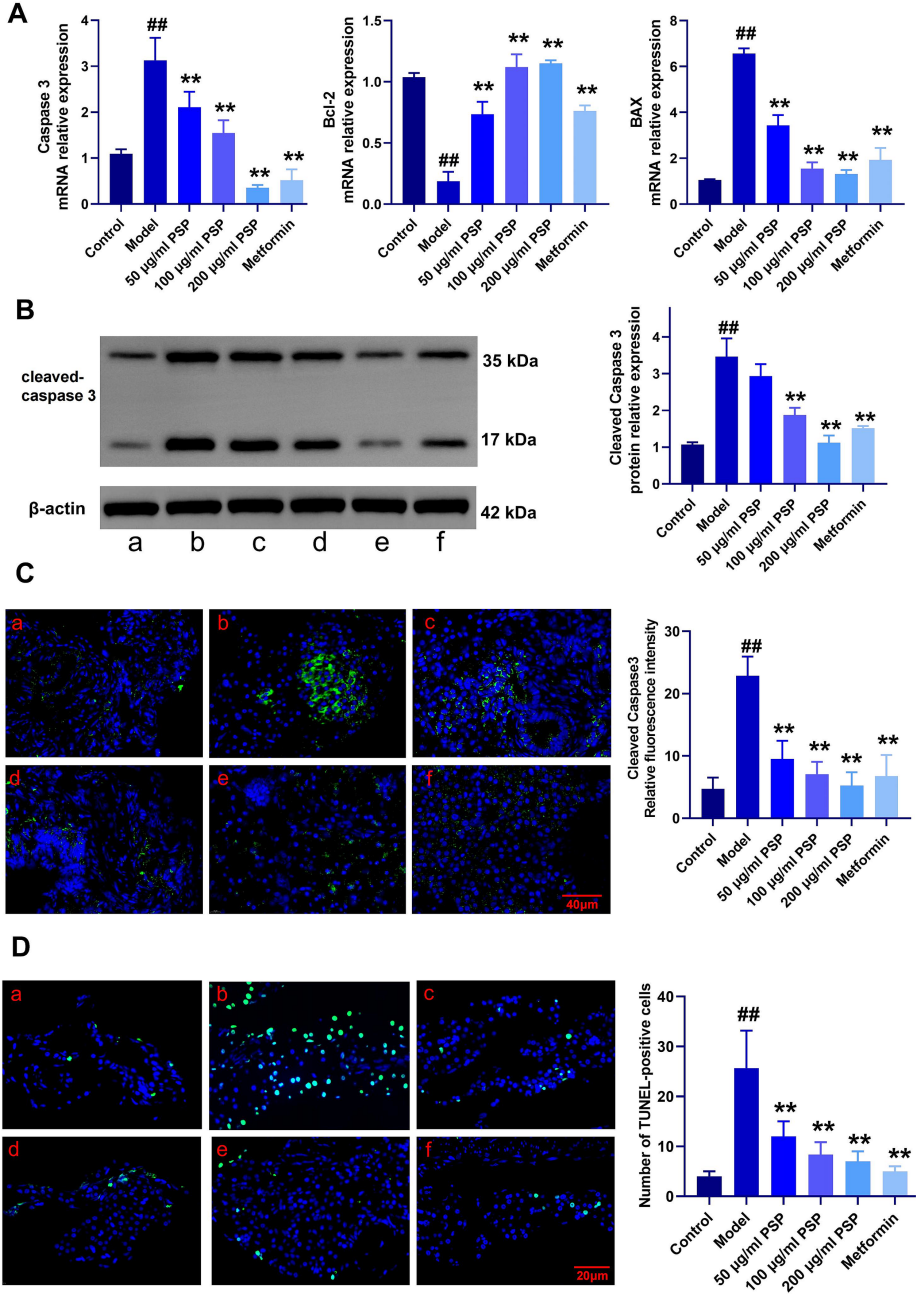
Effects of PSP on β-cell apoptosis in a zebrafish diabetes model. **(A)** mRNA expression of apoptosis-related genes. **(B)** Relative protein expression of cleaved caspase-3. **(C)** Relative fluorescence intensity of cleaved caspase-3. **(D)** Number of TUNEL-positive cells. ^##^*p* < 0.01 vs. Control; **p < 0.01 vs. Model; *N* = 3. (a) Control, (b) Model, (c) 50 μg/mL PSP, (d) 100 μg/mL PSP, (e) 200 μg/mL PSP, (f) Metformin.

Caspase 3 mRNA expression was significantly elevated in the model group compared to the control group (*p* < 0.01), indicating extensive β-cell apoptosis. PSP treatment at low, medium, and high doses (50 μg/mL, 100 μg/mL, 200 μg/mL) dose-dependently reduced Caspase 3 expression by 36, 52, and 74%, respectively (*p* < 0.01). The high-dose PSP group showed similar efficacy to the metformin-positive control, which reduced Caspase 3 expression by 68% (*p* < 0.01) (Figure 8A). Furthermore, mRNA levels of Bcl-2 were significantly decreased, while BAX expression was significantly increased in the model group. PSP treatment mitigated these changes (*p* < 0.01).

Western blot analysis confirmed that cleaved Caspase-3 protein levels were significantly higher in the model group compared to the control, but PSP and metformin treatments effectively reduced these levels (*p* < 0.01) (Figure 8B). Fluorescence intensity analysis showed a marked reduction in cleaved Caspase-3 fluorescence in the PSP-treated groups compared to the model group (*p* < 0.01) (Figure 8C). TUNEL staining further confirmed that β-cell apoptosis was significantly reduced in the PSP-treated groups (*p* < 0.01) (Figure 8D).

## 4 Discussion

Modern medical research has confirmed that the core pathology of diabetes involves impaired pancreatic β-cell function and the onset of insulin resistance, particularly the mitochondrial dysfunction and oxidative stress response in β-cells, which play a crucial role in the pathogenesis of diabetes (20). PSP, derived from *P. sibiricum*, have garnered attention in recent years for their potential role in diabetes management due to their multifaceted effects, including antioxidant, anti-inflammatory, hypoglycemic, and β-cell protective properties (21, 22). This study evaluates the hypoglycemic effects of PSP in a zebrafish diabetes model, particularly focusing on its protective effects on β-cells, improvement of mitochondrial function, and regulation of the AMPK-SIRT1 signaling pathway.

Chronic hyperglycemia is one of the major characteristics of diabetes, and its pathological mechanisms are closely linked to insulin resistance and impaired pancreatic β-cell function. Insulin resistance primarily occurs in insulin target organs such as skeletal muscle, adipose tissue, and the liver, leading to reduced glucose uptake, increased gluconeogenesis, and elevated blood glucose levels (23). This study demonstrates that PSP significantly lowers blood glucose and fructosamine levels in a zebrafish diabetes model, showcasing a pronounced hypoglycemic effect. PSP exerts dual regulatory effects on glucose metabolism, addressing both peripheral insulin resistance and hepatic gluconeogenic dysregulation. Specifically, PSP enhances insulin-dependent glucose uptake by upregulating glucose transporter 4 (GLUT4) expression in skeletal muscle and adipose tissue, which is consistent with previous reports indicating PSP’s ability to improve insulin sensitivity and glycemic control in diabetic models (24). Concurrently, PSP suppresses hepatic glucose overproduction by transcriptionally downregulating gluconeogenesis-related genes, particularly Pck2 (phosphoenolpyruvate carboxykinase), thereby mitigating hyperglycemia. These findings align with those of a recent study using a db/db mouse model, where PSP inhibited gluconeogenesis via suppression of Pck2 expression and enhanced hepatic glycogen storage, restoring glucose homeostasis (25).

Oxidative stress plays a pivotal role in the progression of diabetes. Under hyperglycemic conditions, pancreatic β-cells, due to increased mitochondrial activity, produce an excess of reactive oxygen species (ROS). These ROS not only damage proteins, lipids, and DNA but also activate pro-apoptotic pathways, leading to β-cell apoptosis. Given that pancreatic β-cells have relatively low antioxidant capacity, they are particularly vulnerable to oxidative damage, which further accelerates the progression of diabetes (26). This study shows that PSP significantly alleviates oxidative damage in β-cells by enhancing antioxidant enzyme activities such as superoxide dismutase (SOD) and catalase (CAT), and by reducing lipid peroxidation products like malondialdehyde (MDA). These findings indicate that PSP mitigates oxidative stress-induced damage in β-cells under hyperglycemic conditions by inhibiting ROS production and promoting antioxidant enzyme expression. This is in agreement with the findings of Wang et al. (27), who observed reduced MDA levels and increased SOD/CAT activity in diabetic models, supporting our observation of oxidative stress reduction.

The dysfunction and loss of pancreatic β-cells are central pathological events in the progression of type 2 diabetes. Under hyperglycemic conditions, mitochondrial dysfunction in β-cells results in the excessive production of ROS, activating pro-apoptotic pathways and ultimately leading to cell apoptosis (28). The loss of β-cells further diminishes insulin secretion, thus accelerating the development of diabetes (29). Our findings indicate that PSP mitigates hyperglycemia-induced mitochondrial oxidative stress in pancreatic β-cells through coordinated molecular mechanisms. PSP prevents mitochondrial damage by downregulating critical stress-responsive genes such as Cycs (cytochrome c), while also attenuating apoptosis by upregulating anti-apoptotic proteins (Bcl-2) and inhibiting pro-apoptotic effectors (Bax and Caspase-3). These results corroborate prior studies that highlighted PSP’s ability to preserve mitochondrial integrity under pathological conditions. For instance, research on age-related muscle dysfunction models revealed that PSP stabilizes calcium homeostasis and suppresses mitochondrial-associated membrane (MAM) formation, thereby reducing oxidative stress and cellular damage (30). Similarly, in diabetic systems, PSP has been shown to inhibit mitochondrial apoptotic pathways, further confirming its role in safeguarding β-cell viability under glucotoxic stress (31).

Network pharmacology and molecular docking results highlight a robust mechanistic framework for PSP’s effects on diabetes (32). By integrating data from various databases, we identified 389 common targets between PSP and diabetes-related pathways. These targets were notably enriched in pathways related to inflammation, insulin resistance, and mitochondrial dysfunction—core components of diabetes pathophysiology. The AMPK-SIRT1 signaling pathway emerged as a key regulator in this network. This is particularly significant as AMPK activation promotes mitochondrial biogenesis and enhances cellular stress responses, while SIRT1 regulates the deacetylation of critical proteins involved in mitochondrial function and oxidative stress management (33). Molecular docking studies further validated the interaction between PSP components and the AMPK-SIRT1 pathway. Docking simulations confirmed that PSP’s active components exhibit strong binding affinities with both AMPK and SIRT1, suggesting that PSP may directly interact with these targets to activate downstream signaling events critical for mitochondrial function and β-cell survival. Although these results suggest a direct interaction between PSP components and AMPK/SIRT1, it is possible that PSP also interacts with other receptors or pathways. Further studies are needed to explore these potential interactions.

The AMPK-SIRT1 signaling pathway is crucial for regulating cellular energy metabolism, oxidative stress defense, and cell survival. AMPK, a cellular energy sensor, is activated under energy-deficient conditions and promotes glucose uptake, fatty acid oxidation, and mitochondrial biogenesis, while inhibiting gluconeogenesis, thereby maintaining energy balance (34). SIRT1, an NAD^+^-dependent deacetylase, complements AMPK by enhancing mitochondrial function and improving metabolic disorders associated with diabetes. Through deacetylation, SIRT1 activates key regulators such as PGC-1α, Nrf1, and Nrf2, which are essential for mitochondrial biogenesis, antioxidant defense, and cellular energy utilization (35, 36). A critical aspect of the AMPK-SIRT1 pathway is its role in regulating cell apoptosis. AMPK activation reduces apoptosis by alleviating oxidative stress and enhancing mitochondrial function, while SIRT1 promotes cell survival by deacetylating pro-apoptotic proteins and boosting antioxidant defenses (37, 38). Similarly, SIRT1-mediated deacetylation of PGC-1α enhances mitochondrial biogenesis and function, thereby reducing oxidative damage and apoptosis in pancreatic β-cells (39). In this study, we found that PSP significantly activates the AMPK-SIRT1 signaling pathway, leading to the upregulation of downstream regulatory factors like PGC-1α, Nrf1, and Nrf2. These factors play key roles in enhancing mitochondrial function, boosting antioxidant defenses, and protecting cells from oxidative stress-induced damage. Specifically, PGC-1α, a major regulator of mitochondrial biogenesis, increases mitochondrial quantity and function, improving cellular energy utilization. Nrf1 and Nrf2, on the other hand, regulate the expression of antioxidant genes, further enhancing cellular resistance to oxidative stress (40–42). The activation of the AMPK-SIRT1 pathway by PSP not only improves mitochondrial function in pancreatic β-cells but also enhances their resistance to oxidative stress and apoptosis. This is evidenced by the reduced expression of cleaved caspase-3 and Bax, along with increased expression of Bcl-2 in PSP-treated groups. These findings suggest that PSP exerts its protective effects on β-cells by mitigating mitochondrial oxidative damage and apoptosis, which are central to the pathogenesis of diabetes.

Zebrafish, due to their high genetic similarity to humans, particularly in terms of pancreatic structure and glucose homeostasis regulation, are considered an ideal animal model for studying type 2 diabetes (43). Among the existing methods for constructing zebrafish diabetes models, the glucose immersion method is widely used because of its simplicity, accessibility to experimental materials, low mortality rate, and high model stability. This method involves exposing zebrafish to high-concentration glucose solutions, creating a hyperglycemic environment that places stress on the pancreas, leading to insulin resistance and glucose metabolism disorders (13). In this study, zebrafish in the model group exhibited significant behavioral changes, including increased swimming speed, heightened sensitivity to external stimuli, elevated blood glucose levels, and the excretion of white, fibrous metabolic products. These manifestations are similar to clinical symptoms observed in type 2 diabetes patients, such as metabolic disorders and hyperglycemia. This study further examined the intervention effects of PSP in this model. The results showed that PSP significantly improved zebrafish blood glucose levels, suggesting its potential to maintain glucose homeostasis. Since blood glucose levels are a key clinical indicator in type 2 diabetes, their regulation reflects the efficacy of therapeutic interventions. The zebrafish diabetes model used in this study not only demonstrated the regulatory effects of PSP on blood glucose but also provides a valuable reference for future drug screening and mechanistic research.

This study has several limitations. First, although zebrafish provide valuable insights into diabetic mechanisms, further validation in mammalian models is necessary. Second, the exact bioactive components within PSP responsible for AMPK-SIRT1 modulation require further isolation and characterization. Lastly, pharmacokinetic and toxicity studies in higher-order models are essential to support clinical translation.

## 5 Conclusion

In summary, PSP demonstrates promising potential as a natural multi-target therapeutic agent for diabetes, primarily by enhancing mitochondrial function and reducing β-cell apoptosis via activation of the AMPK-SIRT1 axis. These findings suggest that PSP may serve as an adjunct or alternative to current diabetes treatments. Future research should focus on validating these findings in mammalian models and clinical settings, and elucidating the bioavailability, pharmacokinetics, and possible combinational therapies involving PSP.

## Data Availability

The original contributions presented in the study are included in the article/Supplementary material, further inquiries can be directed to the corresponding authors.

## Footnotes

^1^ https://old.tcmsp-e.com/

^2^ http://www.swisstargetprediction.ch/

^3^ https://www.genecards.org/

^4^ https://bioinfogp.cnb.csic.es/tools/venny/

^5^ https://cn.string-db.org/

^6^ https://davidbioinformatics.nih.gov/

^7^ https://scifinder-n.cas.org/

^8^ https://www.rcsb.org/

^9^ https://cadd.labshare.cn/cb-dock2/index.php

^10^ http://bioinformatics.com.cn/
