# Supplementary figures and images for: Polygonatum sibiricum polysaccharides enhance pancreatic β-cell function in diabetic zebrafish by mitigating mitochondrial oxidative damage via the AMPK-SIRT1 pathway
Source: Front Nutr. 2025 May 9;12:1601490. doi: 10.3389/fnut.2025.1601490 (PMC12128605; doi:10.3389/fnut.2025.1601490)

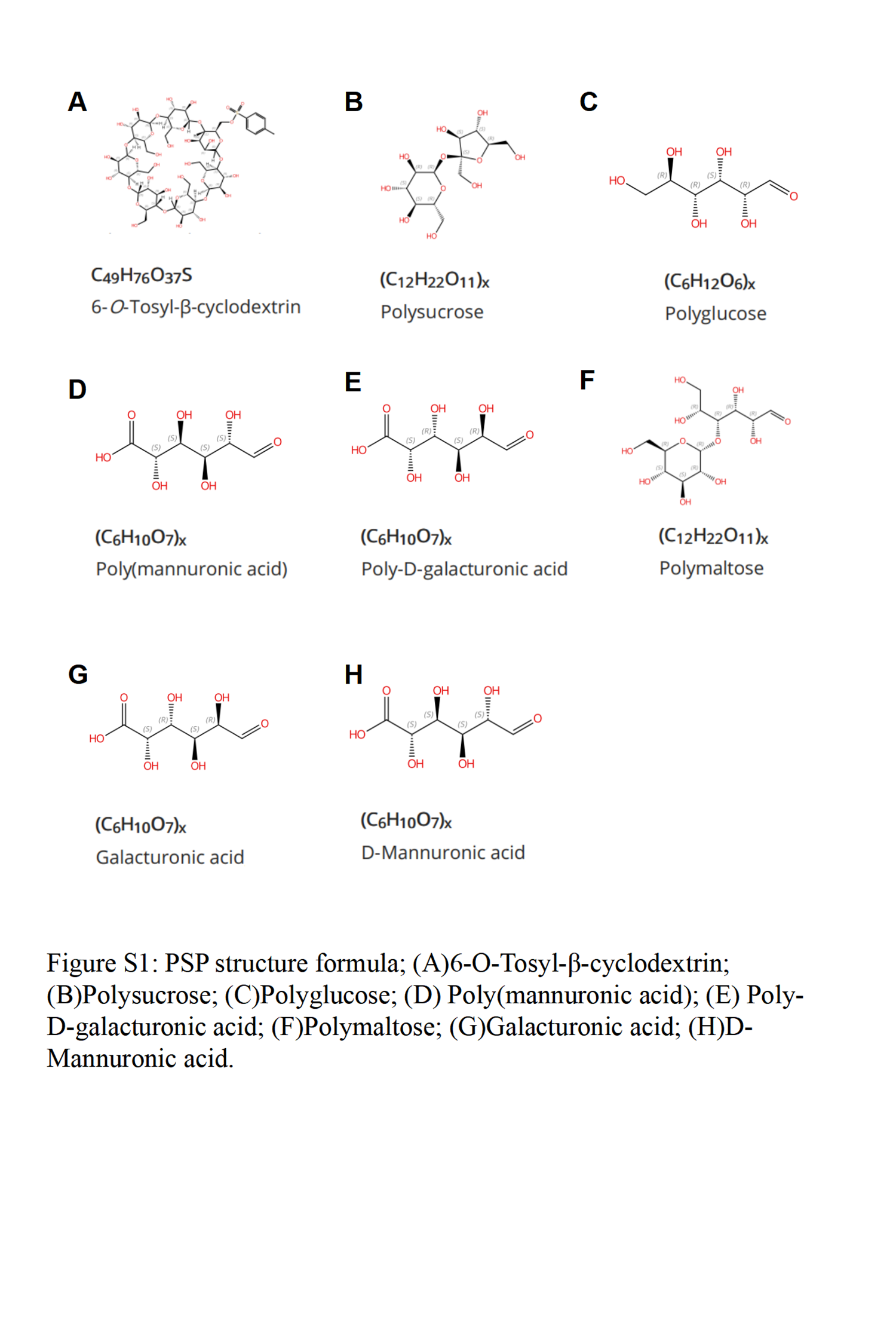

Supplement: Supplementary file 1 [file Image_1.TIF]

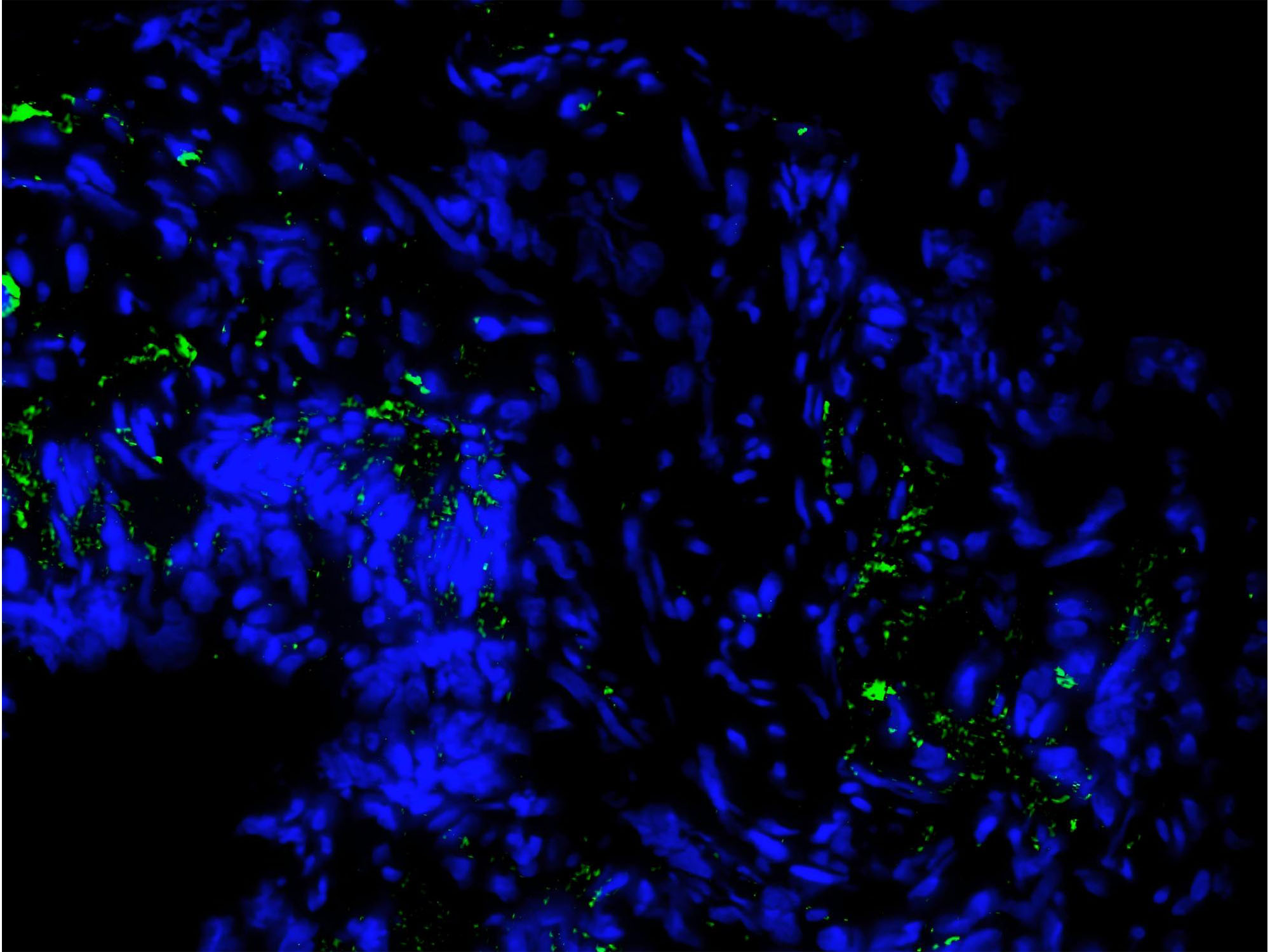

Supplement: Supplementary file 11 [file Data_Sheet_3.ZIP › IF-c-caspase/100 PSP-1.jpg]

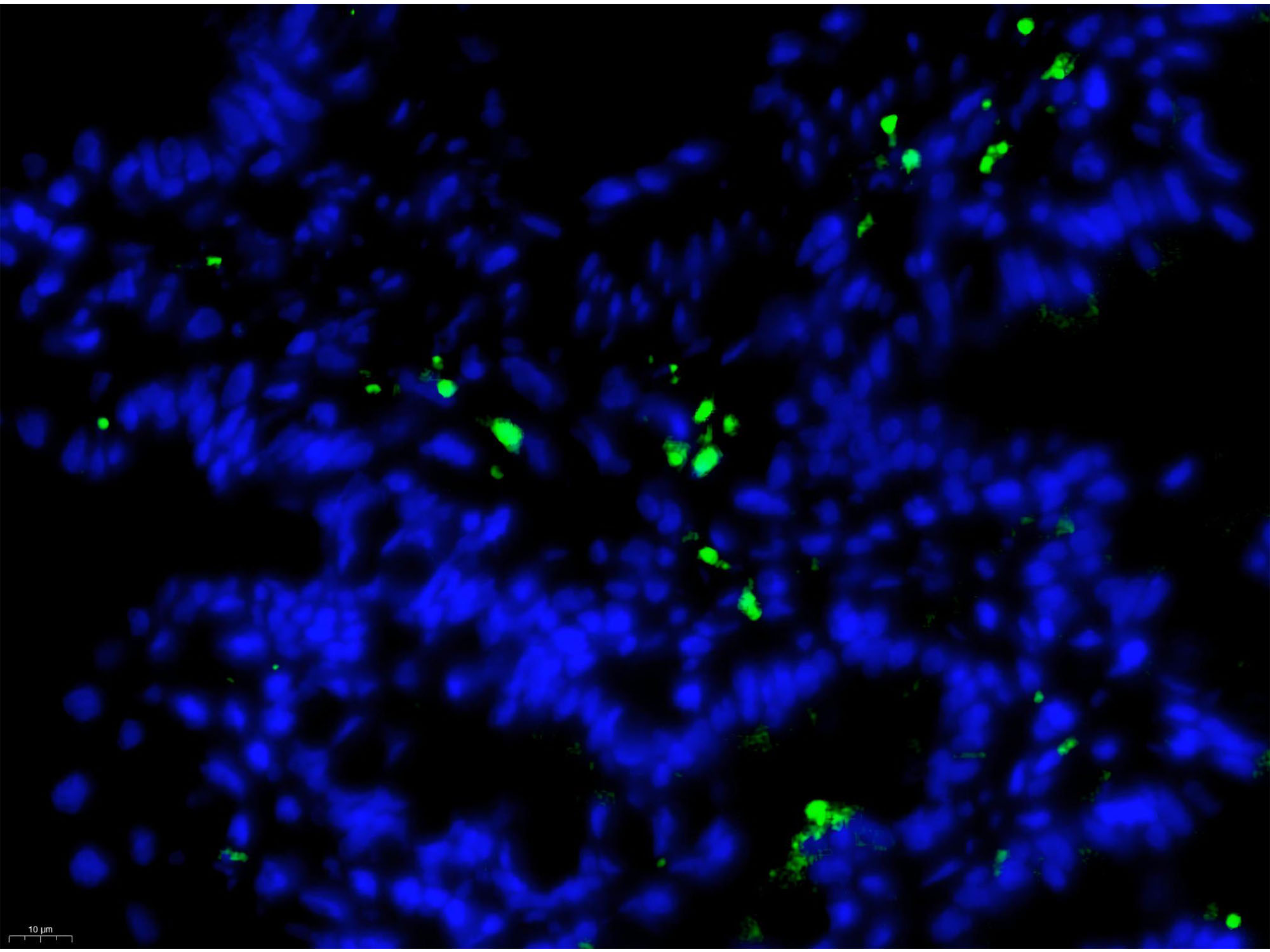

Supplement: Supplementary file 11 [file Data_Sheet_3.ZIP › IF-c-caspase/100 PSP-2.jpg]

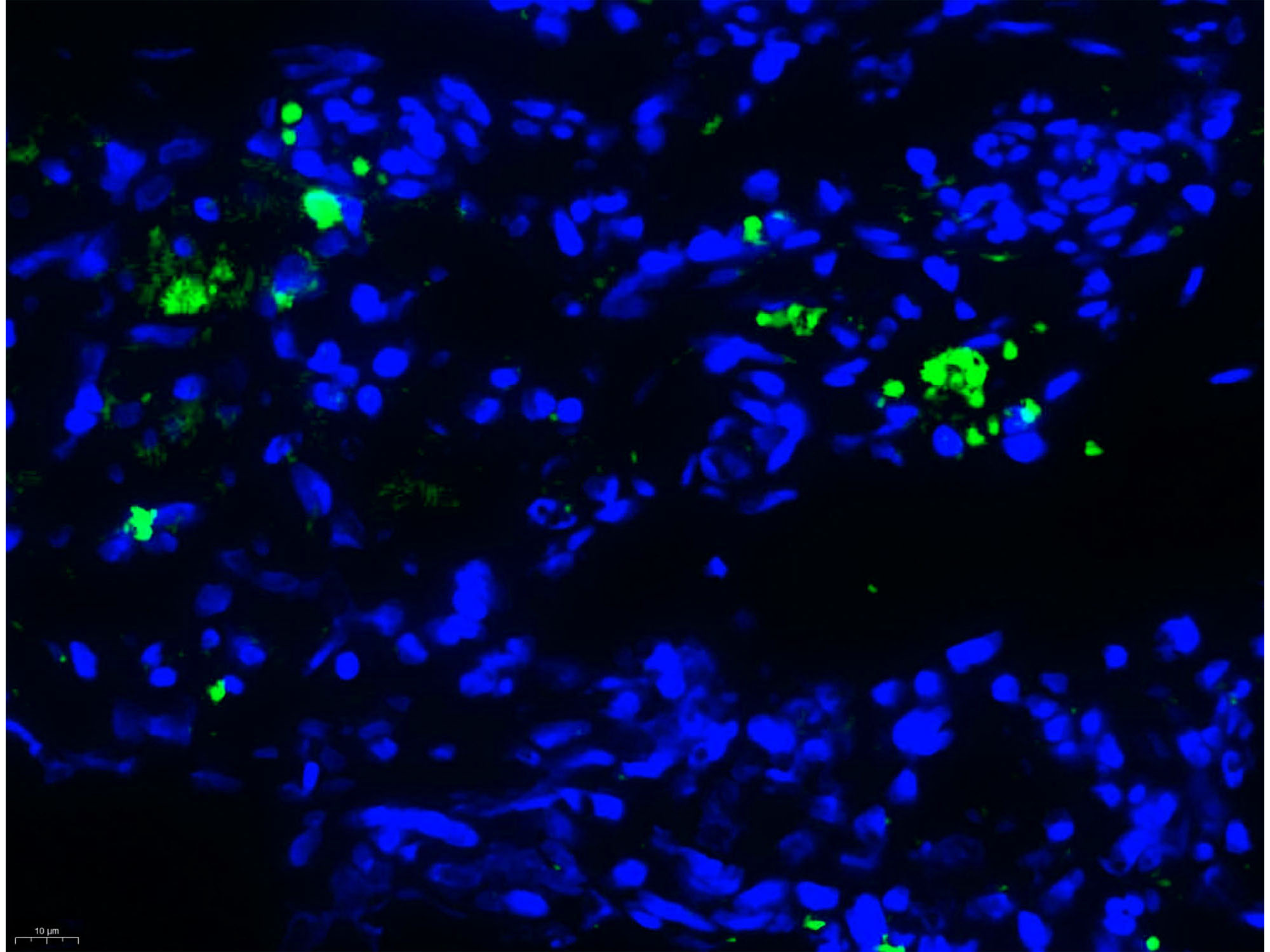

Supplement: Supplementary file 11 [file Data_Sheet_3.ZIP › IF-c-caspase/100 PSP-3.jpg]

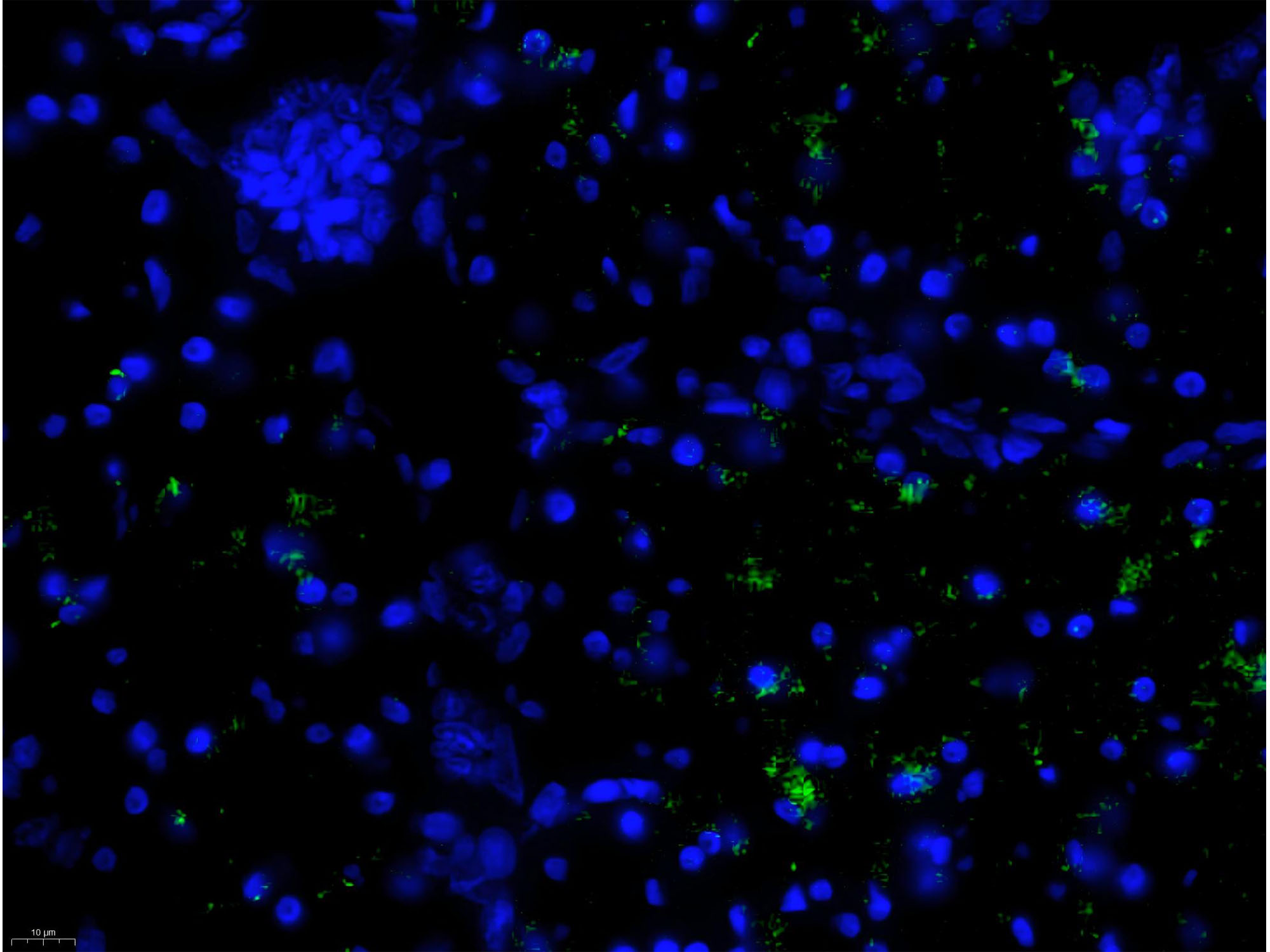

Supplement: Supplementary file 11 [file Data_Sheet_3.ZIP › IF-c-caspase/200 PSP-1.jpg]

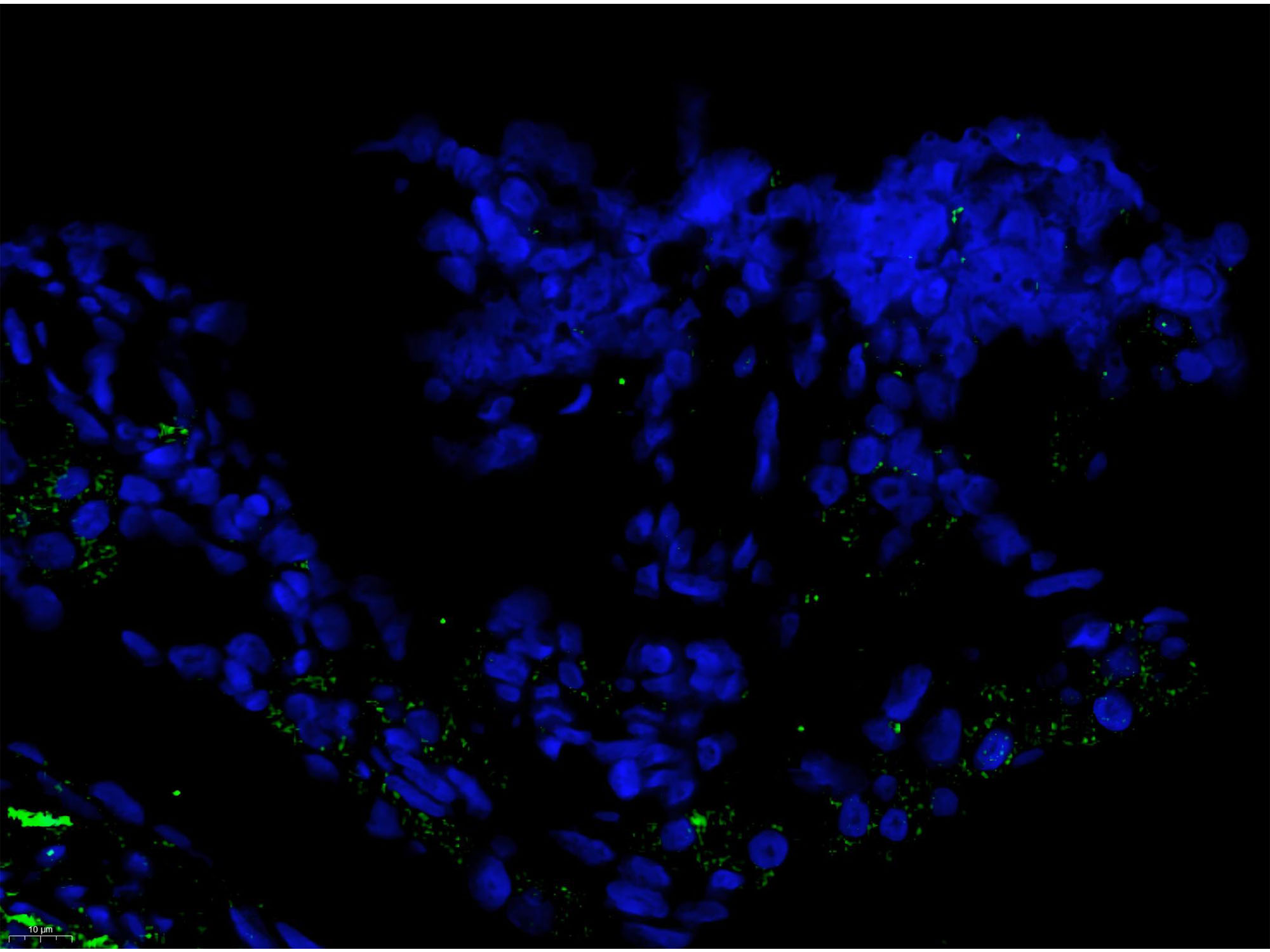

Supplement: Supplementary file 11 [file Data_Sheet_3.ZIP › IF-c-caspase/200 PSP-2.jpg]

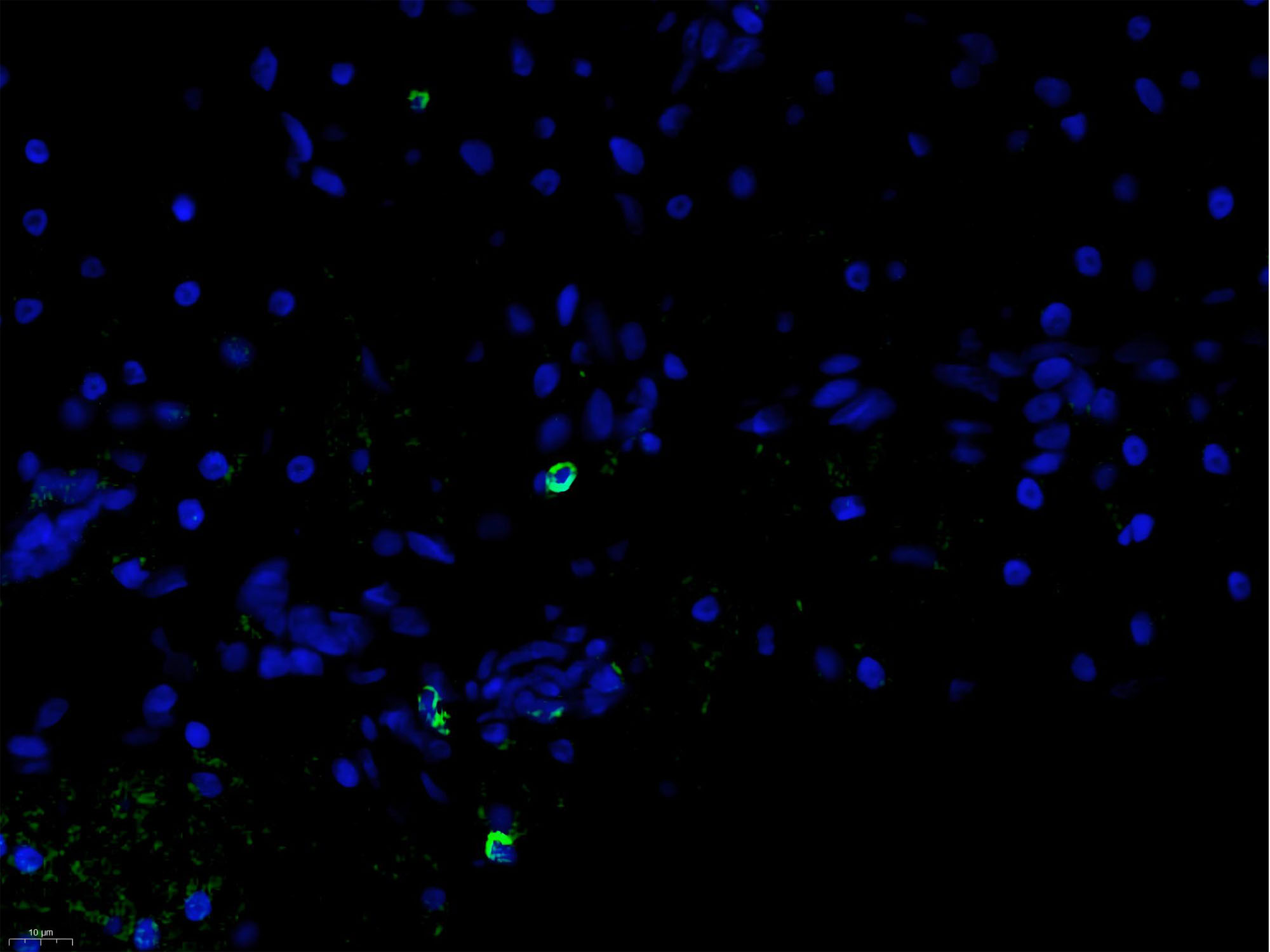

Supplement: Supplementary file 11 [file Data_Sheet_3.ZIP › IF-c-caspase/200 PSP-3.jpg]

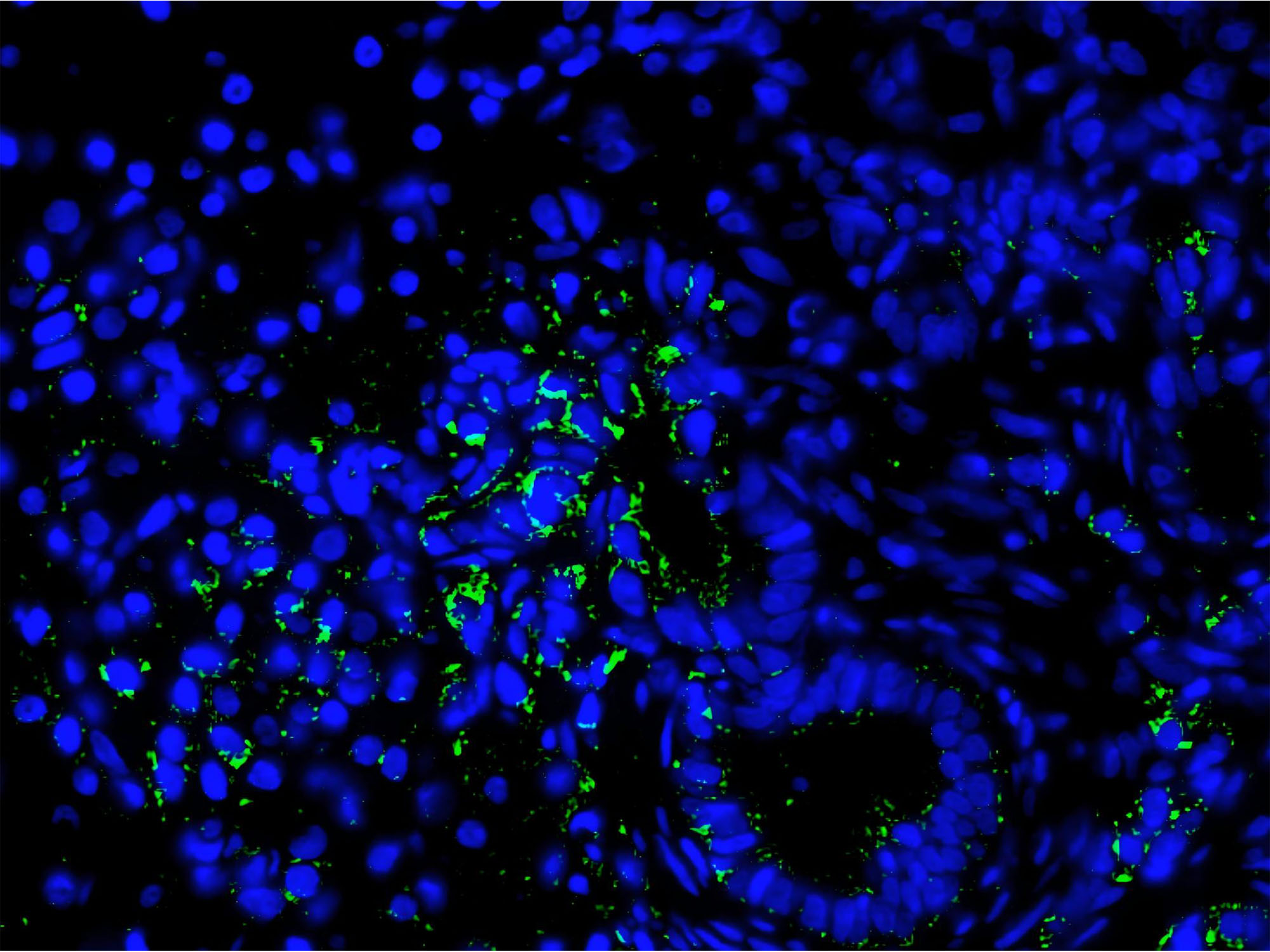

Supplement: Supplementary file 11 [file Data_Sheet_3.ZIP › IF-c-caspase/50 PSP-1.jpg]

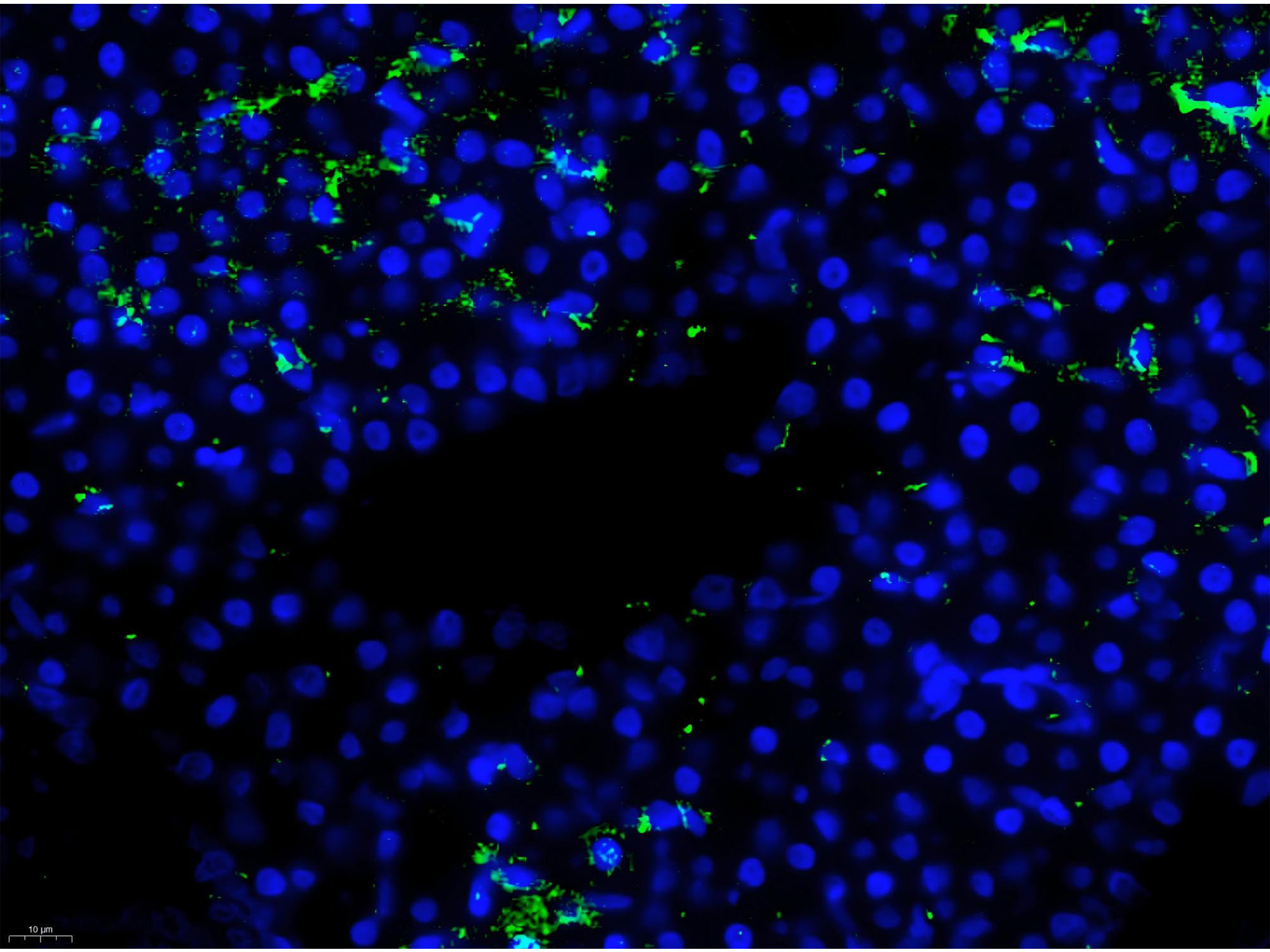

Supplement: Supplementary file 11 [file Data_Sheet_3.ZIP › IF-c-caspase/50 PSP-2.jpg]

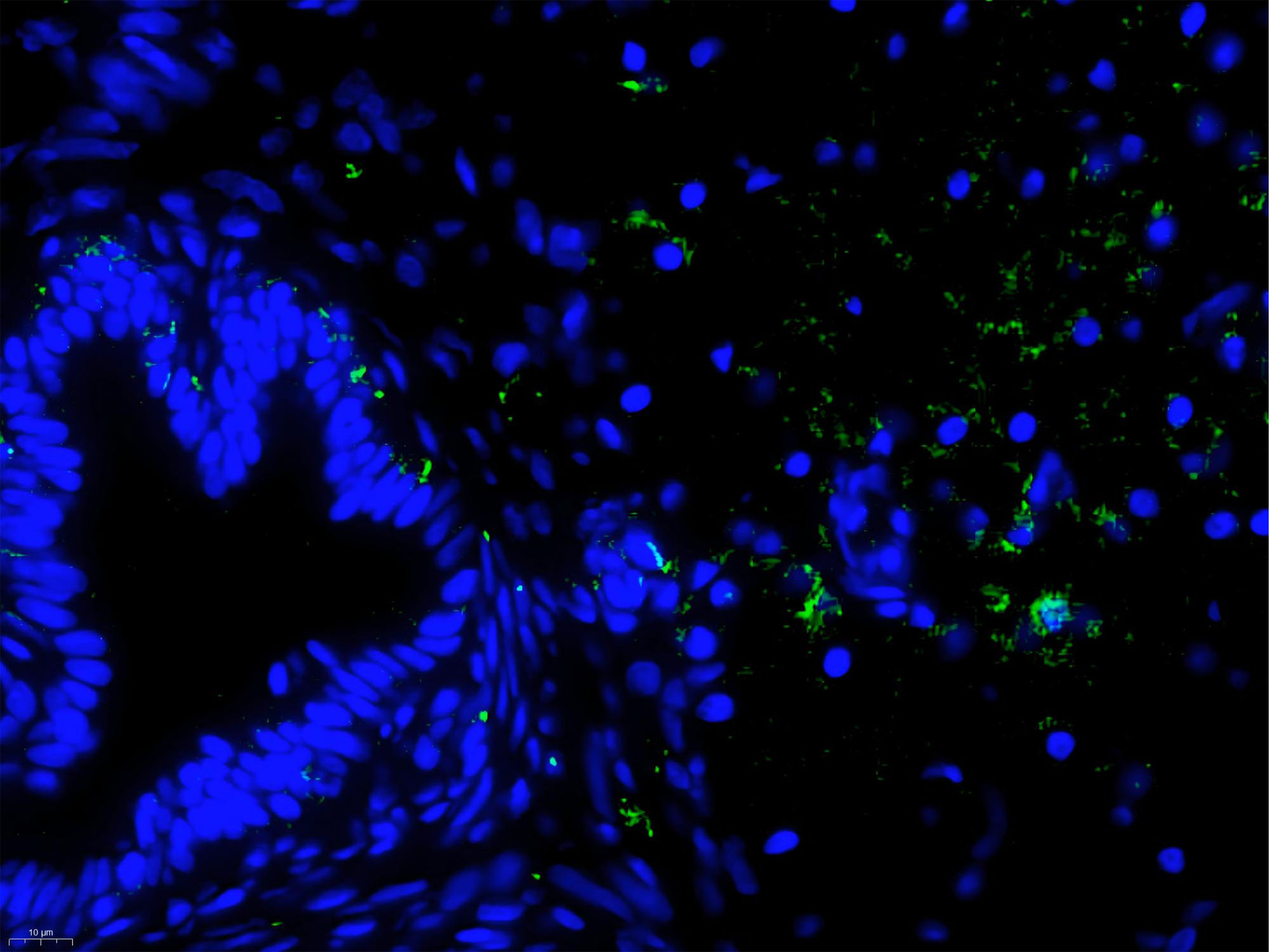

Supplement: Supplementary file 11 [file Data_Sheet_3.ZIP › IF-c-caspase/50 PSP-3.jpg]

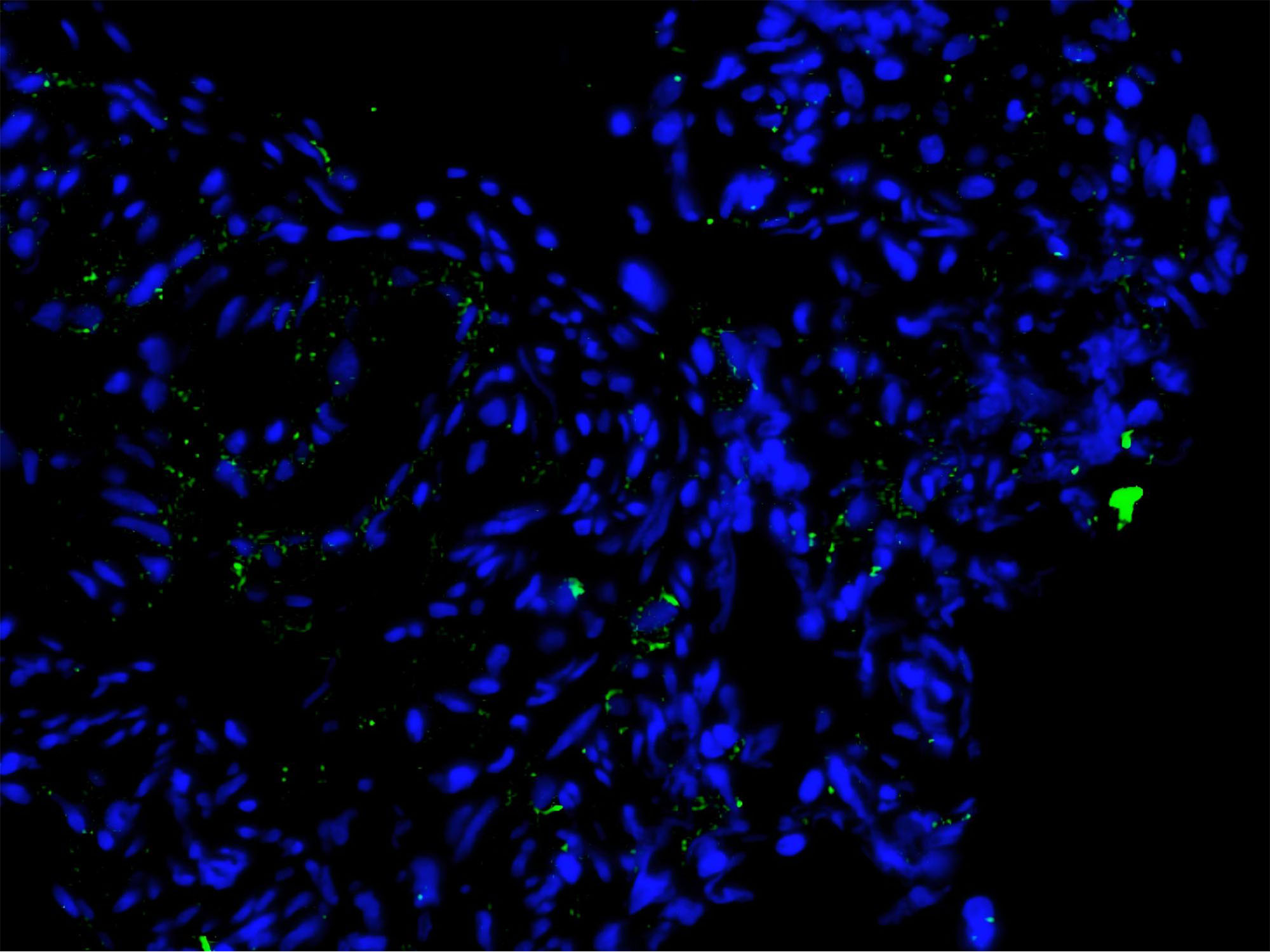

Supplement: Supplementary file 11 [file Data_Sheet_3.ZIP › IF-c-caspase/Control-1.jpg]

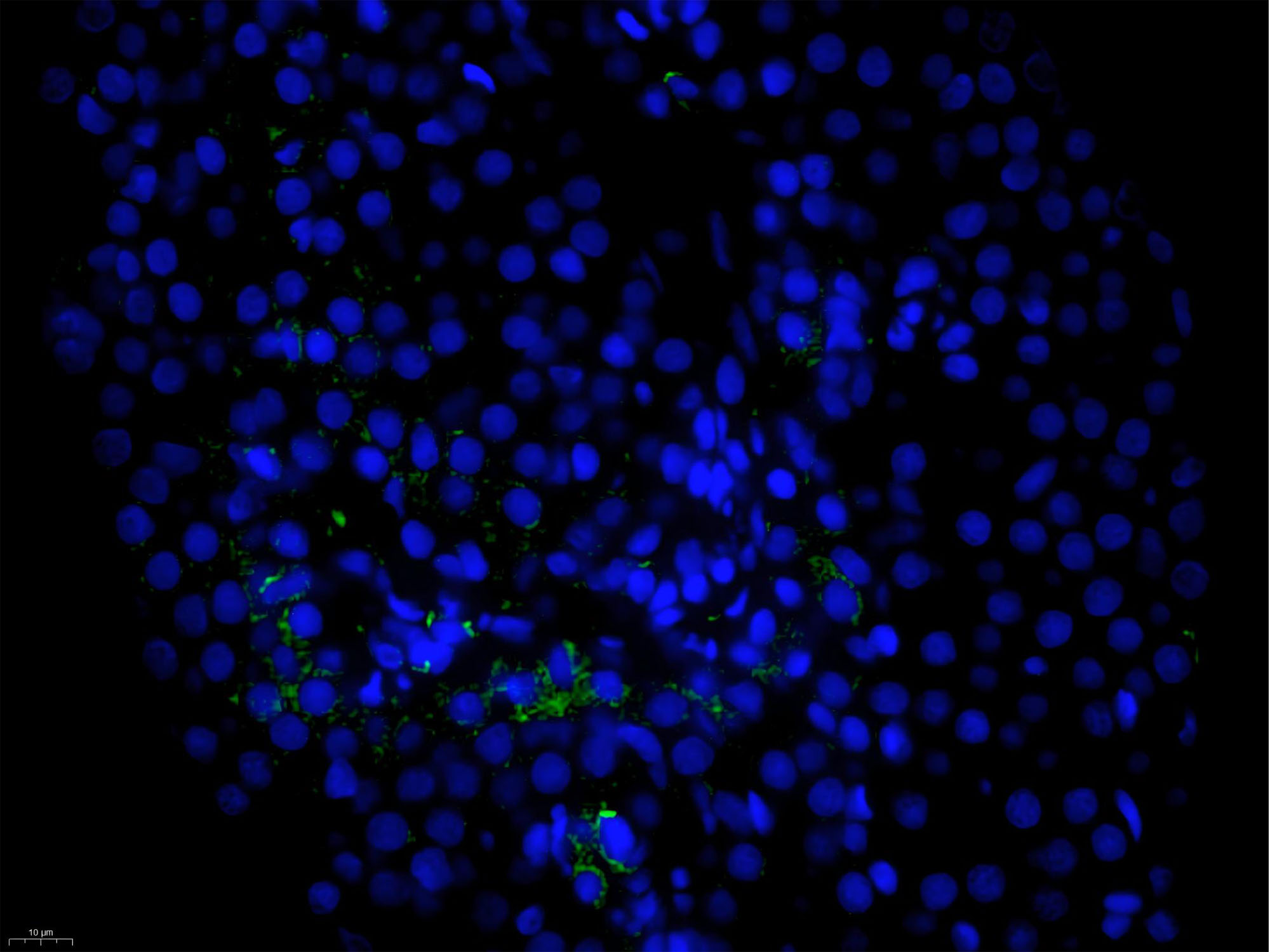

Supplement: Supplementary file 11 [file Data_Sheet_3.ZIP › IF-c-caspase/Control-3.jpg]

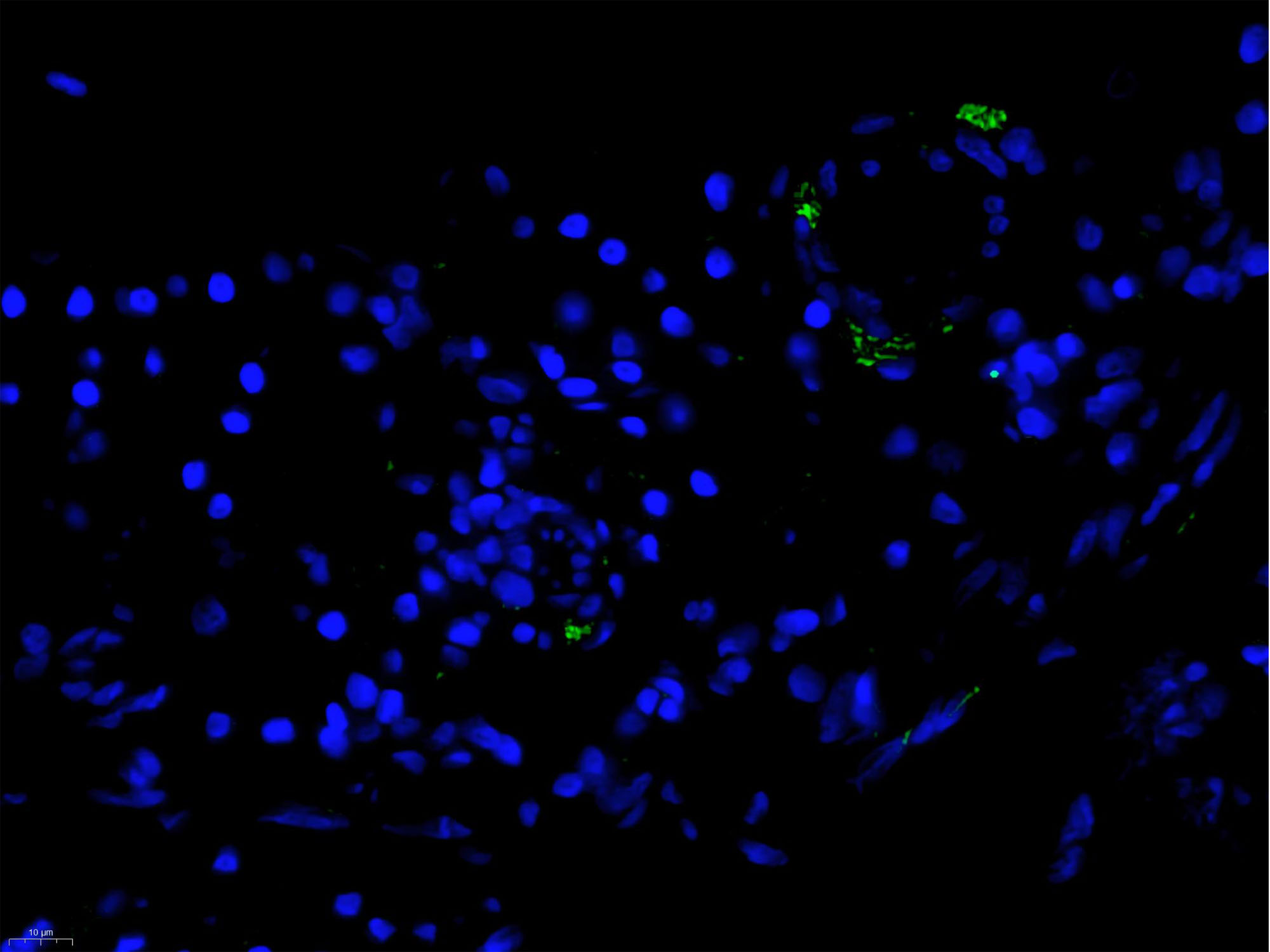

Supplement: Supplementary file 11 [file Data_Sheet_3.ZIP › IF-c-caspase/Control2.jpg]

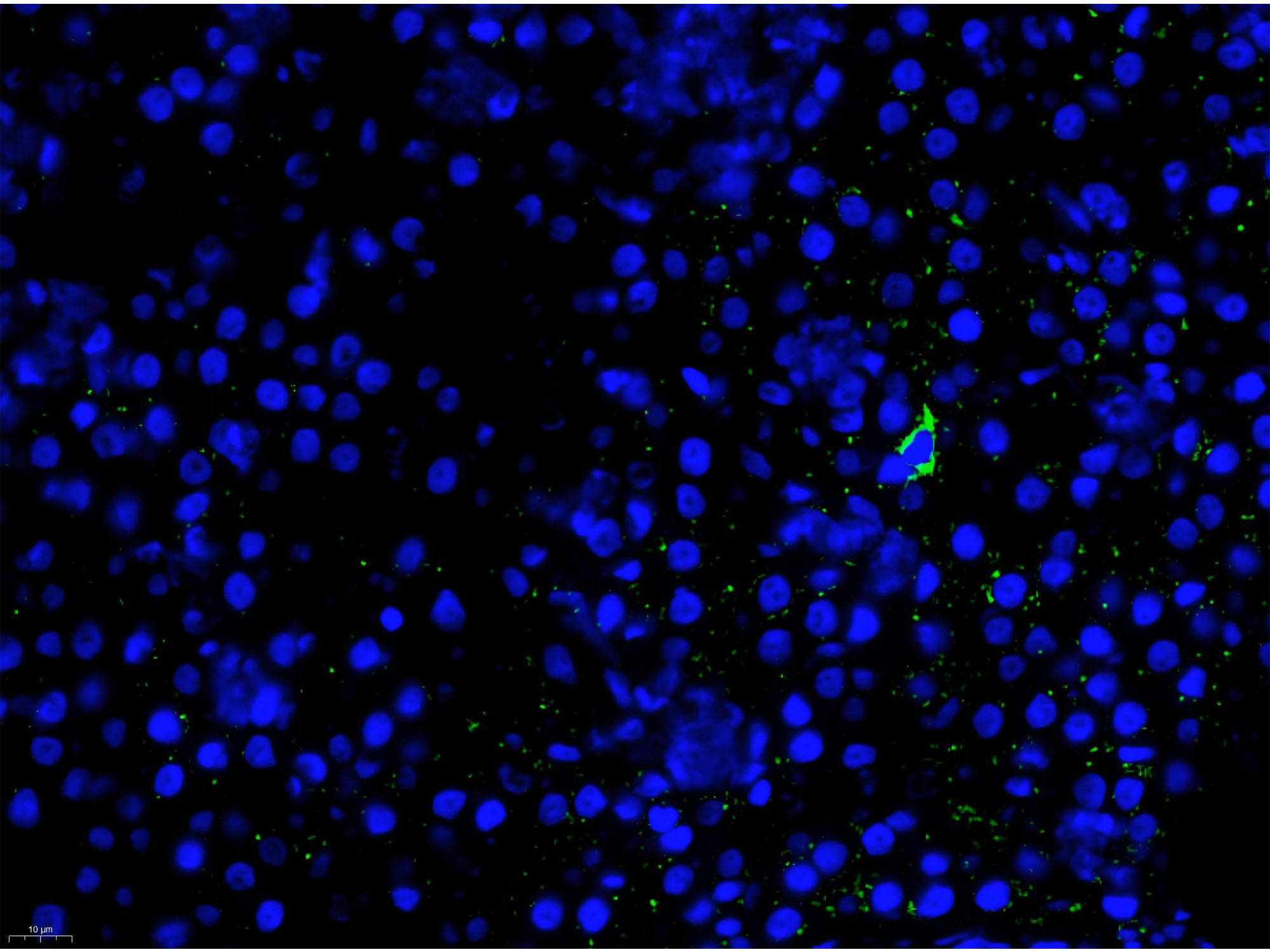

Supplement: Supplementary file 11 [file Data_Sheet_3.ZIP › IF-c-caspase/Metformin-2.jpg]

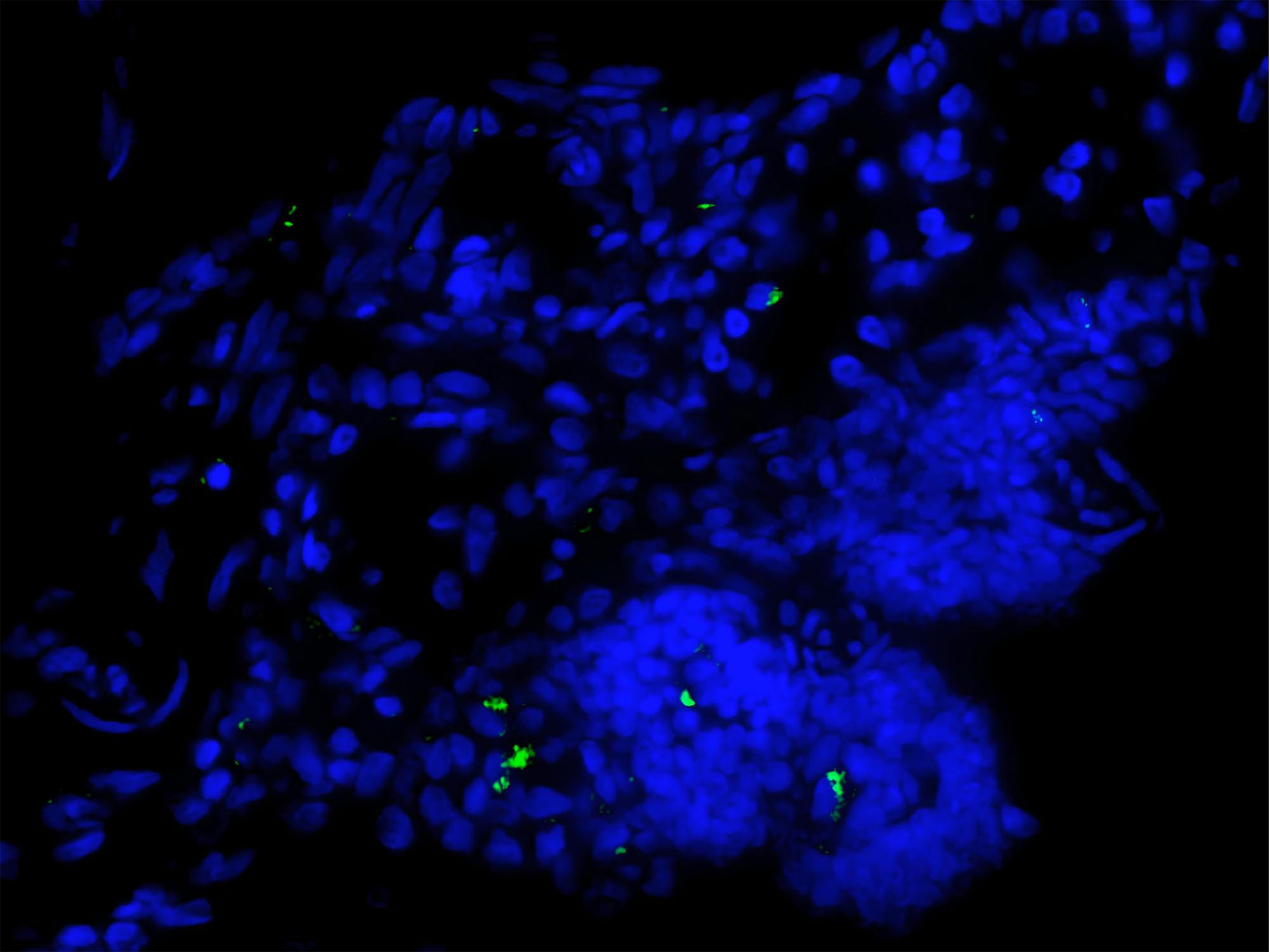

Supplement: Supplementary file 11 [file Data_Sheet_3.ZIP › IF-c-caspase/Metformin-3.jpg]

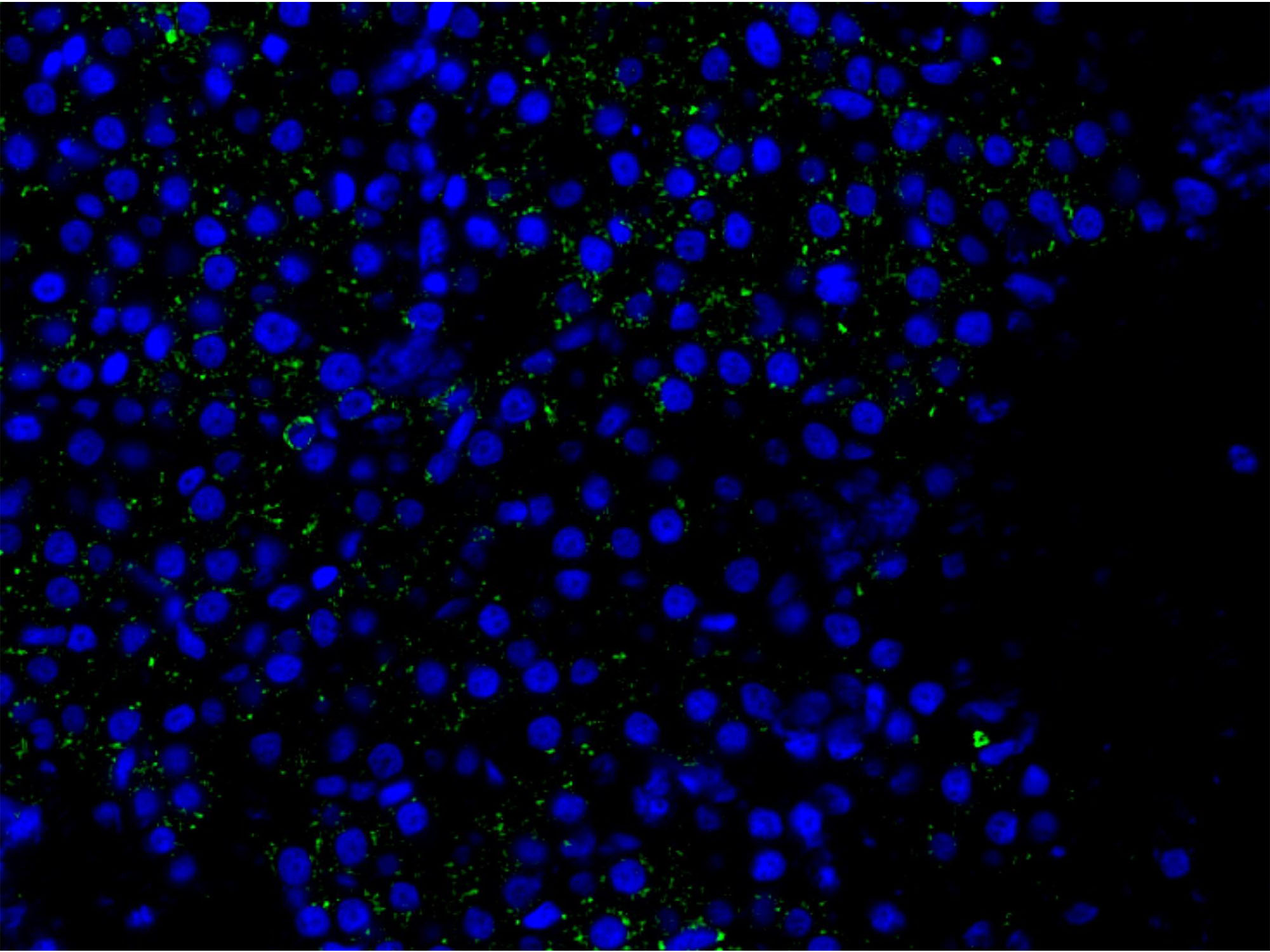

Supplement: Supplementary file 11 [file Data_Sheet_3.ZIP › IF-c-caspase/Metformin1.jpg]

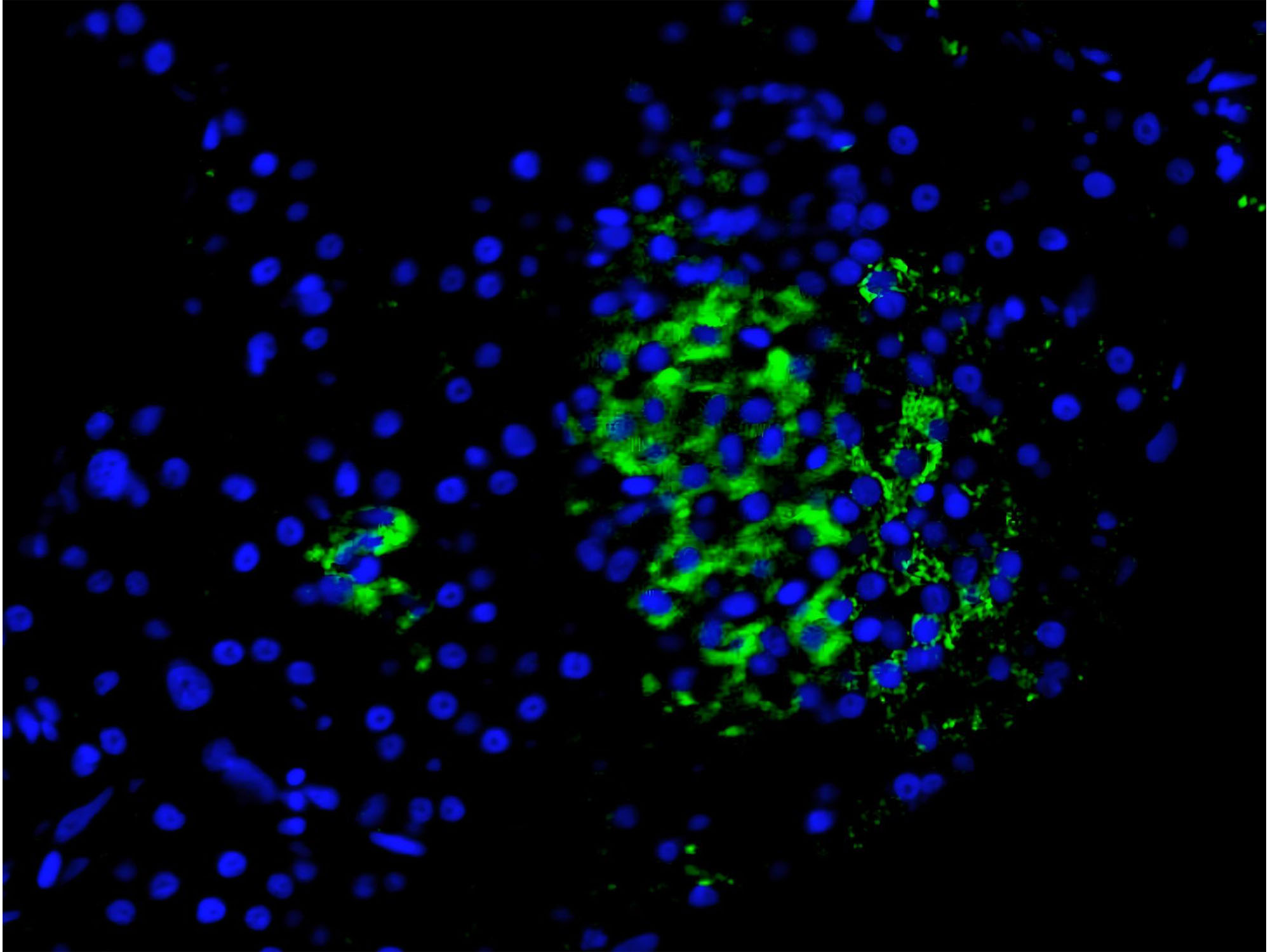

Supplement: Supplementary file 11 [file Data_Sheet_3.ZIP › IF-c-caspase/Model-1.jpg]

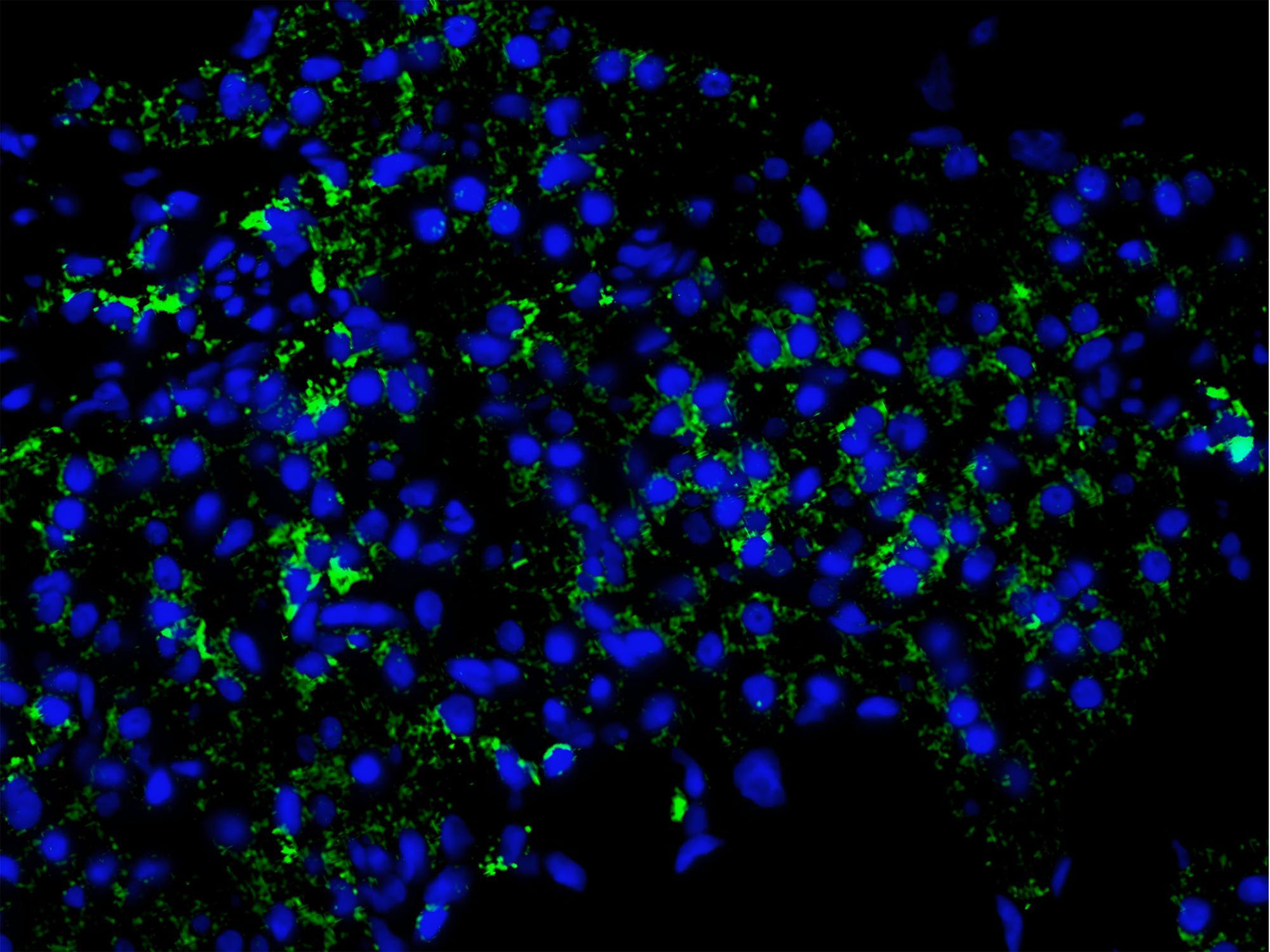

Supplement: Supplementary file 11 [file Data_Sheet_3.ZIP › IF-c-caspase/Model-2.jpg]

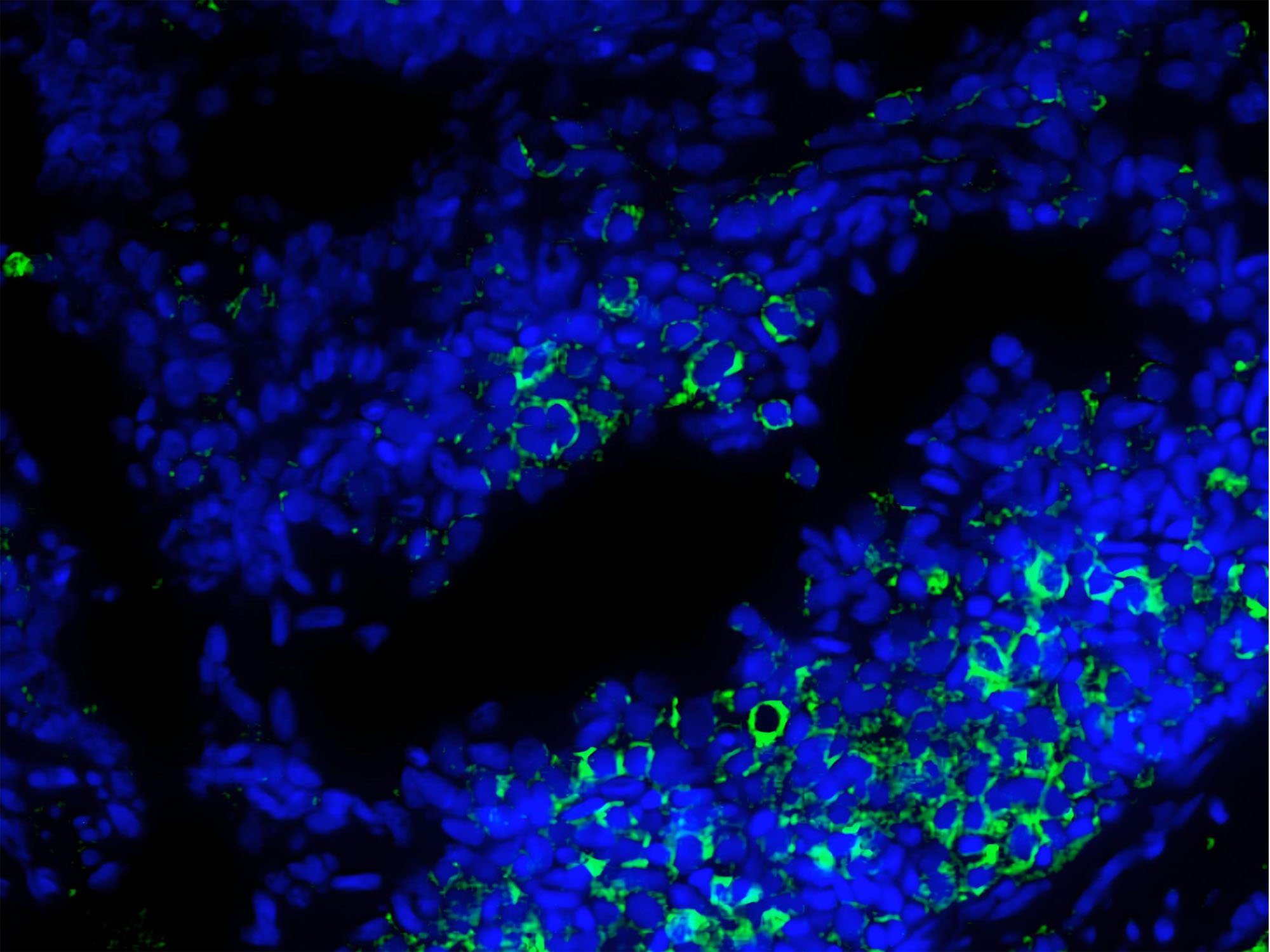

Supplement: Supplementary file 11 [file Data_Sheet_3.ZIP › IF-c-caspase/Model-3.jpg]

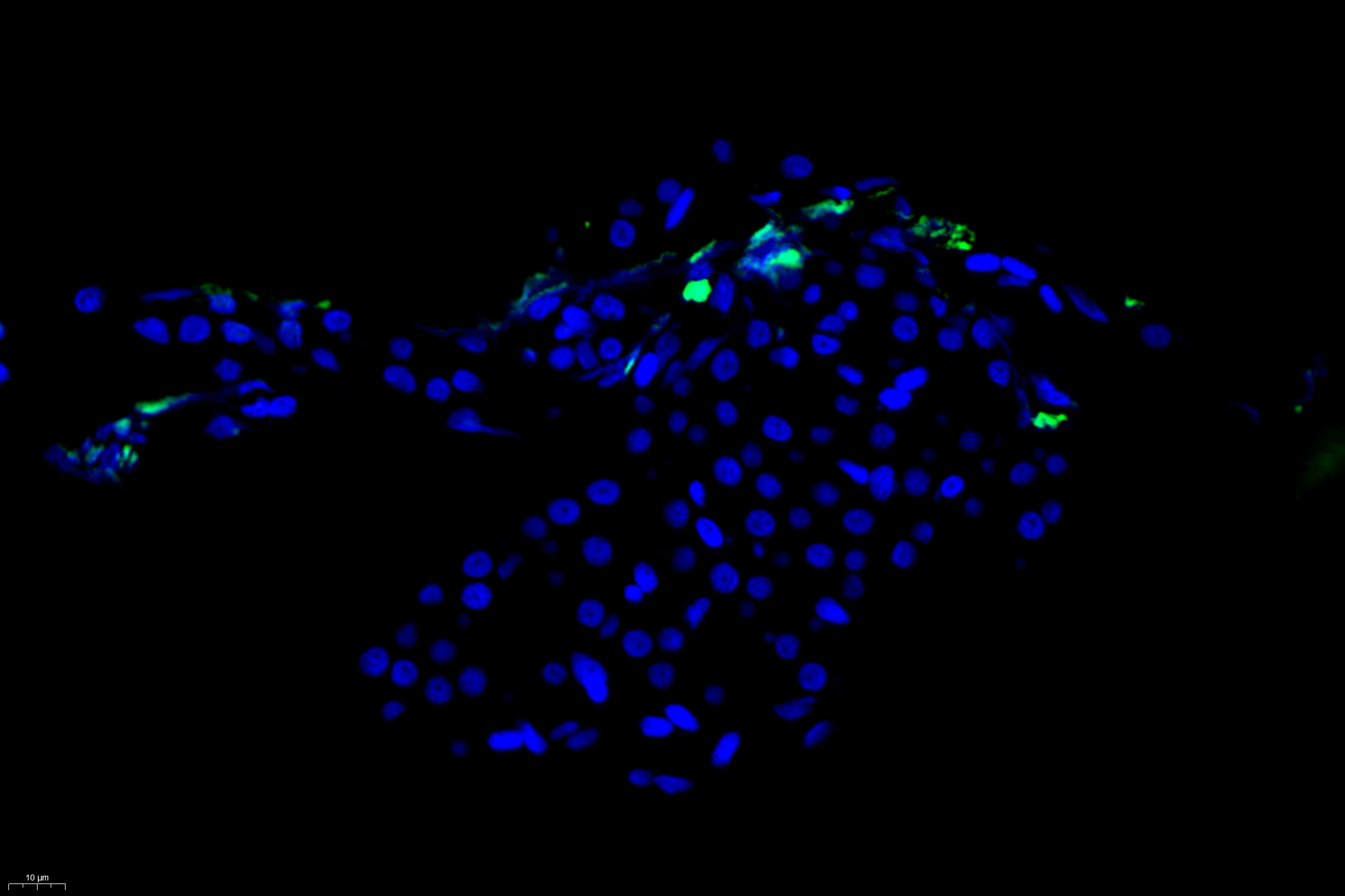

Supplement: Supplementary file 12 [file Data_Sheet_4.ZIP › TUNEL picture/100 PSP-1.jpg]

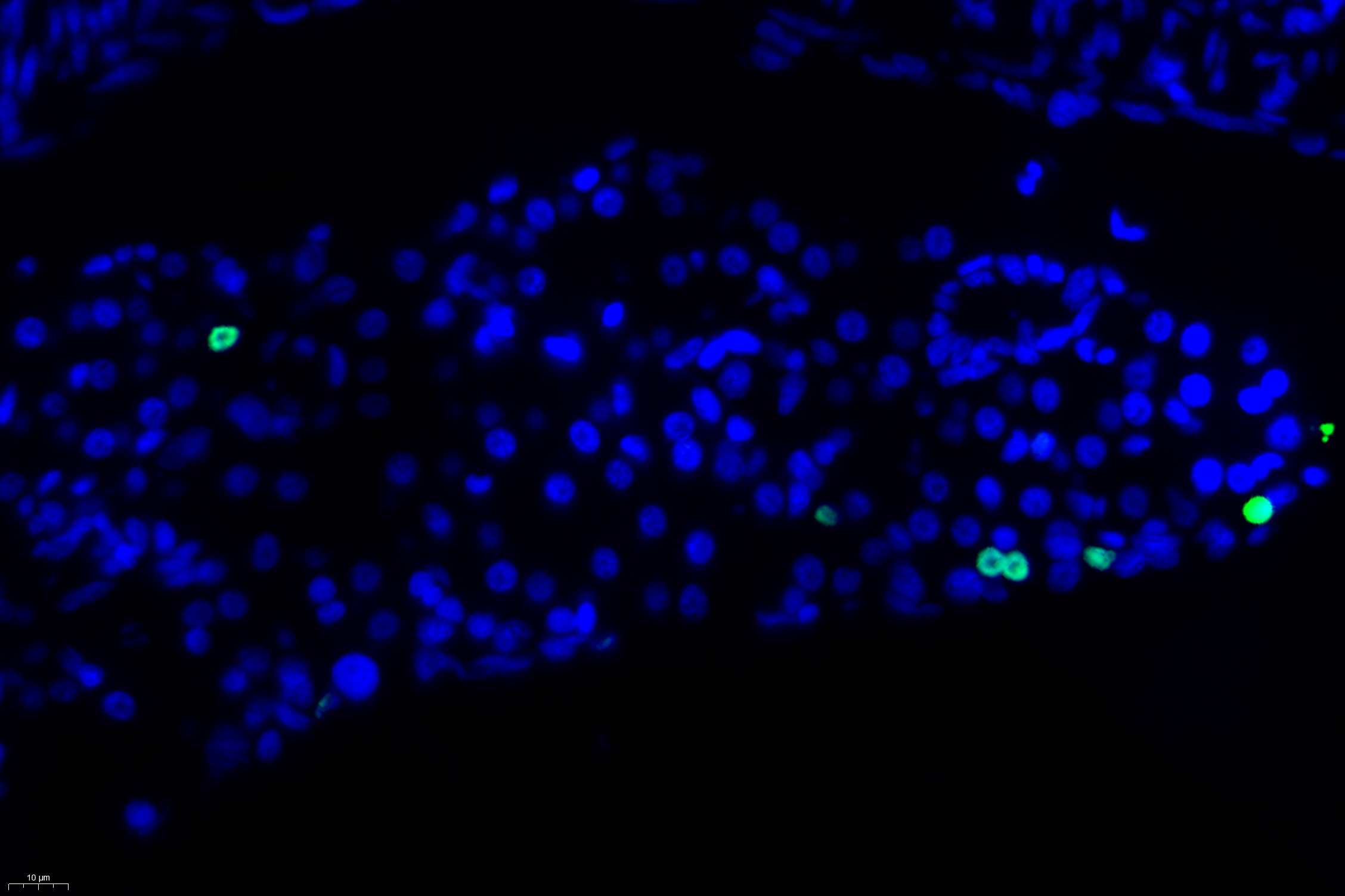

Supplement: Supplementary file 12 [file Data_Sheet_4.ZIP › TUNEL picture/100 PSP-2.jpg]

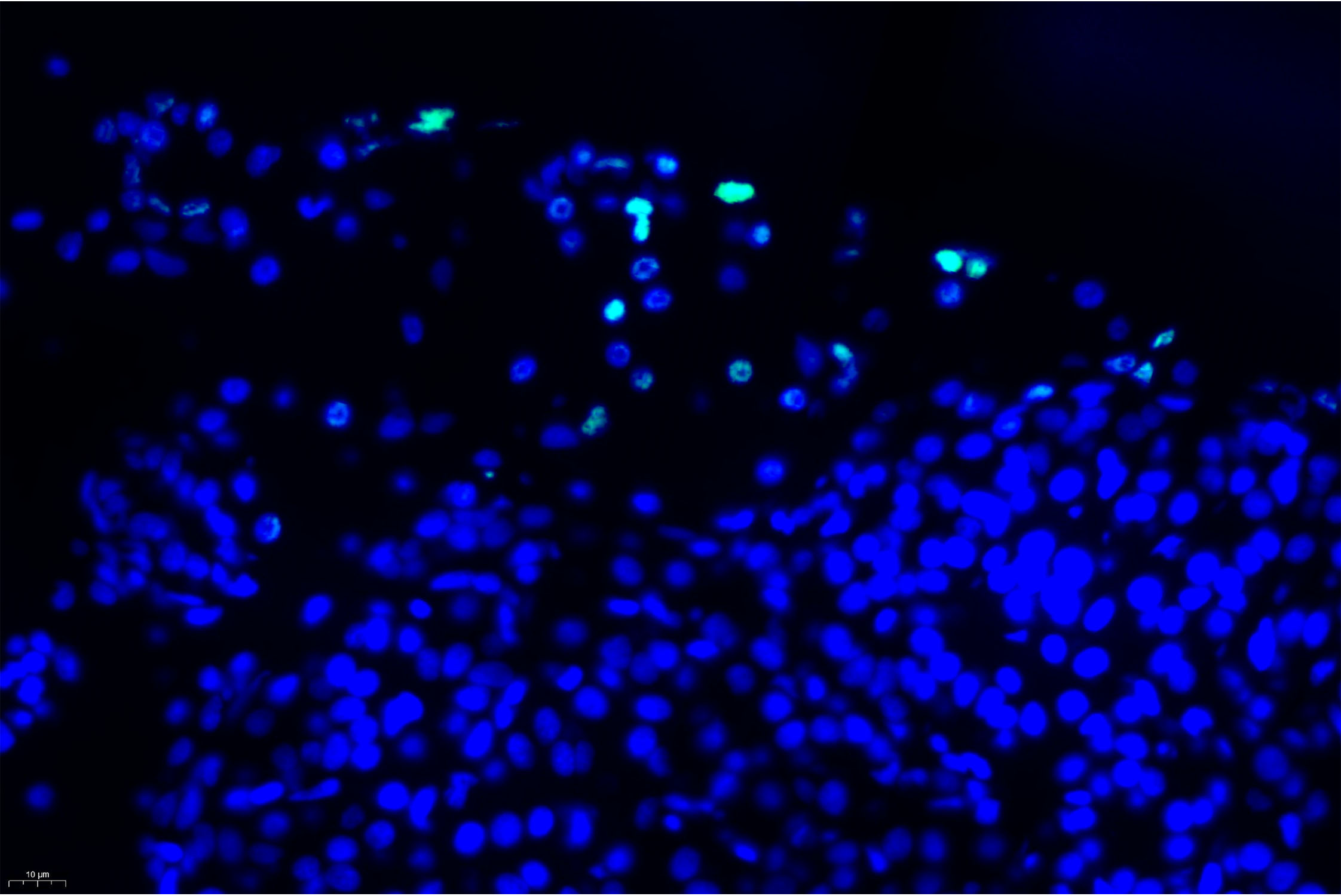

Supplement: Supplementary file 12 [file Data_Sheet_4.ZIP › TUNEL picture/100 PSP-3.jpg]

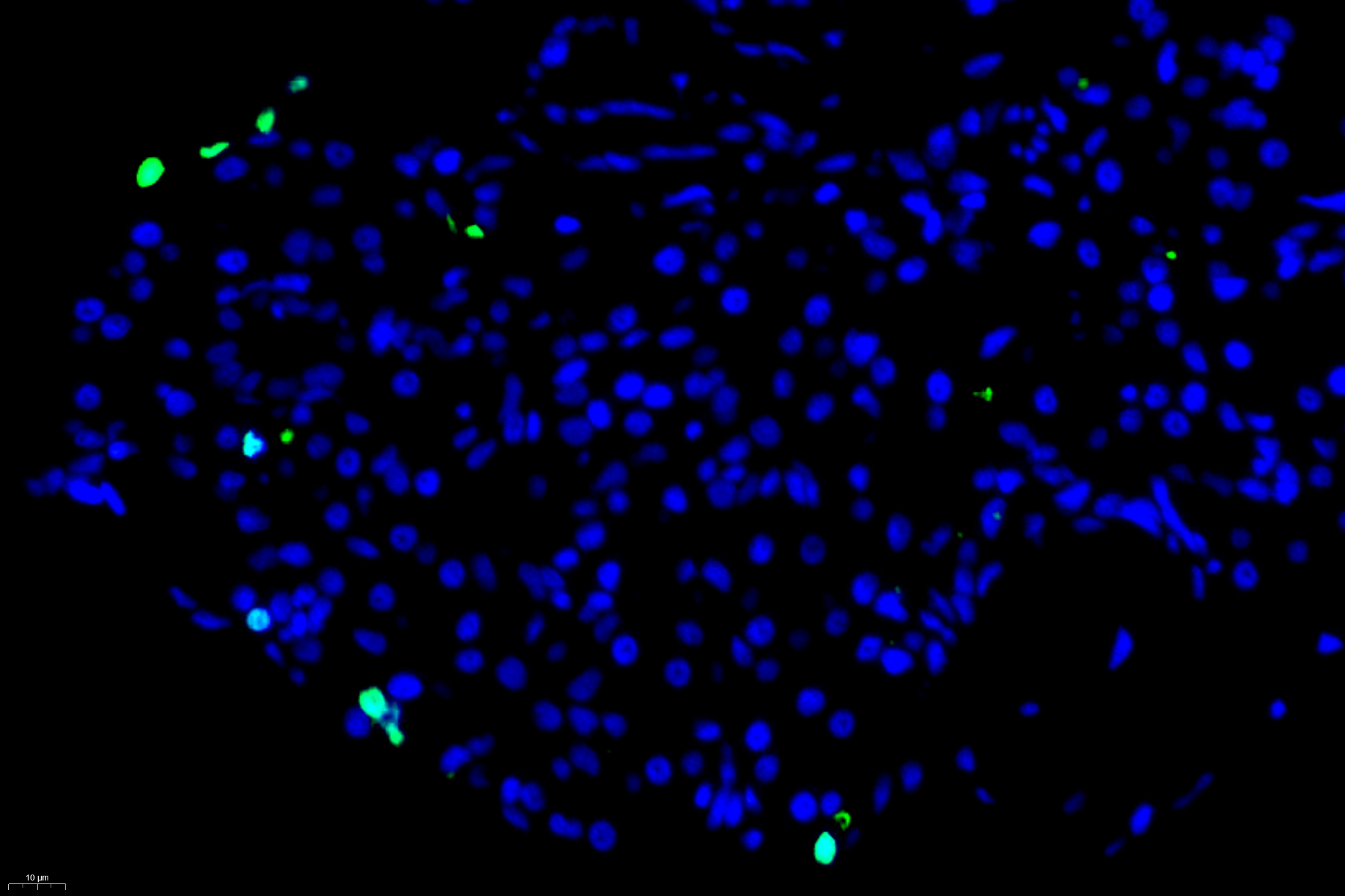

Supplement: Supplementary file 12 [file Data_Sheet_4.ZIP › TUNEL picture/200 PSP-1.jpg]

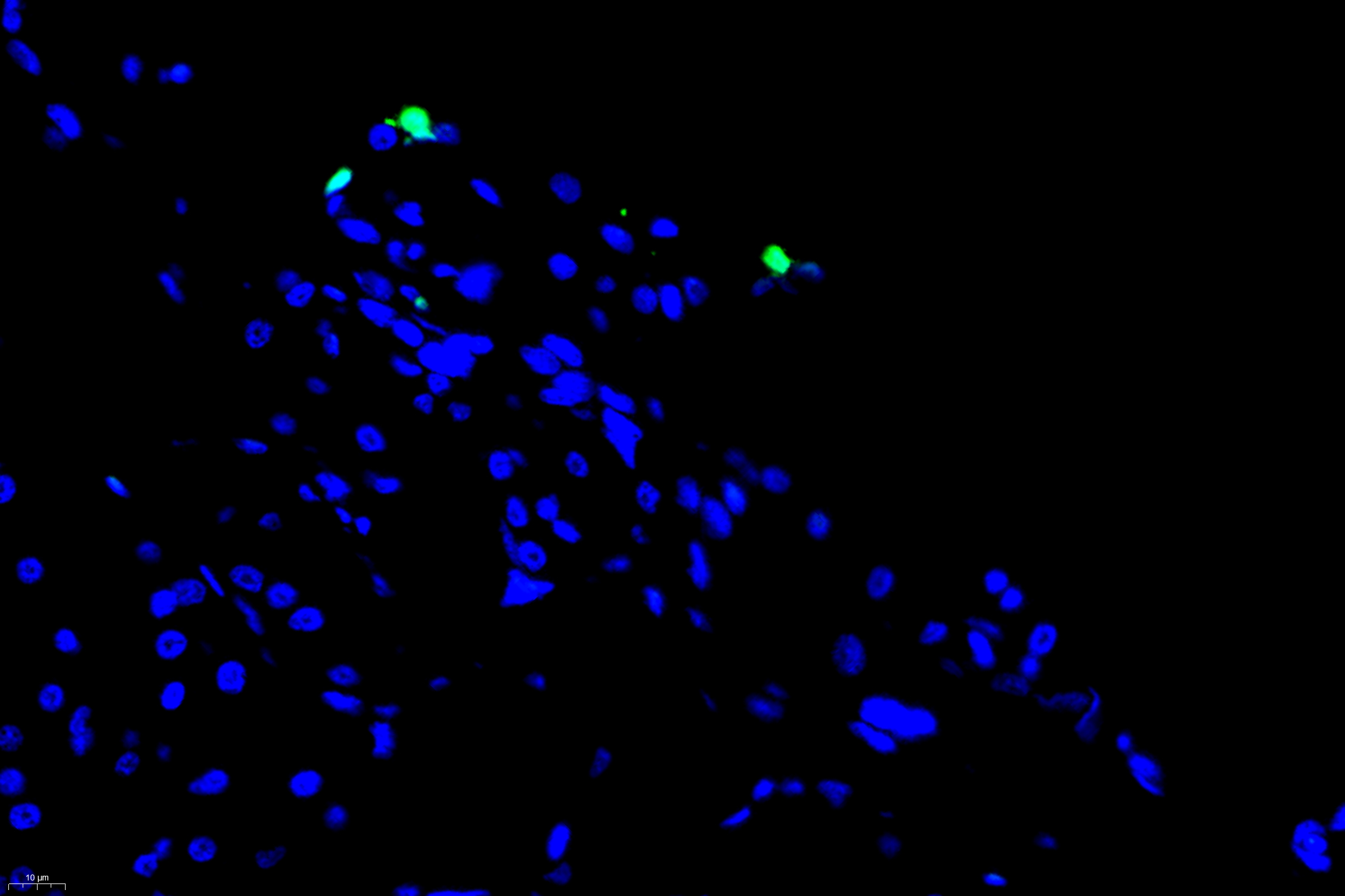

Supplement: Supplementary file 12 [file Data_Sheet_4.ZIP › TUNEL picture/200 PSP-2.jpg]

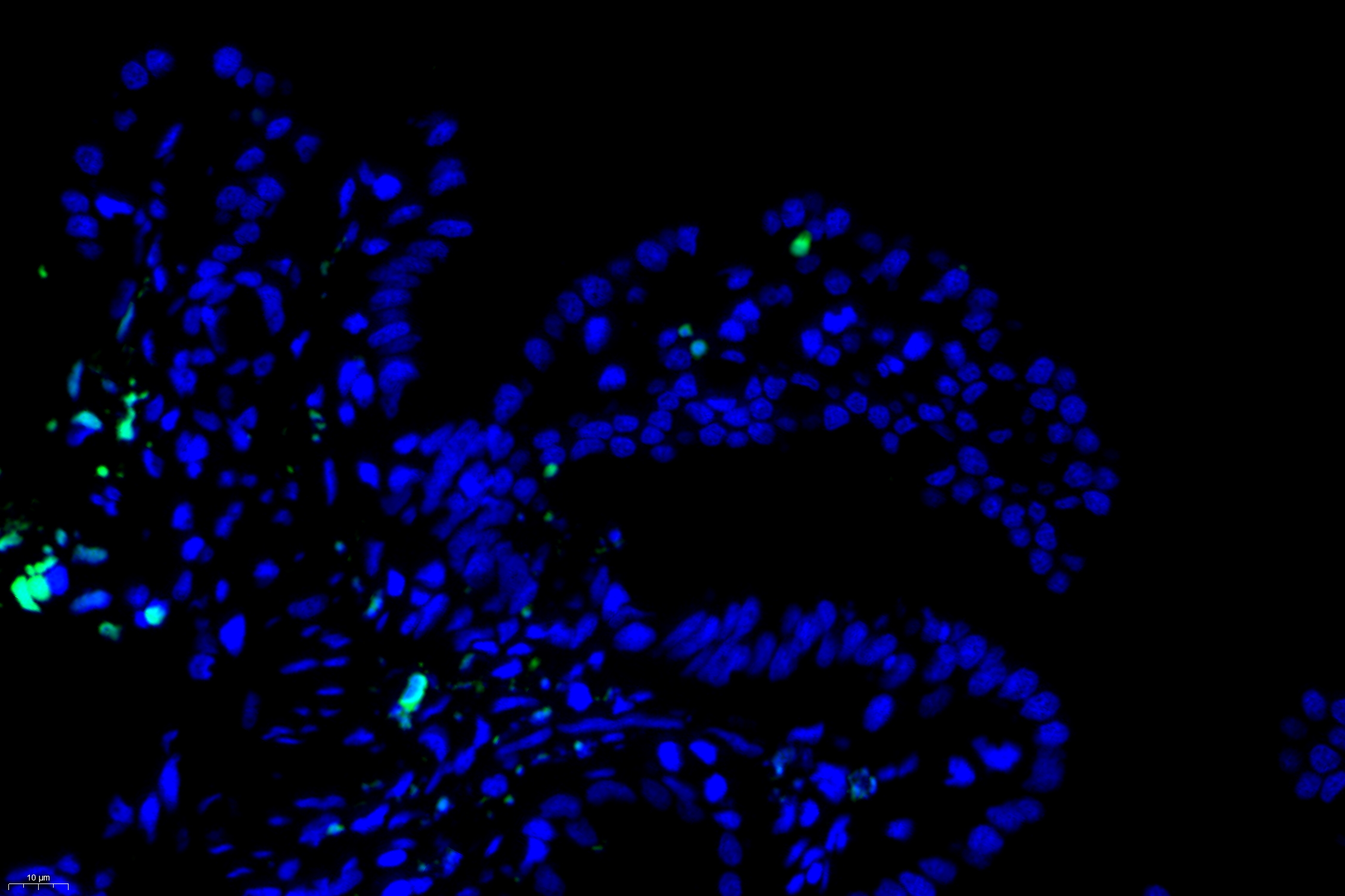

Supplement: Supplementary file 12 [file Data_Sheet_4.ZIP › TUNEL picture/200 PSP-3.jpg]

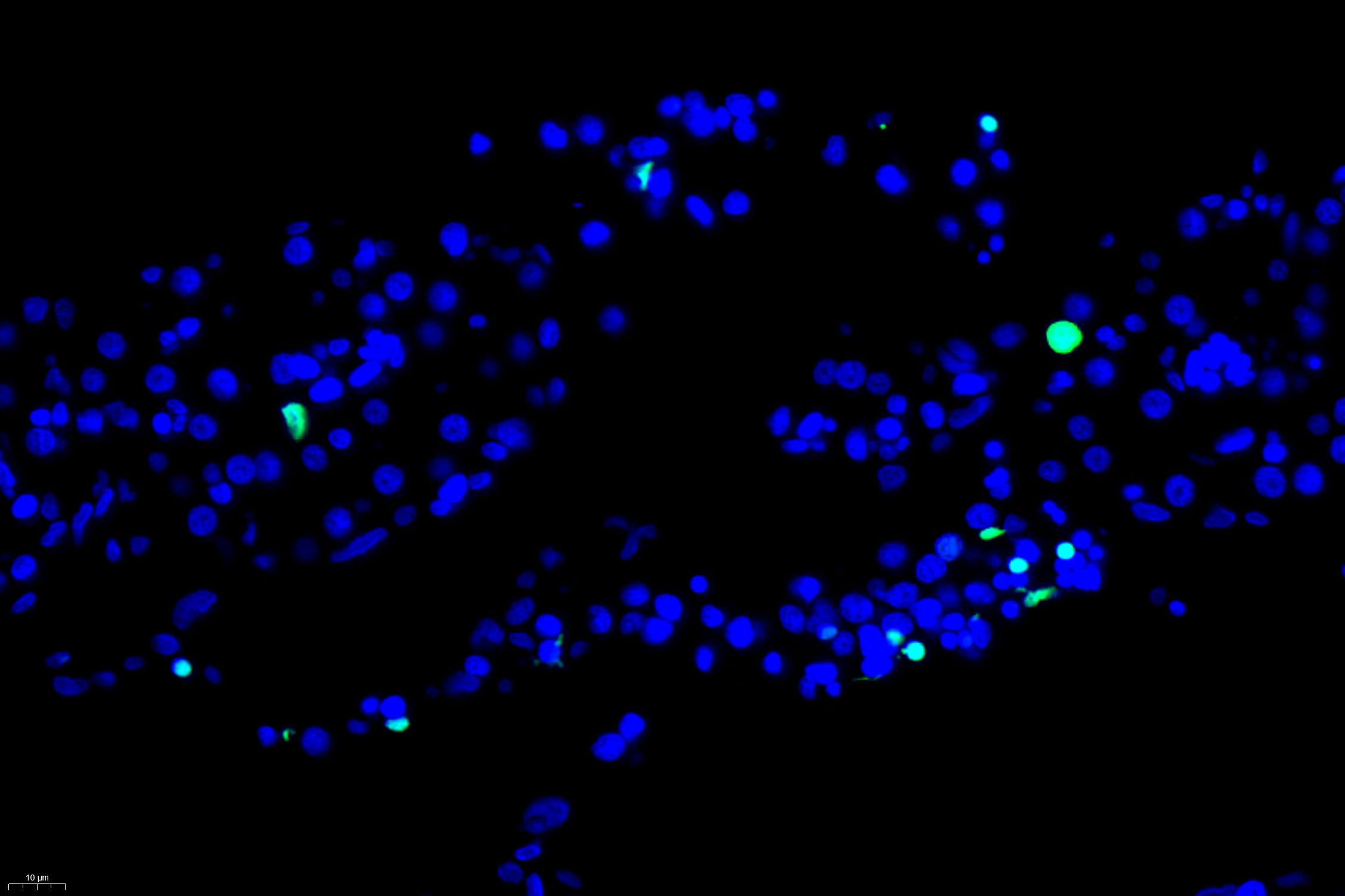

Supplement: Supplementary file 12 [file Data_Sheet_4.ZIP › TUNEL picture/50 PSP-1.jpg]

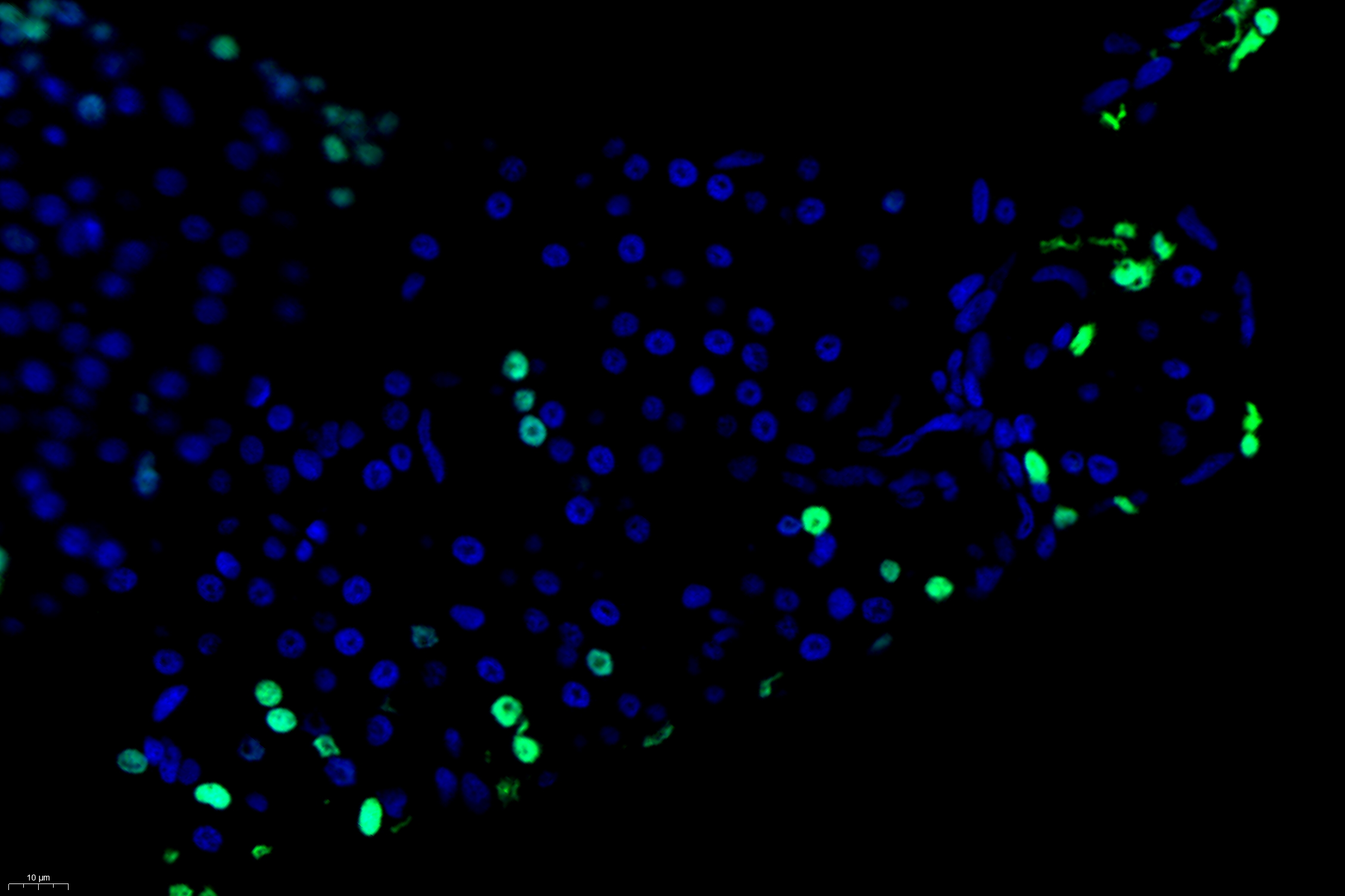

Supplement: Supplementary file 12 [file Data_Sheet_4.ZIP › TUNEL picture/50 PSP-2.jpg]

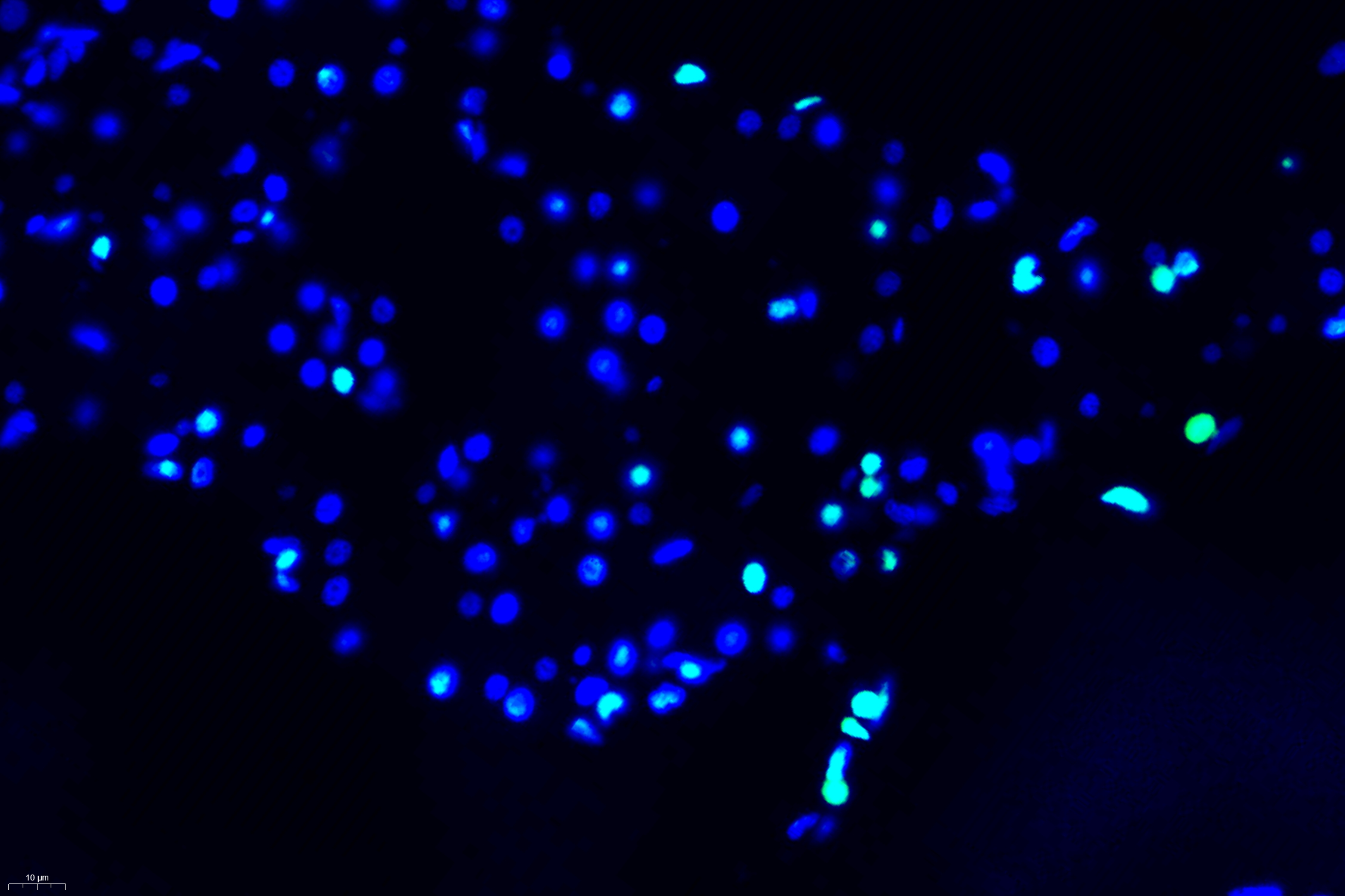

Supplement: Supplementary file 12 [file Data_Sheet_4.ZIP › TUNEL picture/50 PSP-3.jpg]

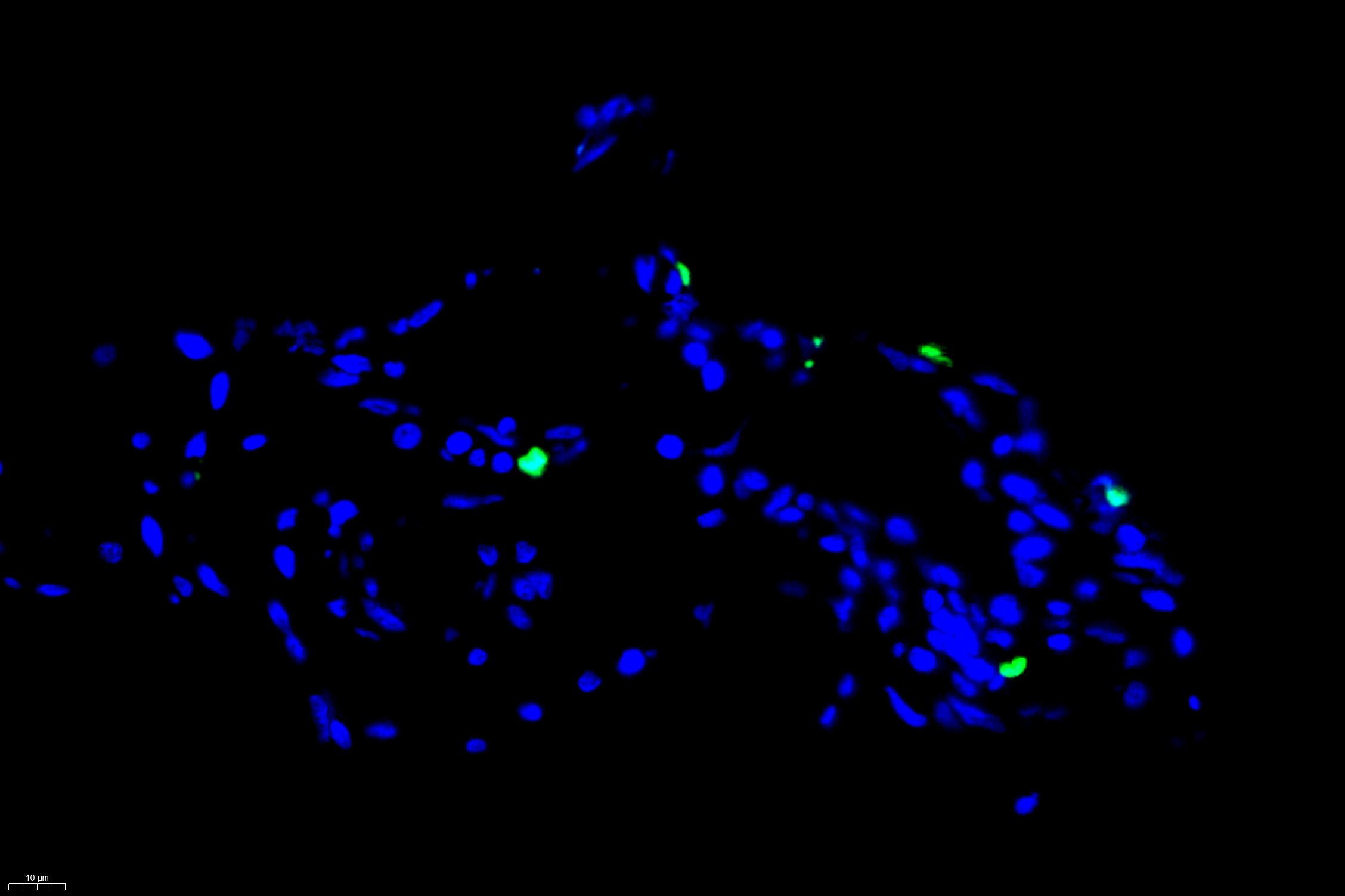

Supplement: Supplementary file 12 [file Data_Sheet_4.ZIP › TUNEL picture/Control-1.jpg]

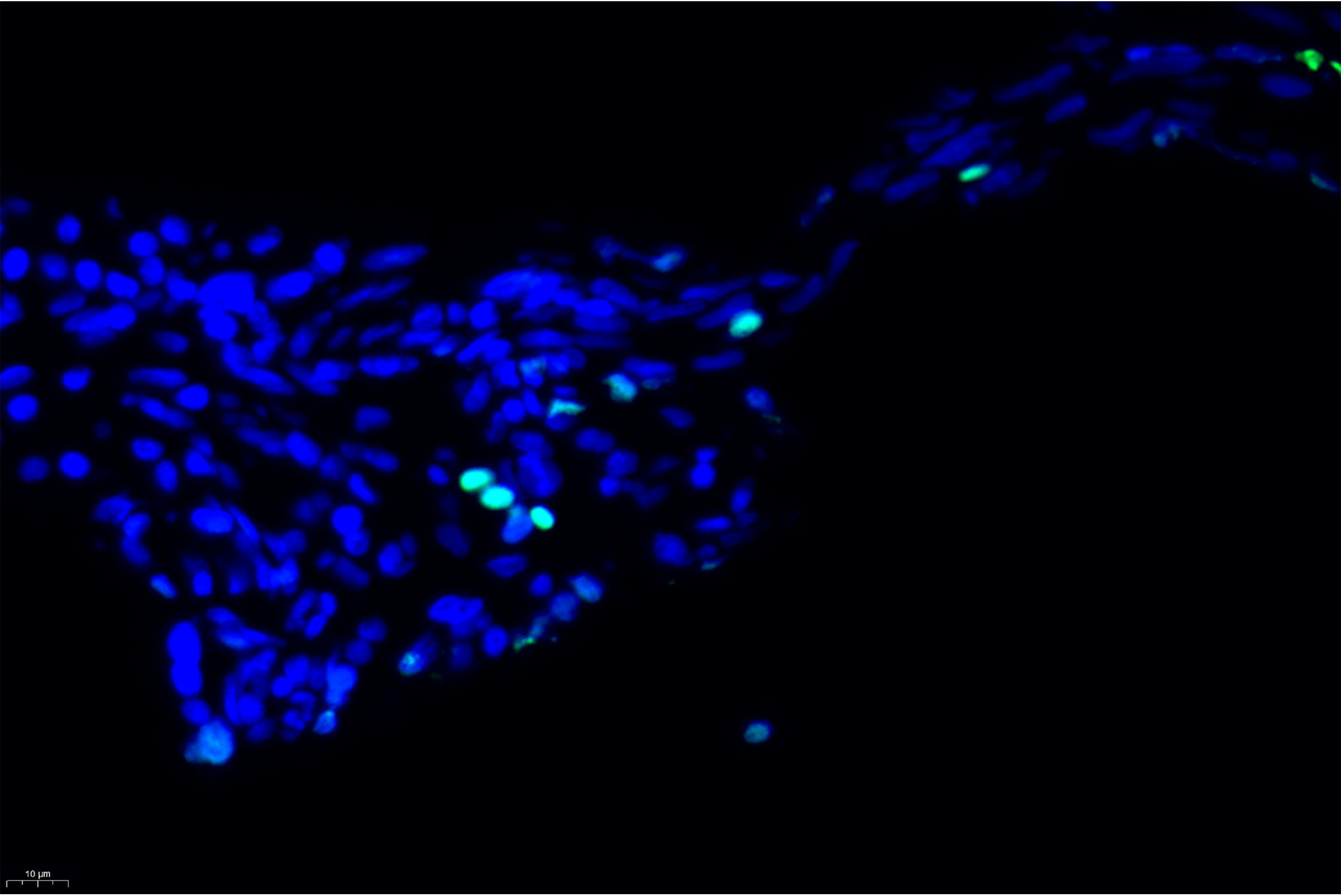

Supplement: Supplementary file 12 [file Data_Sheet_4.ZIP › TUNEL picture/Control-2.jpg]

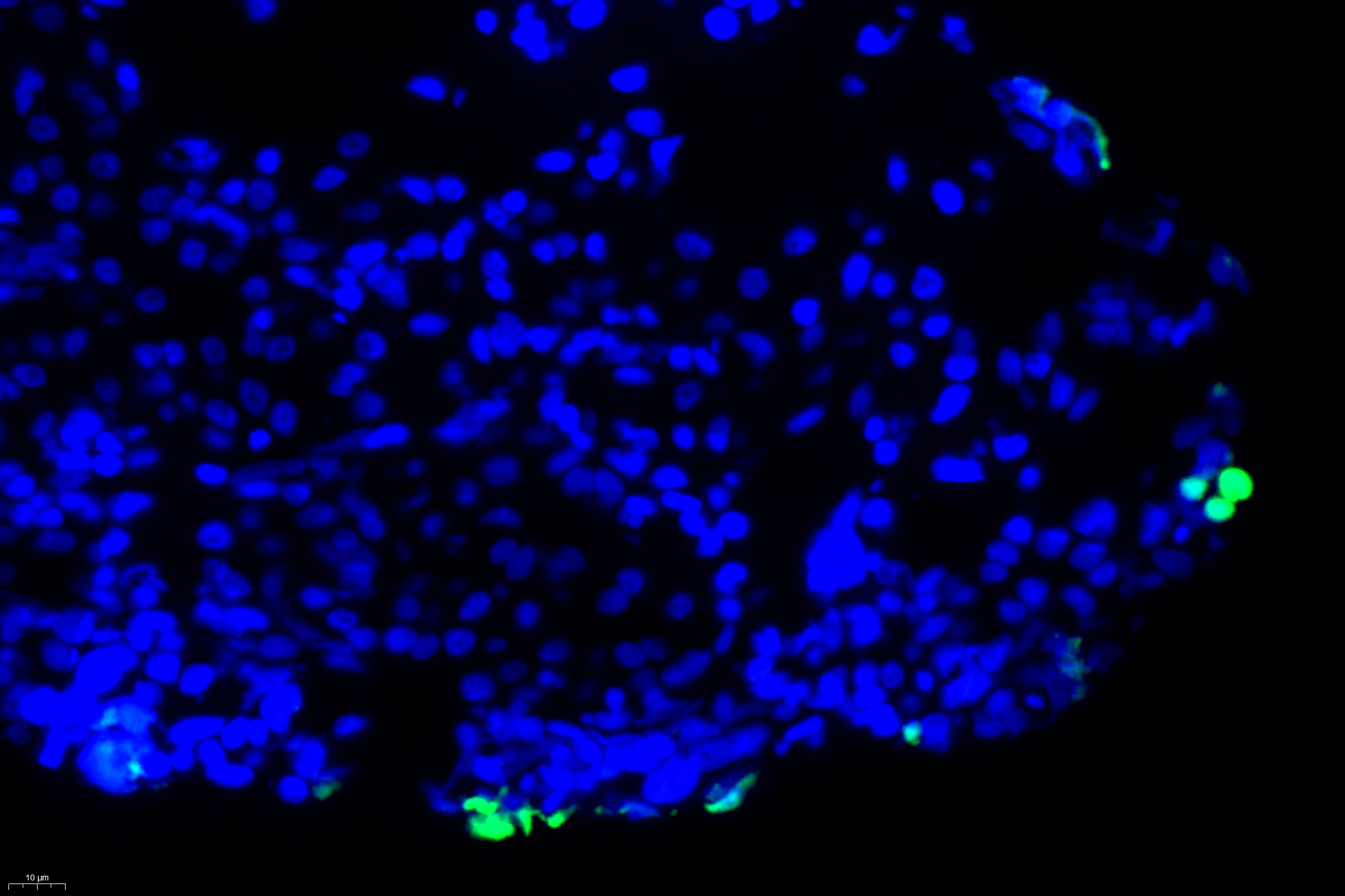

Supplement: Supplementary file 12 [file Data_Sheet_4.ZIP › TUNEL picture/Control-3.jpg]

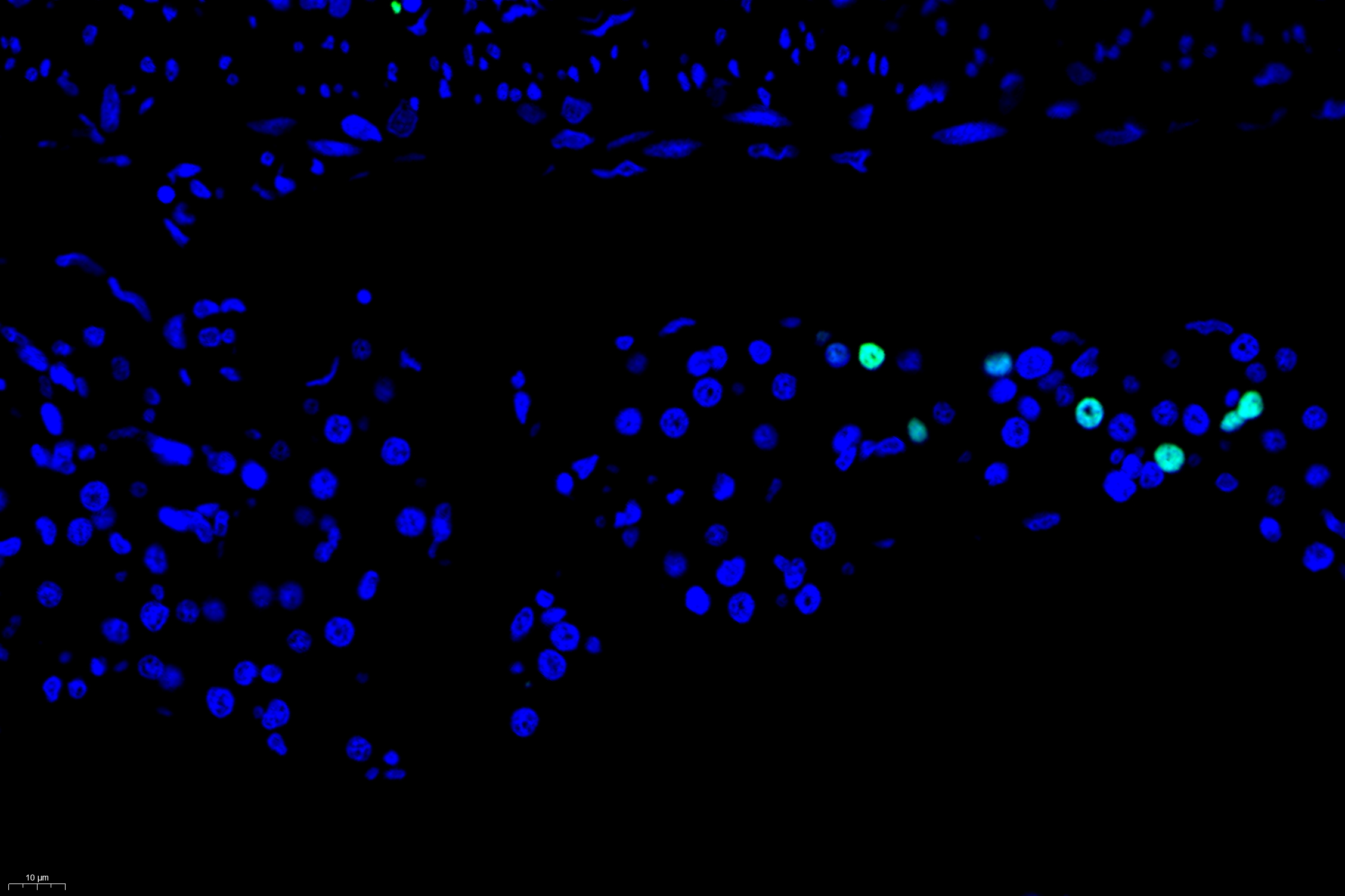

Supplement: Supplementary file 12 [file Data_Sheet_4.ZIP › TUNEL picture/Metformin-1.jpg]

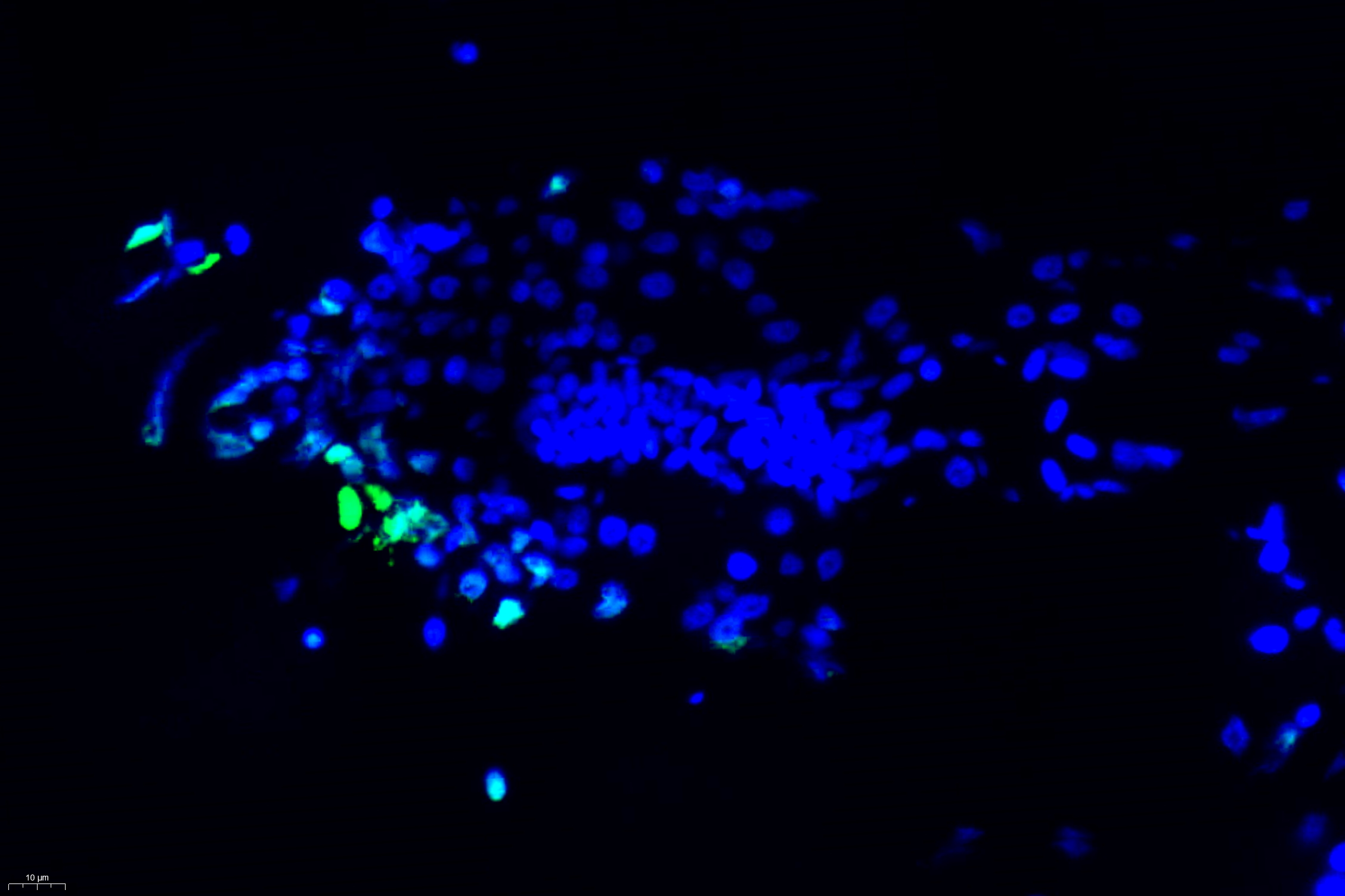

Supplement: Supplementary file 12 [file Data_Sheet_4.ZIP › TUNEL picture/Metformin-2.jpg]

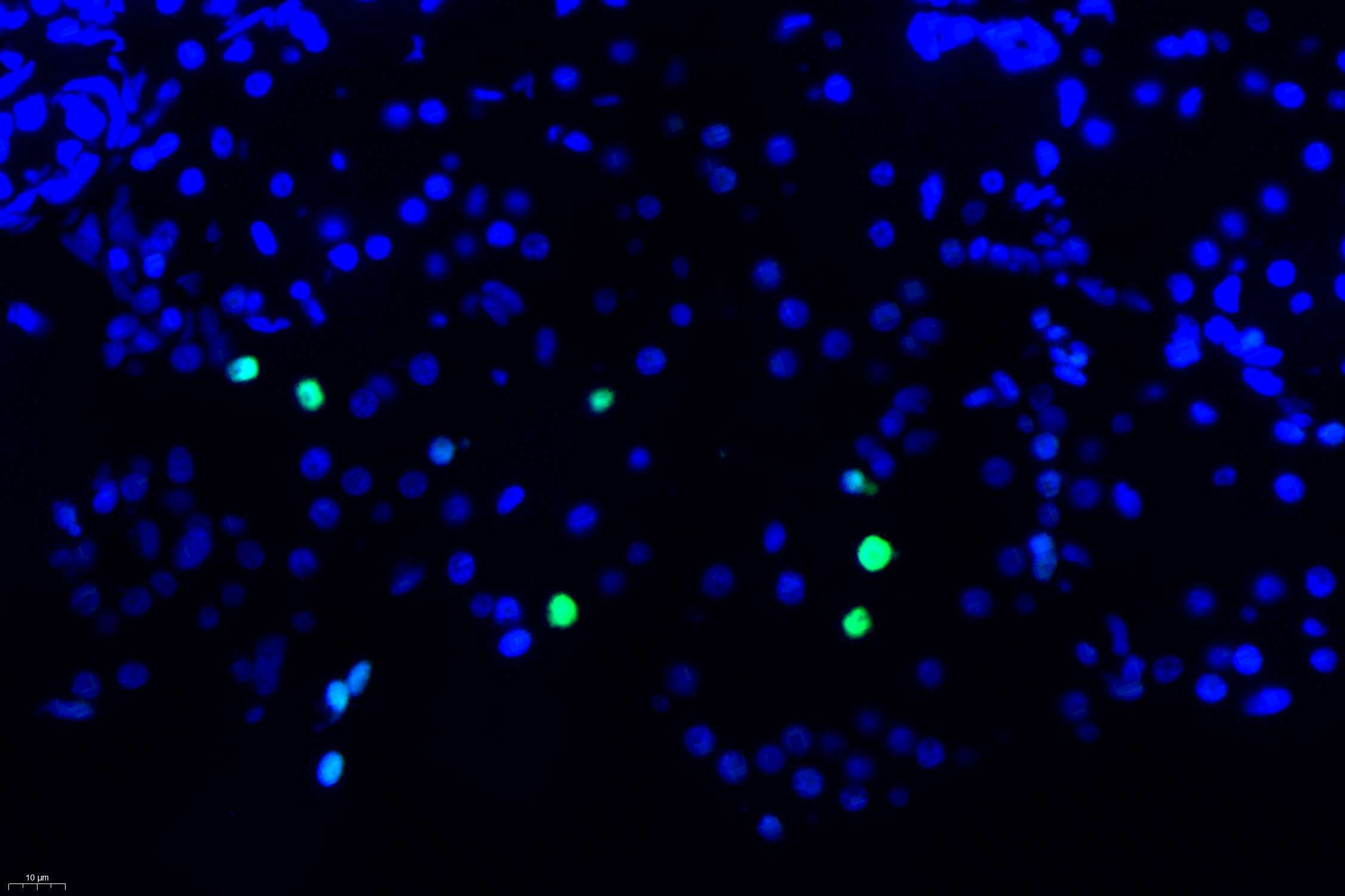

Supplement: Supplementary file 12 [file Data_Sheet_4.ZIP › TUNEL picture/Metformin-3.jpg]

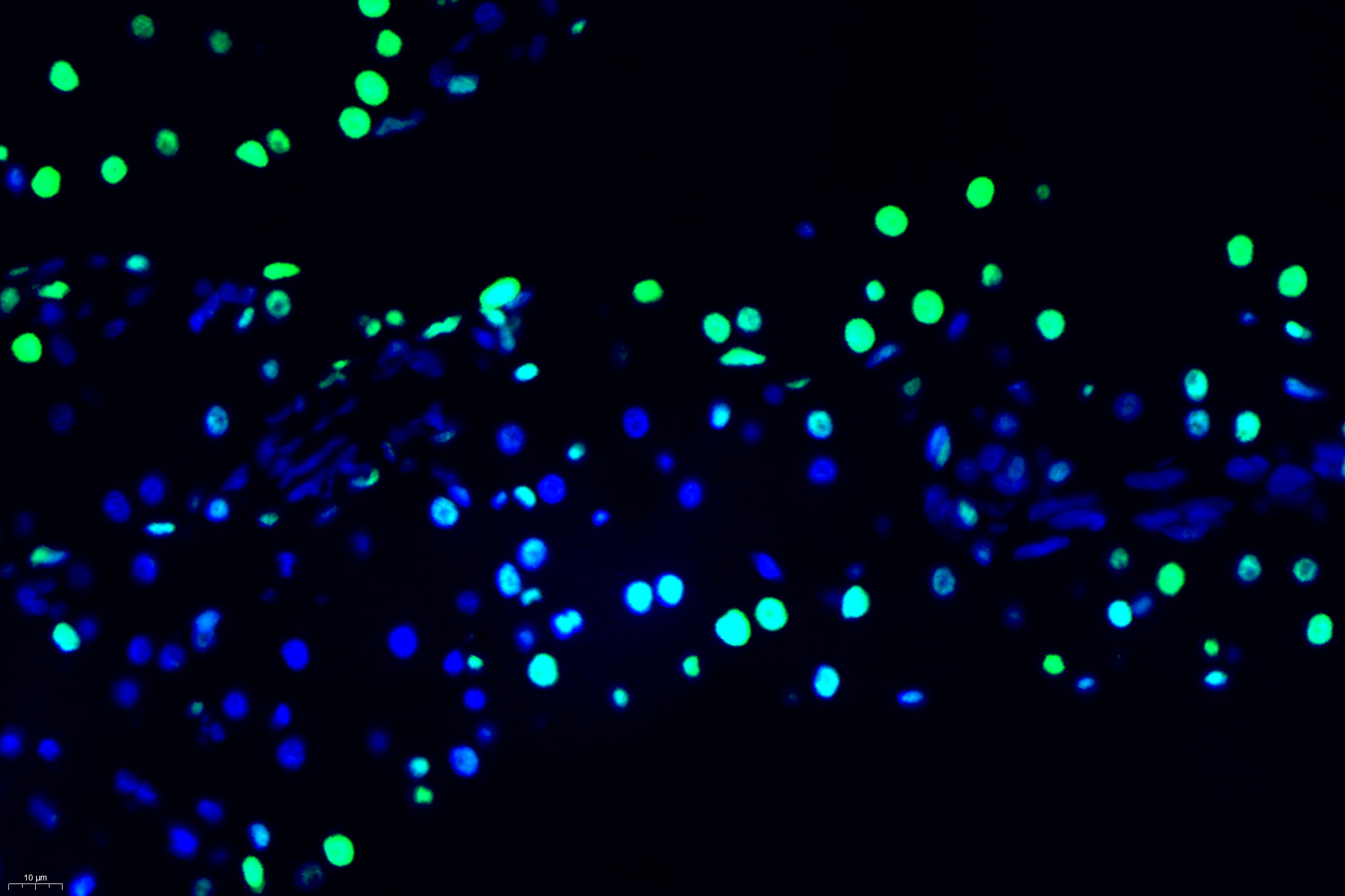

Supplement: Supplementary file 12 [file Data_Sheet_4.ZIP › TUNEL picture/Model-1.jpg]

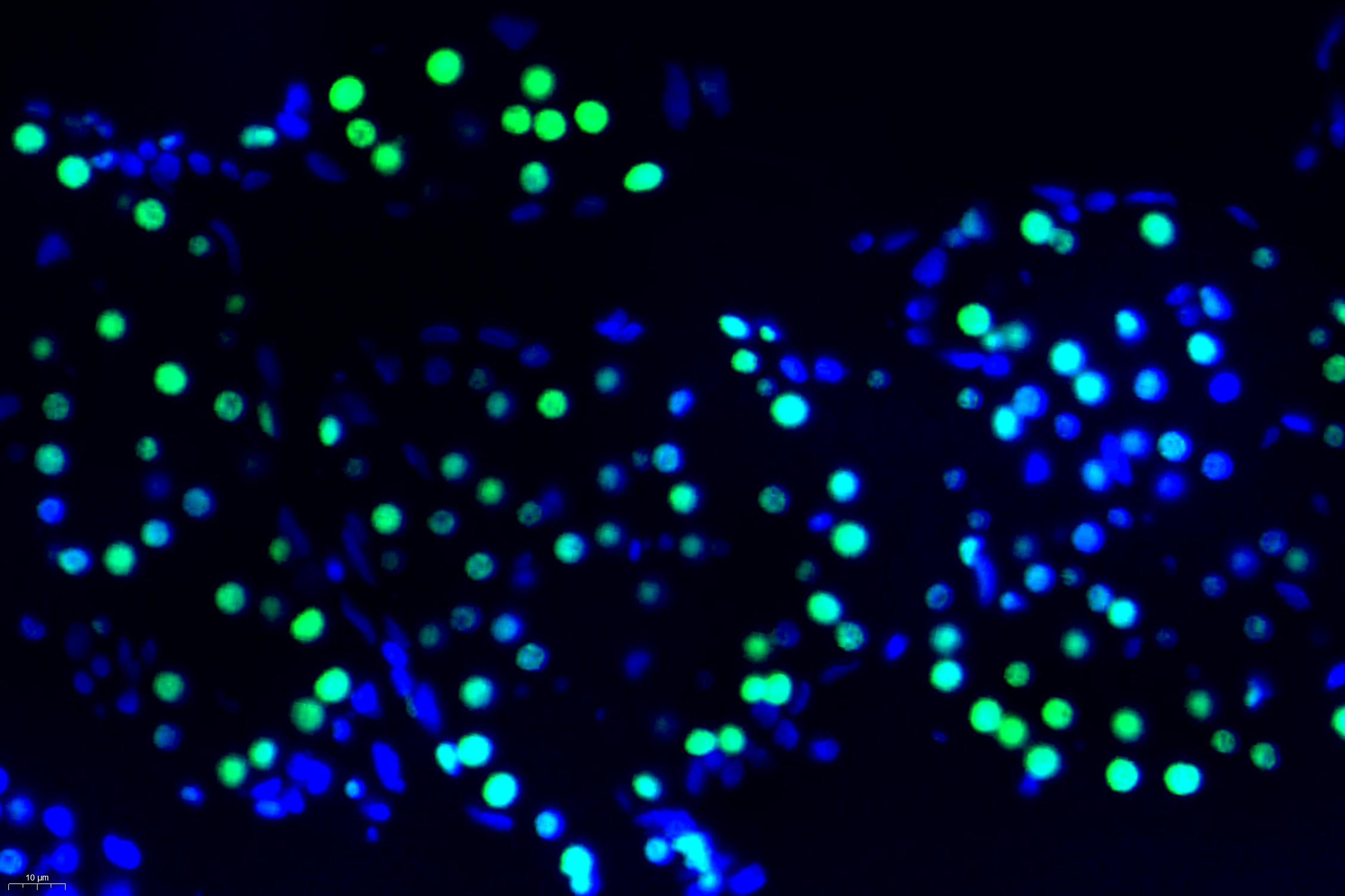

Supplement: Supplementary file 12 [file Data_Sheet_4.ZIP › TUNEL picture/Model-2.jpg]

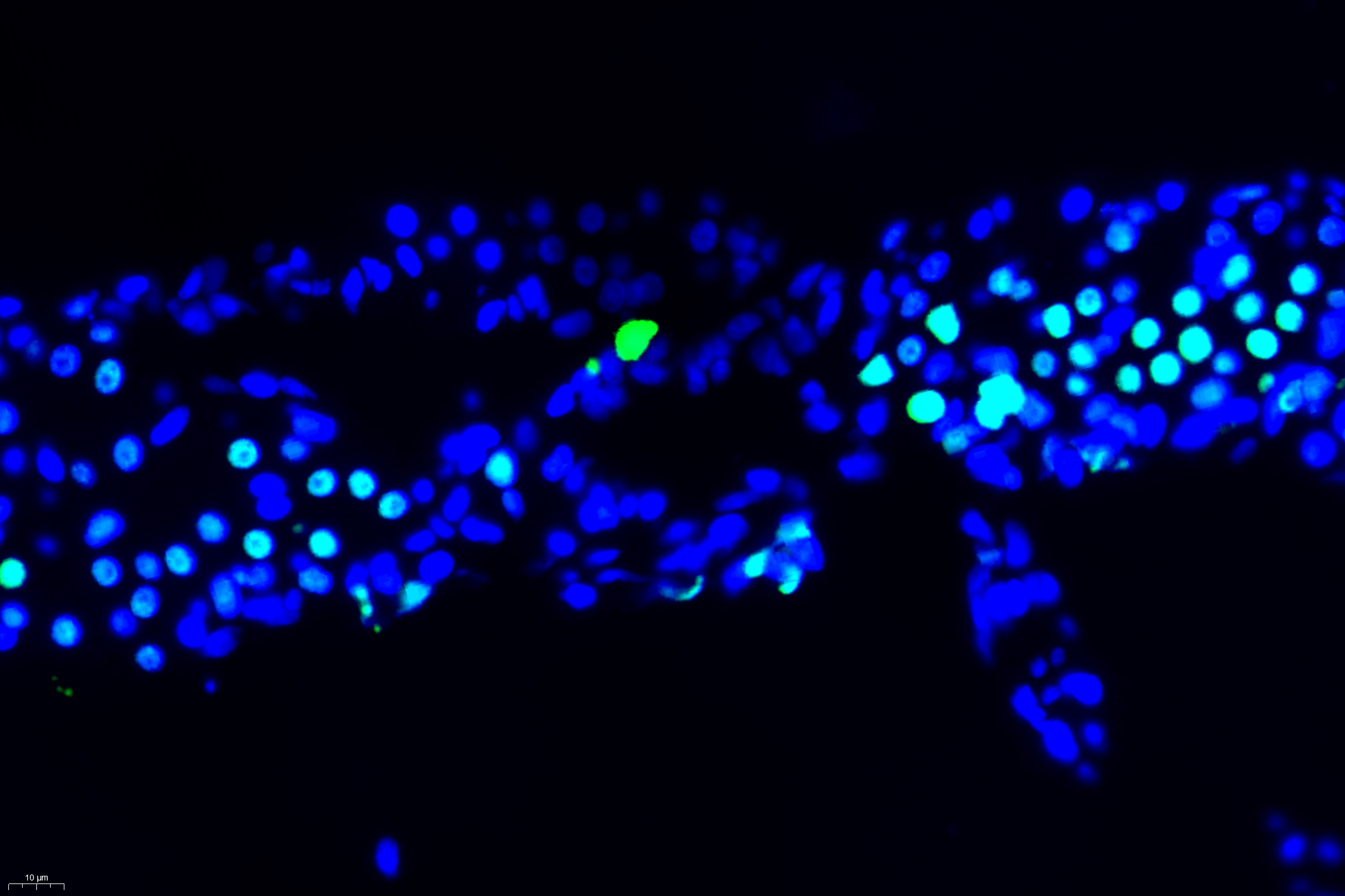

Supplement: Supplementary file 12 [file Data_Sheet_4.ZIP › TUNEL picture/Model-3.jpg]
