# Supplementary material for: Polygonatum sibiricum polysaccharides enhance pancreatic β-cell function in diabetic zebrafish by mitigating mitochondrial oxidative damage via the AMPK-SIRT1 pathway
Source: Front Nutr. 2025 May 9;12:1601490. doi: 10.3389/fnut.2025.1601490 (PMC12128605; doi:10.3389/fnut.2025.1601490)
Supplement: Supplementary file 9 [file Data_Sheet_1.PDF]

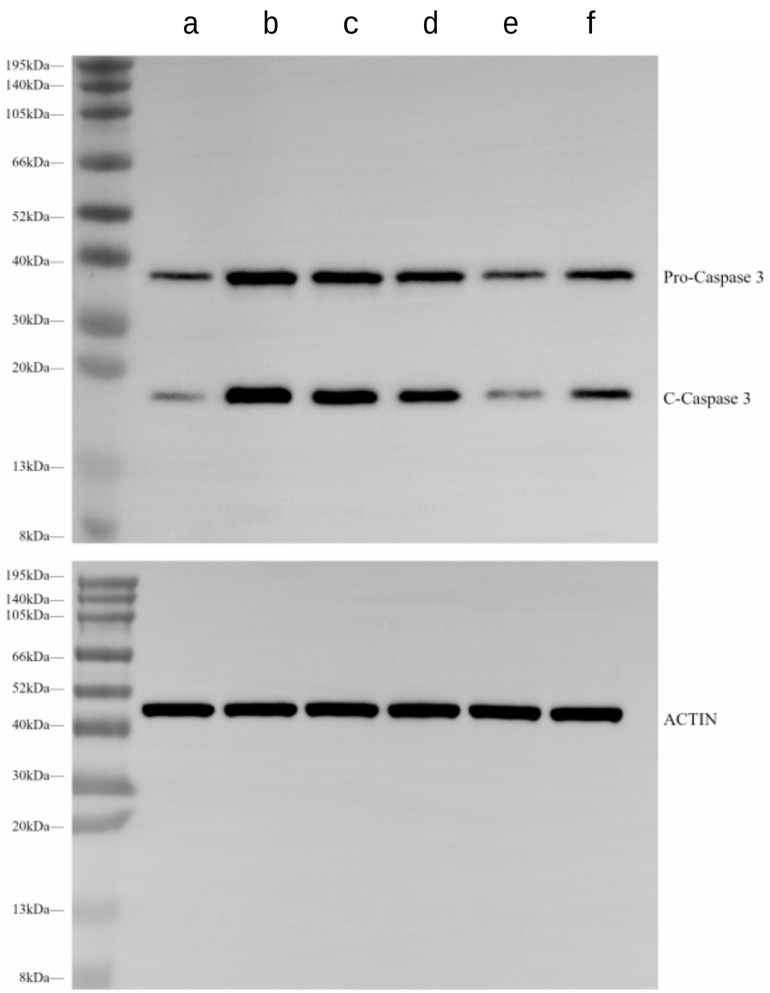

a: Control group, b: Model group, c: 50 μg/mL PSP group, d: 100 μg/mL PSP group, e: 200 μg/mL PSP group, f: Metformin group.

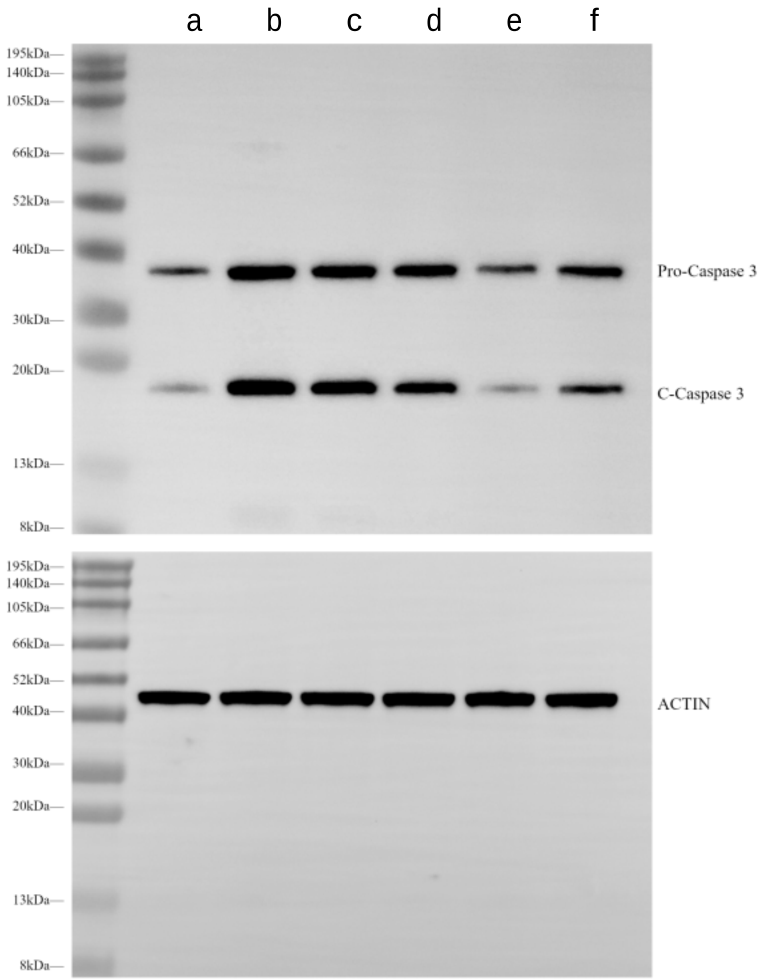

a: Control group, b: Model group, c: 50 μg/mL PSP group, d: 100 μg/mL PSP group, e: 200 μg/mL PSP group, f: Metformin group.

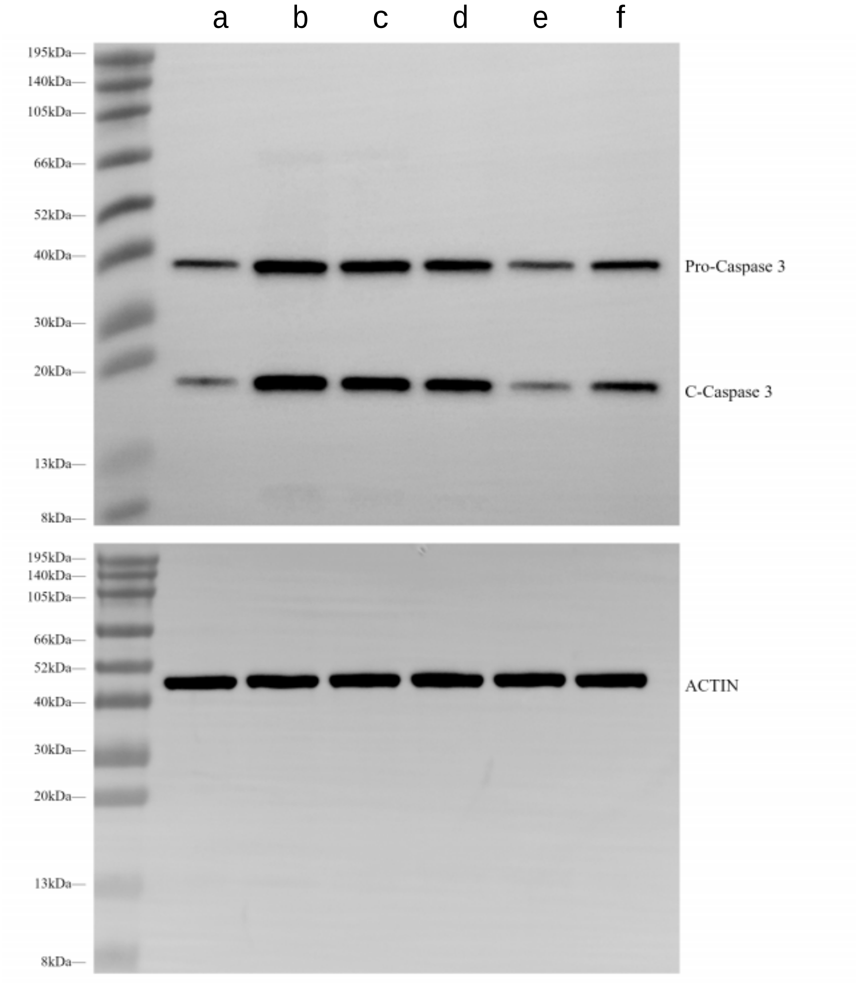

a: Control group, b: Model group, c: 50 μg/mL PSP group, d: 100 μg/mL PSP group, e: 200 μg/mL PSP group, f: Metformin group.
